# Speciation and Introgression between *Mimulus nasutus* and *Mimulus guttatus*

**DOI:** 10.1371/journal.pgen.1004410

**Published:** 2014-06-26

**Authors:** Yaniv Brandvain, Amanda M. Kenney, Lex Flagel, Graham Coop, Andrea L. Sweigart

**Affiliations:** 1 Department of Evolution and Ecology & Center for Population Biology, University of California - Davis, Davis, California, United States of America; 2 Department of Plant Biology, University of Minnesota – Twin Cities. St. Paul, Minnesota, United States of America; 3 Department of Genetics, University of Georgia, Athens, Georgia, United States of America; 4 Monsanto Company, Chesterfield, Missouri, United States of America; University of Cambridge, United Kingdom

## Abstract

*Mimulus guttatus* and *M. nasutus* are an evolutionary and ecological model sister species pair differentiated by ecology, mating system, and partial reproductive isolation. Despite extensive research on this system, the history of divergence and differentiation in this sister pair is unclear. We present and analyze a population genomic data set which shows that *M. nasutus* budded from a central Californian *M. guttatus* population within the last 200 to 500 thousand years. In this time, the *M. nasutus* genome has accrued genomic signatures of the transition to predominant selfing, including an elevated proportion of nonsynonymous variants, an accumulation of premature stop codons, and extended levels of linkage disequilibrium. Despite clear biological differentiation, we document genomic signatures of ongoing, bidirectional introgression. We observe a negative relationship between the recombination rate and divergence between *M. nasutus* and sympatric *M. guttatus* samples, suggesting that selection acts against *M. nasutus* ancestry in *M. guttatus*.

## Author Summary

While speciation is often depicted as a simple population split, in many cases it is likely more complex. Recently, whole genome sequencing and computational methods to interpret patterns of genomic variation have facilitated the inference of complex speciation histories. We present and analyze genomic data to infer the speciation history of an ecological and evolutionary model species pair - *Mimulus guttatus*/*M. nasutus*. We infer that *M. nasutus* split from a central Californian *M. guttatus* population approximately 200–500 kya, roughly corresponding to *M. nasutus*’ shift to self-fertilization. We document ongoing gene flow between these species where they co-occur. Finally, we present patterns genomic divergence suggesting that natural selection disfavors introgression of *M. nasutus* ancestry in *M. guttatus*.

## Introduction

While speciation is often depicted as a simple event in which a single species splits into two, there is increasing evidence that this process is often more complex. In particular, speciation reflects a tension among divergence, the assortment of ancestral variation, ecological interactions and in some cases introgression that play out across the environment of the incipient species. Historically, a population genetic view of the process of speciation has been limited to few loci, where stochasticity in ancestral processes can prevent strong inferences about isolation and gene flow. By contrast, whole genome resequencing (even of only a few individuals) reveals many genealogical histories across contiguous genomic regions to provide well-resolved views of population history, divergence and introgression [1]–[5]. Here, we present a population genomic investigation of the speciation history of two closely related species of yellow monkeyflowers, the primarily outcrossing *Mimulus guttatus*, and the self-pollinating *M. nasutus –* an evolutionary model system for which the genetic and ecological basis of reproductive isolation is reasonably well characterized [6].

In flowering plants, speciation often involves a shift in pollinator (*e.g.*, [7]–[9]) or mating system (*e.g.*, [7], [10]–[12]), with concomitant divergence in key floral traits causing reproductive isolation between lineages. The evolutionary transition from outcrossing to self-fertilization, as occurred in *M. nasutus*, is of particular interest because the expected reduction in both the effective population size and effective recombination rate [13], [14] can dramatically alter population genetic processes and patterns of genomic variation [15], [16]. Recent evidence for elevated levels of putatively deleterious alleles in selfing taxa [17]–[19] is consistent with the idea that inbreeding reduces the effectiveness of purifying selection (due to a lowered effective population size). However, we still have few examples of the effects of self-fertilization on patterns of diversity across the genome, particularly in the context of recently diverged and potentially hybridizing species. Genomic datasets from young selfing species can uniquely inform the process of mating system divergence by allowing us to compare regions of the genome that share a common ancestor before or after the origin of self-fertilization and thus understand the assortment of ancestral variation [20].

The *M. guttatus – M. nasutus* species pair is an excellent model for investigating the causes and consequences of mating system evolution and species divergence. *M. guttatus* is primarily outcrossing (although the outcrossing rate varies across populations [21]–[23]) with large, bee-pollinated flowers and occupies diverse ecological habitats throughout western North America. *M. nasutus* is highly selfing with reduced, mostly closed flowers. Although these species are often found in different microhabitats, the range of *M. nasutus* is broadly nested within that of *M. guttatus* and the two species do co-occur. In sympatry, *M. nasutus* and *M. guttatus* are partially reproductively isolated by differences in floral morphology, flowering phenology, and pollen-pistil interactions [24]–[26]. Although early-generation hybrids occur in nature [24], [27], numerous intrinsic hybrid incompatibilities decrease hybrid fitness [28]–[30]. Based on the most detailed population genetic analyses of *Mimulus* to date (two and six nuclear loci, respectively [30], [31]), *M. nasutus* exhibits reduced diversity compared to *M. guttatus*, and some *M. guttatus* sequences are nearly identical to *M. nasutus*, suggestive of historical introgression. However, this limited view of the genome cannot resolve the timing and genomic consequences of divergence between *Mimulus* species, nor can it inform the extent or consequences of introgression between them.

We present the first population genomic analysis of *M. guttatus* and *M. nasutus*, spanning diverse ecotypes collected from throughout the species' ranges. We use these dense and contiguous population genomic data to estimate the population-split time, quantify rapid loss of ancestral variation accompanying the transition to selfing in *M. nasutus*, and identify ongoing, bidirectional introgression. Additionally, we observe a negative correlation between the recombination rate and interspecific divergence between *M. nasutus* and sympatric *M. guttatus*, a result best explained by selection against introgressed *M. nasutus* ancestry in *M. guttatus*. Our approach provides a detailed view of differentiation and introgression in a tractable ecological, genetic, and evolutionary model system.

## Results

**Figure 1:**
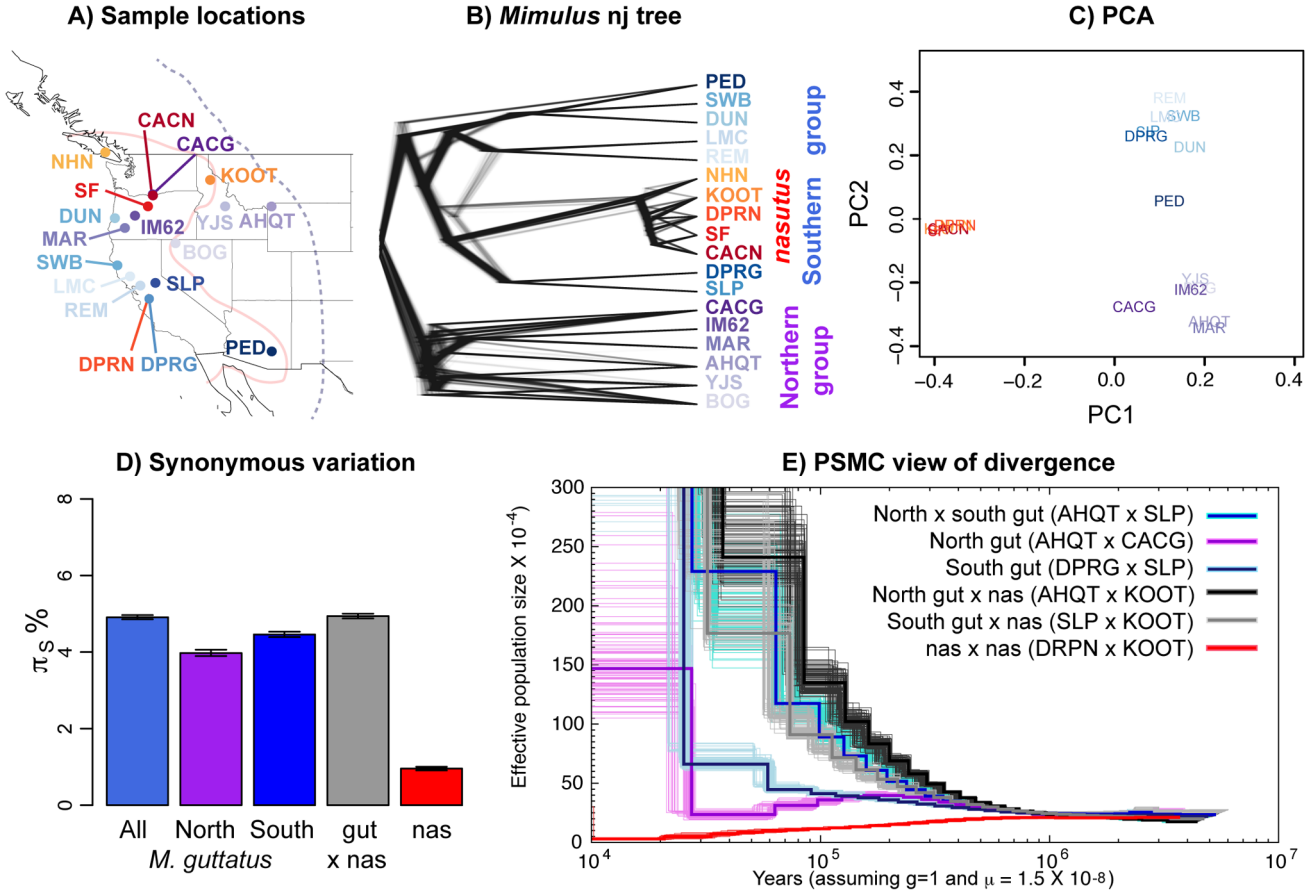
Relationships among *Mimulus* samples. ***A***) A map of all samples with identity and species denoted by population code and color, respectively. *M. guttatus* individuals are colored in blues and purples, and *M. nasutus* is colored in oranges and reds. Approximate species distribution boundaries are shown for *M. guttatus* with the purple dashed line and for *M. nasutus* with the solid pink line. Sympatric samples are marked by lines originating in the same location. ***B***) A neighbor-joining tree for all samples rooted by *M. dentilobous*. Sample and species colors are identical to those in 1A. The consensus tree was constructed from the pairwise distance matrix described in the main text with the *nj* function in the R package, *ape*, and smoothed with the function, *chronopl*, with λ = 1, an implementation of Sanderson's nonparametric rate smoothing program, r8s [102]. The distribution of trees is plotted using *densitree* [103] as implemented in the *R* package, *phangorn* [103]. Each of the one thousand trees is a resampling of the 14,000 SNPs with replacement. All of the 14,000 trees support a strong split between northern and southern groups. The one exception is LMC, a California sample that clusters with southern and northern *M. guttatus* in 542 and 458 of the 1,000 bootstrapped trees, respectively. ***C***) A principle component analysis of these data, excluding *M. dentilobus*. ***D***) The mean number of pairwise sequence differences per fourfold degenerate site (π_S_) within and among *Mimulus* species and populations, including uncertainty via a block bootstrap. ***E***) Demographic history as inferred by the PSMC. Inferred population size through time is shown for pairwise combinations of haploid genomes of *M. guttatus* and/or *M. nasutus* individuals. Black/gray = interspecific comparisons with allopatric *M. guttatus*. Blue and violet = intraspecific *M. guttatus* comparisons. Red = intraspecific *M. nasutus*. For each pair-wise comparison, the thick dark line represents the point inference and each lighter-colored, thin line represents 1 of 100 bootstraps (see Text S1).

We present and analyze a population genomic dataset of nineteen lab and/or naturally inbred (see Table S1) *Mimulus* samples – thirteen *M. guttatus*, five *M. nasutus*, and one of *M. dentilobus*, an outgroup. We generated sequence data for five of these samples (four *M. nasutus* and one *M. guttatus*), and accessed data for the other 14 samples from previously existing resources (see Materials and Methods for sequence sources and processing details). Collections spanned the ecological and geographic ranges of each species (Figure 1A and Table S1). Many of our analyses focus on four *M. guttatus* and four *M. nasutus* collections sequenced to relatively high depth (13.8×–24.7×) and with identical read lengths (100 bp, paired-end reads). Pairwise comparisons of nucleotide diversity among all nineteen samples are presented in Tables S2A and S3. Individual heterozygosity in the eight focal samples was relatively low (see METHODS) and was not clustered in regions of residual heterozygosity (contra the observation in naturally inbred *Capsella rubella* [20]) suggesting that natural and lab inbreeding has resulted in near total homozygosity by (recent) descent. Throughout this manuscript, we present results from samples aligned with bwa [32]. In Table S2B, we show that qualitative patterns of relative differentiation in focal samples are consistent when analyzed with a different alignment program, Stampy [33], demonstrating that our results are robust to the choice of bioinformatic pipeline.

Of our focal *M. guttatus* samples, CACG and DPRG are narrowly sympatric with *M. nasutus* populations from the northern and southern portion of both species' ranges, respectively. Our focal northern allopatric *M. guttatus* collection, AHQT, is well outside the geographic range of *M. nasutus*. By contrast, the southern allopatric collection (SLP) is geographically close to *M. nasutus* populations. Our focal *M. nasutus* collections also include sympatric and allopatric samples from the north and south (Table S1).

### Speciation history

Overall patterns of genomic differentiation show deep population structure in *M. guttatus*, with *M. nasutus* diverging from a central Californian *M. guttatus* population approximately 200 kya.

To visualize pairwise relatedness, we constructed a rate-smoothed neighbor-joining (nj) tree (see METHODS for a discussion of the nj approach in population genetics). This tree clearly displays a deep phylogeographic split within *M. guttatus*, roughly corresponding to northern and southern parts of its range; however, geography is an imperfect predictor of genetic structure within *M. guttatus* (*e.g.*, DUN is from a northern latitude yet clusters with our southern *M. guttatus* samples). The tree places all *M. nasutus* samples as a node within the southern *M. guttatus* cluster (Figure 1B). The fact that *M. guttatus* is paraphyletic suggests that *M. nasutus* budded from within a structured ancestral *M. guttatus* population. A principle component analysis (PCA, Figure 1C) also reveals the genetic structure within *M. guttatus* – PC2 differentiates northern and southern *M. guttatus* groups. Consistent with the single origin of *M. nasutus*, PC1 separates *M. guttatus* from the strongly clustered *M. nasutus*, presumably as a consequence of a shared history of genetic drift among these *M. nasutus* samples. Down-sampling to any one *M. nasutus* sample controls for this shared drift, and places *M. nasutus* within southern *M. guttatus* (Figure S1).

To support these qualitative inferences we generated a quantitative description of genetic structure within *M. guttatus*, focusing on our high-coverage (focal) samples. Pairwise sequence diversity at synonymous sites within northern (π_S AHQT×CACG_ = 3.97% [3.89%–4.06%]) and southern (π_S DPR×SLP_ = 4.45% [4.39%–4.52%]) *M. guttatus* samples is significantly lower than that within *M. guttatus* overall (π_S_ = 4.91% [4.85%–4.96%]), and between the north and south (π_S_ = 5.26% [5.20%–5.30%], Figure 1D). Diversity within the northern and southern clades is consistent with a very large effective population size (*N_e_*) of approximately one and a half million chromosomes for both groups (assuming the per generation per base mutation rate, μ = 1.5×10^−8^ [following Koch *et al.* 2001]). As a simple estimate of the population split time (τ generations), ignoring possible introgression, we assume that the divergence between populations is the sum of pairwise diversity (π) within an ancestral population and the product of the per-generation mutation rate, μ, and two times the split time [34]. Using this relationship, and representing ancestral diversity by the southern *M. guttatus* samples, we set τ = (π_S NorthGut×SouthGut_−π_S SouthGut_)/2μ and estimate a split between northern and southern *Mimulus* populations more than a quarter of a million years ago (265 ky [251 ky–280 ky], assuming an annual life history). As above, this estimate assumes μ = 1.5×10^−8^/bp/generation but can be linearly rescaled by alternative estimates of μ. For example, readers can multiply divergence time estimates by a factor of two if they prefer the estimate of μ = 7×10^−9^/bp/generation [35].

Interspecific divergence between *M. guttatus* and *M. nasutus* (*d_S_* = 4.94% [4.88%–5.00%]) is comparable to overall *M. guttatus* diversity, and exceeds diversity within northern or southern *M. guttatus* collections (Figure 1D). We derive a simple estimate of split time between *M. guttatus* and *M. nasutus* as we did above to estimate the split between focal northern and southern *M. guttatus* samples. Using the difference between divergence of *M. nasutus* from the southern, allopatric *M. guttatus* sample (to minimize the influence of recent introgression and historical divergence between *M. guttatus*’ genetic clusters) and a proxy for diversity in an ancestral population (southern *M. guttatus*), we estimate that *M. nasutus* and *M. guttatus* split approximately 200 ky ago, τ = (π_S Nas×AlloSouthGut_−π_S SouthGut_)/2μ = 0.5875%/2μ = 196 ky [181 ky–212 ky].

As a complementary inference of historical patterns of divergence within *M. guttatus* and between species, we applied Li and Durbin's implementation of the pairwise sequentially Markovian coalescent (PSMC) [36] to pairwise combinations of focal haploid genomes (Figures 1E and S2, S3, S4, S5, S6). The PSMC analysis infers large population sizes within both northern (CACG×AHQT) and southern (SLP×DPRG) samples, with an apparent bout of strong recent population growth. However, as we have sampled from a structured population the inferred larger recent population sizes likely represent reduced coalescent rates caused by population structure, rather than dramatic recent increases in *N_e_*. Likewise we infer a very low rate of coalescence (a very large effective population size) in the recent past between northern and southern *M. guttatus* (SLP×AHQT) compared to within these groups (SLP×DPRG and CACG×AHQT, Figures 1E and S2) likely reflecting the strong genetic structure within range-wide *M. guttatus*.

We also use this PSMC analysis for an additional estimate of the approximate split time, by assessing when the inferred coalescent rate between species decreases (*i.e.*, the population size estimate increases) relative to the rate within *M. guttatus* [see 36]. In doing so, we focus on the southern *M. guttatus* samples that fall closest to *M. nasutus* in our nj tree. The inferred coalescent rate between *M. nasutus* and southern *M. guttatus* (SLP×KOOT, gray line) decreases relative to the rate within southern *M. guttatus* (SLP×DPRG, dark blue/navy line), *i.e.*, the lines diverge, from ∼500 to ∼300 kya, suggesting either a gradual split between species over that time span, or a hard split sometime within that range (Figures 1E and S3). This result, which represents an upper bound on time since speciation, is qualitatively similar to our lower estimate based on synonymous nucleotide variation among these samples. We note that for both analyses, historical introgression of *M. nasutus* into SLP would make this split seem more recent than it actually was.

### Genomic consequences of the transition to selfing

Patterns of genomic variation within *M. nasutus* reflect the genomic consequences of a recent transition to selfing. Synonymous diversity within *M. nasutus* (π_S_ = 1.09% [1.03%–1.14%], Figure 1D) is one fifth that observed within *M. guttatus*, consistent with a high rate of genetic drift since *M. nasutus*’ origin. Moreover, most ancestral variation in *M. nasutus* has been homogenized: of the fixed differences between *M. nasutus* and *M. guttatus*, 90% are derived in *M. nasutus* and 10% are derived in *M. guttatus* (when polarizing by *M. dentilobus*). Although *M. nasutus* has lost much of its ancestral variation, shared variants still constitute a much higher proportion of its polymorphism (50%) relative to an equally sized sample of *M. guttatus* (10%). This pattern reflects both the paraphyly of *M. guttatus* and the incomplete sorting of variation present in *M. nasutus*’ founders.

Consistent with this reduction in nucleotide diversity and incomplete sorting of ancestral variation, PSMC analyses infer a dramatic decline in *M. nasutus*’ effective population size after it split from *M. guttatus* (compare red and black-gray lines in Figure 1E, see also Figure S4), suggesting that the evolution of selfing roughly coincided with *M. nasutus*’ split from *M. guttatus*. We caution, however that interpretation of PSMC's estimated population size in *M. nasutus* is not straightforward. This is because the transition to selfing reduces the population recombination rate more than the population mutation rate [14]; however, Li and Durbin's [36] implementation of the PSMC assumes that both these values change proportionally with the historical effective population size.

Relative to expectations under selective neutrality and demographic equilibrium, *M. nasutus* contains an excess of high-frequency derived synonymous alleles (Figure S7). We interpret this observation as a reflection of a recent population contraction. This interpretation is in agreement with the decreased synonymous diversity in *M. nasutus* relative to *M. guttatus* and our PSMC-based inference of a reduction in *N_e_*. However, population structure within *M. nasutus* may also contribute to this excess of high frequency derived alleles [37], [38]. By contrast, in *M. guttatus* we observe slightly more rare synonymous alleles than expected under a neutral equilibrium model, reflecting recent growth, population structure, and/or weak selection against unpreferred codons.

**Figure 2:**
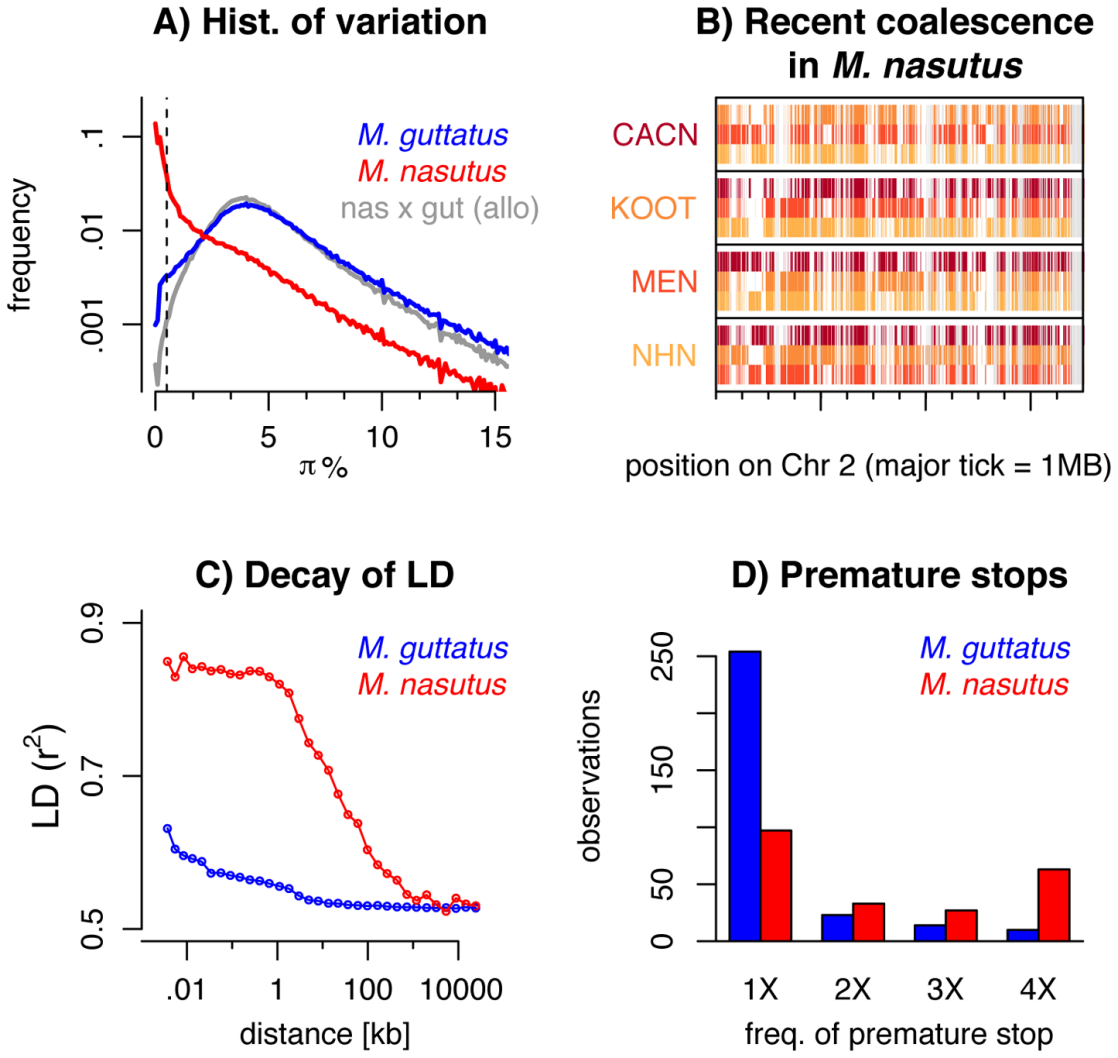
Genomic consequences of the transition to selfing. ***A***) A histogram of pairwise sequence diversity (π) within and between species in overlapping 5 kb windows. For interspecific comparisons we focus only on allopatric *M. guttatus* populations. The dotted line denotes π_S_<0.5% or 170 ky of divergence. ***B***) Moving along a part of chromosome two, for all *M. nasutus* samples, we color genomic regions in which the focal individual (y-label) and another *M. nasutus* sample, indicated by color, recently coalesce (π_S_≤0.5%). White regions coalesce more distantly in the past (π_S_>0.5%) and gray regions indicate insufficient density of informative sites. Major tick-marks on the x-axis indicate 1 megabase. ***C***) Linkage disequilibrium (measured as *r^2^*) within *M. nasutus* (red) and *M. guttatus* (blue), as a function of physical distance. ***D***) The number of premature stop codons observed in one, two, three, or four *M. nasutus* (red) and *M. guttatus* (blue) samples.

The distribution of synonymous diversity across the genome (overlapping 5 kb windows with a 1 kb slide, Figure 2A) bolsters the view that *M. nasutus*’ genomic diversity is a mixture of closely related genomic regions that rapidly coalesce in the small *M. nasutus* population, and distantly related regions that do not coalesce until joining a large *M. guttatus*-like ancestral population. In pairwise comparisons of sequence diversity within *M. nasutus*, half of the genomic windows are differentiated by π_S_<0.5% (corresponding to ∼170 thousand years of divergence), reflecting recent common ancestry since the species split. On the other hand, one third of such windows are differentiated by π_S_>2.0%, reflecting deep ancestry in a large ancestral population (Figure 2A, see Figure S8 for different window sizes).

These findings contrast sharply with comparisons within *M. guttatus*, as well as between *M. nasutus* and allopatric *M. guttatus* samples, for which recent common ancestry since the species split is rare (π_S_<0.5% for less than 1.5% of 5 kb windows) and deep coalescence is the norm (mode π_S_ = 4%, Figure 2A, a result roughly consistent across window sizes, Figure S8). Under the neutral coalescent, a pair of lineages will fail to find a common ancestor with each other by generation t with probability *e* ^−t/Ne*^, where N_e__*_ is the (constant) effective number of chromosomes. Therefore, the observation that half of our *M. nasutus* windows share a common ancestor in the past ∼170 ky, by an admittedly crude calculation, predicts a population size between 150k and 250k effective chromosomes (compared to the estimated N_e__*_ of 1.5 million in *M. guttatus* from synonymous diversity, above). This ten-fold reduction in effective population size as compared to *M. guttatus* far exceeds both the two-fold decrease in *N_e_* expected to accompany the evolution of selfing and the four-fold decrease calculated by the difference in intraspecific variation.

Across the genome, the mosaic nature of ancestry within *M. nasutus* is apparent as long contiguous regions of recent common ancestry (colored windows in Figures 2B and S9) interrupted by regions of deep ancestry, due to incomplete lineage sorting and/or historical introgression (white windows in Figure 2B and S9). This block-like ancestry structure results in extensive linkage disequilibrium (LD) in *M. nasutus*. In contrast to *M. guttatus*, for which the sample pairwise LD drops halfway towards its minimum values within only 15–20 base-pairs, LD in *M. nasutus* decays much more slowly, not dropping halfway towards its minimum values until 22 kb (Figure 2C). This represents a thousand-fold difference in the decay of LD, as compared to a more modest ten-fold reduction in the effective population size between *M. nasutus* and *M. guttatus*. This dramatic difference in the scale of LD between *Mimulus* species is likely due to a major reduction in the effective recombination rate within the selfing *M. nasutus*. We use this difference to derive a simple estimate of *M. nasutus*’ selfing rate using Eq. 1 of Nordborg [14]. Nordborg showed that the ratio of the population-scaled recombination to mutation rate in selfers is reduced by a factor of 1-*F* compared to the same population if it was outcrossing, where F is the inbreeding coefficient. Substituting in the hundred fold difference in the ratios of effective population size and decay of LD between *M. nasutus* and *M. guttatus*, we arrive at 1−*F* = 0.01. Assuming a constant selfing rate s, *F* = *s*/(2−*s*), *M. nasutus*’ selfing rate is approximately 99%.

Patterns of sequence variation suggest a reduced efficacy of purifying selection in *M. nasutus*, a result consistent with extensive genetic drift and/or linked selection within *M. nasutus*. All *M. nasutus* samples contain more premature stop codons than any *M. guttatus* sample (*M. nasutus*: mean 124.0, range = 121–126, *M. guttatus*: mean 95.5, range = 86–102), and a large proportion of these premature stops are at high frequency in *M. nasutus* (Figure 2D). For 27 of the 29 fixed differences for a premature stop codon, *M. nasutus* carries the premature stop and *M. guttatus* carries the intact allele. We acknowledge that errors in annotation could underlie some of the excess of premature stop codons inferred in *M. nasutus*. However, *M. nasutus* and the focal southern *M. guttatus* samples are equally diverged from the reference and yet our southern *M. guttatus* samples do not show this excess. As such, annotation error is likely not a strong contributor to the large and consistent interspecific differences in premature stop codons observed.

Additionally, after standardizing by synonymous variation, we observe an excess of putatively deleterious, non-synonymous variation in *M. nasutus* relative to *M. guttatus* (π_N_/π_S_ = 0.197 [0.192–0.203] and 0.157 [0.155–0.160], respectively). However, this difference is not yet reflected in divergence between the species (*d_N_/d_S_* = 0.156 [0.154–0.159]), presumably because interspecific sequence differences largely reflect variation that predates the origin of selfing in *M. nasutus* rather than the relatively few mutations accrued within the past ∼170 ky. This pattern of elevatedπ_N_/π_S_ in selfing species but only modest d_N_/d_S_ between selfers and their close relatives is common [15], even in genome-wide analyses (*e.g.*, [20]). We note that elevated π_N_/π_S_ in selfing species may reflect the faster rate at which nonsynonymous diversity approaches equilibrium after a reduction in diversity compared to synonymous mutations [39], [40], rather than a consequence of a reduced efficacy of purifying selection. However, this interpretation cannot explain the absolute excess of premature stop codons in *M. nasutus*.

### Ongoing gene flow and its consequences

#### Inferring the history of introgression from *M. nasutus* into *M. guttatus*

**Figure 3:**
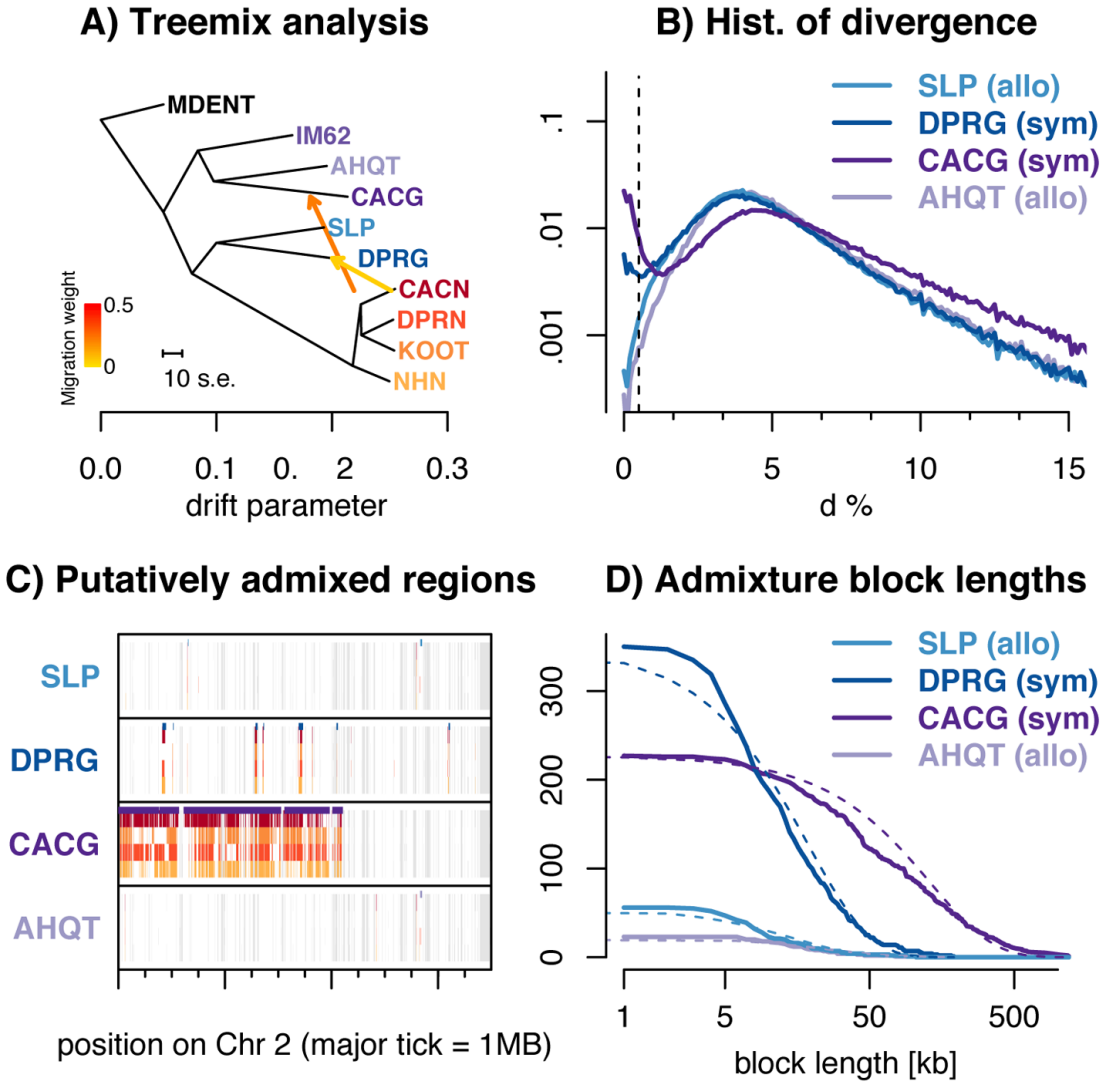
Introgression of *M. nasutus* material into *M. guttatus*. ***A***) Treemix suggests introgression from *M. nasutus* in to sympatric *M. guttatus* samples. ***B***) A histogram of interspecific pairwise sequence divergence in 5 kb windows for each *M. guttatus* sample. ***C***) Introgression across a chromosome - Moving along a part of chromosome two for all *M. guttatus* samples, we color genomic regions in which the focal individual (y-label) and a *M. nasutus* sample, indicated by color, recently coalesce (π_S_≤0.5%). White regions coalesce more distantly in the past (π_S_>0.5%) and gray regions indicate insufficient density of informative sites. Major tick marks on the x-axis indicate 1 megabase. Purple bars above each focal individual denote greater than a 95% posterior probability of *M. nasutus* ancestry as inferred from our HMM. ***D***) Admixture block length distribution - The number of admixed blocks, as inferred by a greater than 95% posterior probability of *M. nasutus* ancestry from our HMM, longer than x. The expected exponential distribution is marked with a dotted line.

A surprising result from our analyses of genomic divergence is that although the sympatric northern *M. guttatus* sample (CACG) is far from *M. nasutus* in both PCA space and position in the neighbor joining tree (Figures 1B and 1C, respectively), CACG is least diverged from *M. nasutus* according to the expected number of synonymous pairwise differences (see Table S2A, a result that holds across alignment pipelines, Table S2B). To test whether these seemingly contradictory observations are due to introgression, we used Treemix to construct a population tree featuring admixture events [41]. Treemix finds a clear signal of admixture from *M. nasutus* into the population ancestral to CACG (Figure 3A). This result holds across a range of different sample subsets (Figure S10). Some Treemix analyses also suggest introgression from *M. nasutus* into the population ancestral to our southern, sympatric *M. guttatus* (DPRG); however, the manifestation of this second signal varies across sample subsets (Figure S10, see Text S1). A D-test of introgression based on 4 populations [42] provides additional evidence for historical introgression of *M. nasutus* ancestry into both sympatric *M. guttatus* samples. Specifically we find that D(CACG, AHQT; *M. nasutus*, SLP) and D(DPRH, SLP; *M. nasutus*, AHQT) are both significantly greater than zero (D estimates equal 0.408 and 0.068, and standard errors of 0.074 and 0.013, respectively, see *METHODS*), further supporting *M. nasutus* introgression into CACG and DPRG.

The bimodal distribution of divergence between *M. nasutus* and CACG in overlapping 5 kb genomic windows (dark purple lines in Figures 3B, see other window sizes in S11) provides further evidence for introgression. For most windows, CACG shows deep divergence with *M. nasutus* (half of windows have more than 5% sequence divergence), but approximately one tenth of windows show nearly no divergence (π<0.5%) and were likely recently introduced by introgression. We find a similar, but more subtle, bimodal distribution of divergence between the sympatric, southern *M. guttatus* sample, DPRG, and *M. nasutus* (2.5% of 5 kb windows in this comparison exhibit less than 0.5% sequence divergence – dark blue lines in Figure 3B). This signal is apparent across a range of window sizes, with increasing window size dampening this effect as short admixture blocks are averaged into the genomic background (see Figure S11). We do not observe this binomial distribution of divergence times between *M. nasutus* and allopatric *M. guttatus* – less than 0.5% of genomic windows in SLP and AHQT are less than 0.5% diverged from *M. nasutus* (Figures 2A, 3B, S8, and S11). The PSMC analysis also reflects ongoing introgression in sympatry, as it highlights recent common ancestry between sympatric *M. guttatus* samples (CACG and DPRG) and *M. nasutus* (Figures S5 and S6). Moreover, genomic regions of low interspecific divergence are spatially clustered (Figures 3C and S9), consistent with recent and ongoing introgression that is slowly broken down over generations of recombination. We point readers towards the recent work of Wilkinson-Herbots [43] for parameter inference from distributions like those in Figures 2B and 3B.

We utilize the spatial distribution of windows of low interspecific divergence to probabilistically infer regions of recent *M. nasutus* or *M. guttatus* ancestry across the genomes of focal *M. guttatus* samples (Figures 3C and S9) using a Hidden Markov Model (‘HMM’ see METHODS). We estimate *M. nasutus* ancestry proportions of 15.1% and 5.7% in our sympatric northern (CACG) and southern (DPRG) *M. guttatus* samples, respectively, using output from the HMM. We find much lower proportions of *M. nasutus* ancestry in the allopatric *M. guttatus* genomes samples, 1.1% in the south (SLP) and 0.6% in the north (AHQT). This low level of admixture inferred in allopatric samples may reflect low levels of gene flow into allopatric *M. guttatus* and/or misassigned regions of incomplete lineage sorting.

To learn about the timing of admixture, we find contiguous regions of individual *M. guttatus* genomes with a greater than 95% posterior probability of *M. nasutus* ancestry and display the length distribution of these blocks in Figure 3D. The block length distribution can be used to disentangle complex histories of recent introgression [44], [45]. The mean admixture block lengths are 132 kb (∼0.74 cM, n = 227 blocks) and 18.6 kb (∼0.10 cM, n = 350 blocks) for northern (CACG) and southern (DPRG) sympatric *M. guttatus* samples, respectively. Because our HMM occasionally breaks up what seem to be contiguous blocks of admixed ancestry, we instituted strategies to fuse admixture blocks, none of which greatly influenced our block length distributions (see Text S1 and Figure S12). Under a single temporal pulse of gene flow, admixture block lengths are exponentially distributed with a mean length (in Morgans) that is the reciprocal of the number of generations since admixture. With this model, we estimate 135 and 962 generations since admixture for CACG and DPRG samples, respectively (Table S4). Because these estimates are much more recent than the split time of ∼200 kya, this pattern cannot be explained by incomplete lineage sorting.

Although this pulse model provides an intuitive summary of time back until an admixed region in *M. guttatus* coalesces with *M. nasutus*, our data are inconsistent with a model of a single admixture pulse. In comparison to the exponential decay expected from a single admixture pulse, there are fewer blocks of *M. nasutus* ancestry of intermediate length in admixed *M. guttatus* genomes (Figure 3D and Table S4). Thus, these estimates should be viewed as average times back to an admixture event, rather than an estimated date of one admixture event. Furthermore, many of these blocks come from far enough back in the past that they are likely derived from a very large number of historical introgression events (Table S4, see also Methods and Text S1 for quantitative support for these points).

#### Detecting introgression from *M. guttatus* into the selfer *M. nasutus*

We also detected a signal of introgression from *M. guttatus* into *M. nasutus*. Identifying this signal is difficult, compared to identifying introgression into *M. guttatus*, due to the short scale of LD in *M. guttatus* and the similarity of interspecific divergence and *M. guttatus* diversity. Both the incomplete sorting of ancestral variation and recent introgression from *M. guttatus* can cause one *M. nasutus* sample to differ from all others in some genomic regions. However, introgressed regions differ from incompletely sorted variation, in that admixed regions should *(1)* persist for a long physical distance and *(2)* more closely resemble the putative source of admixture – a geographically close *M. guttatus* – than other *M. nasutus* samples. To test whether introgression from *M. guttatus* contributes to variation in *M. nasutus*, we focus on long genomic regions (>20 kb) where one “outlier” *M. nasutus* sample differs from all other *M. nasutus* samples (as inferred by pairwise π_S_>1%, alternative thresholds explored in Text S1). We then ask whether these outlier sequences are less diverged from allopatric but geographically close *M. guttatus* samples than the non-outliers. For example, are “outlier” genomic regions in a northern *M. nasutus* sample less diverged from allopatric northern *M. guttatus* than are the three non-outlier *M. nasutus* samples in these regions? By excluding sympatric *M. guttatus* samples, we avoid conflating introgression into *M. nasutus* with the extensive signature of introgression from *M. nasutus* into *M. guttatus*.

Pooling across northern *M. nasutus* samples (NHN, KOOT, CACN), outlier windows are more often genetically closer to the northern *M. guttatus* sample (AHQT) than are the non-outlier windows (272 of 490, one sided binomial test against the null expectation of 50% P = 0.008. Table S5 and Text S1 show that this result is robust to designation of outlier windows). However, this result is individually significant only for our most geographically remote *M. nasutus* sample, NHN (141 of NHN's 249 outlier windows are closer to northern *M. guttatus* than are the non-outlier *M. nasutus*). Additionally, the southern *M. nasutus* sample, DPRN, contained the smallest proportion of outlier windows resembling northern *M. guttatus*. By contrast, no samples have a disproportionate share of outliers genetically close to southern *M. guttatus*, SLP (Table S5), consistent with little or no introgression into DPRN (note, however, that this comparison is likely underpowered because of the genetic proximity of southern *M. guttatus* to the population that founded *M. nasutus*).

#### Divergence between *M. nasutus* and sympatric *M. guttatus* samples increases with decreasing local recombination rate

We observed a genome-wide negative relationship between absolute synonymous divergence from *M. nasutus* (*i.e.*, the mean number of pairwise sequence differences at synonymous sites) and the local recombination rate in both sympatric *M. guttatus* samples. (DPRG×*M. nasutus*, Spearman's ρ = −0.080, P = 0.0008; CACG×*M. nasutus*, Spearman's ρ = −0.0718, P = 0.0027). These sympatric *M. guttatus* samples display approximately a 5% increase in synonymous nucleotide divergence to *M. nasutus* in genomic regions with below average recombination rates. This signal is substantially weaker in the allopatric southern *M. guttatus* sample (SLP×*M. nasutus*: Spearman's ρ = −0.0521, P = 0.0297), which is nested within the range of *M. nasutus*, and seems to be absent in comparisons with the allopatric northern *M. guttatus* sample, which occurs well outside of *M. nasutus*’ range (AHQT×Nas: Spearman's ρ = −0.0261, P = 0.2768). This result holds after accounting for the potential confounding effects of sequencing depth and divergence from the *M. dentilobus*, a proxy for mutation rate variation (see Methods, Text S1 and Table S6).

This negative relationship between divergence and the recombination rate in sympatry is consistent with selection against *M. nasutus* ancestry reducing effective gene flow at linked sites (see [46], for related theory and tentative evidence to date), and inconsistent with alternative scenarios. Specifically, neutral processes cannot generate this correlation because mean neutral coalescent times are independent of recombination rates [47]. Additionally, although selective sweeps and background selection can influence relative measures of divergence, such as *F_ST_* [46], [48], [49], these models of linked selection do not influence the expected substitution rates at neutral sites [50].

We observe no relationship between diversity and the recombination rate within *M. guttatus* (Spearman's ρ = −0.0275, P = 0.244) or *M. nasutus* (Spearman's ρ = 0.0291, P = 0.218). Therefore, it seems unlikely that linked selection processes within species (background selection and selective sweeps) could explain this result. Note that this lack of signal within populations is interesting in its own right, as it suggests that linked selection may not strongly influence species-wide diversity in *M. guttatus*. Furthermore a lack of a correlation within *M. nasutus* suggests that we do not have any clear evidence of linked selection being the cause of the rapid loss of diversity in *M. nasutus*.

## Discussion

### Speciation history and the origin of *M. nasutus*

Genetically, *M. nasutus* clusters with central Californian *M. guttatus* samples, suggesting that speciation post-dated the differentiation of some *M. guttatus* populations. Thus, speciation in this pair is best described as a ‘budding’ of *M. nasutus* from *M. guttatus*, rather than a split of an ancestral species into two. We observe an approximate coincidence between the timing of divergence and the decline in population size in *M. nasutus* (as inferred from our PSMC analysis). This observation could be a result of the transition to selfing being linked to speciation (see [11] for phylogenetic evidence of this link in the Solanaceae and [51], [52] for a likely case in *Capsella*). However, given the misspecification of the PSMC model for the transition to selfing (see above), this observation should be treated with caution. More work is needed to develop methods to test whether split times and changes in selfing rate occur concurrently to see if this is indeed a general pattern.

Future genomic analyses across the *M. guttatus* complex and other species groups will facilitate an in-depth view of the causes and consequences of speciation by the budding of selfing and/or endemic populations from widespread parental species. We note that recent phylogenetic analyses of species' ranges suggest that this mode of speciation is common in *Mimulus* [53] and other flowering plants [54].

We estimate that *M. nasutus* split from a *M. guttatus* population within the last two hundred to five hundred thousand years (with our estimate of ∼200 ky, inferred from differences in synonymous sequence differences within and between species, and the estimate of ∼500 ky corresponding to conservative estimates of population splits from the PSMC). This lies between the ∼50 ky separating selfing *Capsella rubella* from outcrossing *C. grandiflora* [20], [55] and *Arabidopsis thaliana* which has potentially been selfing for over a million years ([56], having split from *A. lyrata* ∼3–9 Mya [57]). Although 200 ky represents a relatively short time evolutionarily, it implies that *M. nasutus* managed to survive numerous dramatic bioclimatic fluctuations.

### The transition to selfing and its genomic consequences

The transition from outcrossing to self-fertilization in *M. nasutus* has had clear consequences on patterns of genomic variation. In *M. nasutus*, linkage disequilibrium exceeds that in *M. guttatus* by three orders of magnitude. This result suggests a high selfing rate in *M. nasutus* (estimated above at 99%), consistent with direct estimates from field studies [24]. We observe a four-fold drop in diversity and infer a ten-fold reduction in the recent effective population size in *M. nasutus* compared to *M. guttatus*, values far exceeding the two-fold decrease in *N_e_* expected as a direct consequence of selfing [58], [59]. This more than two-fold reduction in *N_e_* of selfing populations relative to their outcrossing relatives has been identified in other plant [17], [55] and animal [60]–[62] species pairs, and may be partially due to extreme founding bottlenecks, frequent colonization events and/or demographic stochasticity that further increase the rate of genetic drift [13], as well as a heightened influence of linked selection in selfing taxa [60], [63]–[65].

Selfing populations are expected to experience a reduced efficacy of purifying selection accompanying the drop in effective population size and recombination rates [15], [65], [66]. Consistent with these predictions, *M. nasutus* has accumulated numerous putatively deleterious mutations, including nonsynonymous variants and premature stop codons. Presumably, this elevation in radical genetic variants reflects a reduction in the efficacy of purifying selection due to a high rate of genetic drift and linked selection, as well as perhaps the escape of some genes (*e.g.*, loci involved in pollinator attraction) from the selective constraints they faced in an outcrossing population (*e.g.*, [17]).

### Selfing as a reproductive barrier and its significance for ongoing gene flow

Despite multiple reproductive isolating barriers, including mating system differences, we find ongoing, bidirectional introgression between *M. guttatus* and *M. nasutus*.

Evidence of ongoing introgression from the selfer, *M. nasutus*, into the outcrosser, *M. guttatus*, is particularly stark. There are numerous evolutionary implications of introgression from selfers to outcrossers. Introgression of deleterious mutations accumulated in selfers may introduce a genetic load to outcrossers. This burden would result in selection against genetic material from selfers in hybridizing outcrossing populations, and could ultimately favor reinforcement of reproductive isolation. Alternatively, such introgression could provide a multi-locus suite of variation facilitating self-fertilization, and other correlated traits (*e.g.*, drought resistance and rapid development), in favorable environments, as appears to be the case in introgression between wild and domestic beets (*Beta vulgaris*, [67]).

Evidence of introgression from *M. guttatus* into *M. nasutus* is subtler, but is potentially critically important. Even relatively low levels of introgression into a selfer may rescue the population from a build up of deleterious alleles, and reintroduce adaptive variation, and so may lower its chances of extinction, a fate considered likely for most selfing lineages [68], [69]. However, before potentially rescuing a selfing population from extinction, genomic regions introduced from outcrossing species must themselves survive a purging of deleterious recessive alleles.

Higher rates of introgression from *M. nasutus* to *M. guttatus* would be consistent with the prediction that backcrosses should be asymmetric – because bees preferentially visit plants with larger flowers [70], [71] and/or larger floral displays [72], [73], both features of *M. guttatus*, visits to *M. nasutus* and F_1_ hybrids are likely preceded and followed by visits to *M. guttatus* [24], [30]. Consistent with this prediction, direct estimates of hybridization in the DPR sympatric population reveal that F_1_ hybrids are the product of *M. nasutus* maternal and *M. guttatus* paternal parents, respectively [24]. However, we caution that it is considerably more challenging to identify introgression into *M. nasutus* than into *M. guttatus*, as the similarity between interspecific divergence and diversity in *M. guttatus* makes historical admixture difficult to separate from the incomplete sorting of *M. nasutus*’ ancestral variation. We further note that, although asymmetrical introgression from selfers to outcrossers has been detected in other systems (*Pitcairnia* [74], and potentially in *Geum* [75], [76]), the relative contribution of selfing vs. other isolating barriers and/or selection is unclear. Dense sampling of sympatric and allopatric populations of outcrossing species experiencing ongoing gene flow with selfing relatives will allow for tests of these hypotheses. Importantly, the number, location and length-distribution of admixture blocks identified from genomic analyses provide information about the longer-term consequences and pace of introgression between selfers and outcrossers.

### Selection against hybrids and implications for species maintenance

The numerous short blocks (in addition to long blocks) of *M. nasutus* ancestry observed in *M. guttatus* suggest that *M. nasutus* ancestry can potentially persist in an *M. guttatus* background for many generations. Despite this, *M. guttatus* and *M. nasutus* are still ecologically and genetically distinct.

We identified a genome-wide signature of selection against introgression of *M. nasutus* ancestry in *M. guttatus*, in the form of a negative relationship between the local recombination rate and absolute divergence. This relationship was highly significant in both sympatric comparisons, but only weakly significant in parapatry, and insignificant in allopatry. Additionally, we did not find a relationship between recombination and diversity within either species. Moreover, unlike a negative relationship between the recombination rate and relative measures of differentiation, such as *F_ST_* or the number of fixed differences [*e.g.*], [ 77], [78,79], this finding cannot also be explained by a high rate of hitchhiking or background selection within populations since the species split [46], [48], [49]. Instead, it seems more consistent with *M. nasutus* ancestry being selected against more strongly in regions of low recombination due to linkage with maladaptive alleles that introgression would introduce. This suggests that the genome has potentially congealed as a barrier to gene flow in low recombination regions. We note that this ‘congealing’ (*sensu* Barton [80], [81]) requires a threshold density of locally adaptive mutations, measured in recombination distance, and does not require a complex model of multi-locus coadaptation. Previous reports of absolute divergence near the breakpoints of inversions (*e.g.*, [82], [83]), or in centromeres relative to telomeres [84], suggested this result; however, genome-wide evidence for this basic prediction is scarce.

Further work, including experiments measuring selection on genetic variants in the wild, and larger sample sizes from both allopatric and sympatric populations, is needed to pinpoint which (if any) genomic regions are particularly strongly selected against in hybrids. Genetically mapped loci for adaptive interspecific differences [85] and hybrid inviability and sterility [29] are promising candidates. Indeed, recent analyses of the distribution of Neanderthal haplotype blocks in ∼1000 human genomes has identified genomic candidates for adaptive introgression from Neanderthals to humans and an apparent paucity of introgression at loci putatively influencing male fertility [86].

### Summary and future prospects

Our analyses of whole genomes from the *M. nasutus* - *M. guttatus* species pair provide a broad view of both the historical divergence in this group and the ongoing processes by which they remain distinct. Less than a half million years ago, a semi-isolated *M. guttatus* population evolved self-pollination and ultimately transformed into modern day *M. nasutus*. In the intervening time, this population experienced a contraction in effective population size, and accumulated deleterious mutations while spreading geographically across western North America. More broadly, our work demonstrates that much can be learned about population history from resequencing relatively few samples in a group with an annotated genome and an integrated physical-genetic map.

Despite numerous reproductive isolating barriers [24], [25], [27]–[29], sympatric populations of *M. guttatus* and *M. nasutus* are still exchanging genes. The low diversity and extensive linkage disequilibrium in *M. nasutus* facilitates straightforward identification of *M. nasutus*-like ancestry in *M. guttatus*, and we use the length-distribution of these blocks to parameterize the recent history of introgression. The many short *M. nasutus* ancestry blocks suggest that its ancestry can persist in *M. guttatus* beyond early generation hybrids, and the length distribution of this ancestry is consistent with more than one pulse of introgression. The genomic distribution of introgression is non-random – in sympatry, absolute interspecific divergence is greater in regions of reduced recombination, suggesting selection against long blocks of *M. nasutus* ancestry in *M. guttatus*. Additional sequencing of individuals in sympatry will help better parameterize the dynamics and extent of introgression from *M. guttatus* to *M. nasutus* and clarify the action of selection for or against admixed ancestry across the genome.

## Materials and Methods

### *Mimulus* sampling and whole genome sequencing

We utilized a combination of existing [downloaded from the NCBI SRA, sequenced by 87] and newly generated whole genome sequence data from 19 different lab and/or naturally inbred *Mimulus* accessions, including 13 *M. guttatus*, 5 *M. nasutus*, and 1 *M. dentilobus* individual as an outgroup (Table S1). Samples varied in their geography and life history. Mean sequencing depths range from 2× to 25×, and read lengths include 36, 76, and 100 base pair paired end reads. We present SRA accession numbers as well as depth, read length and additional sample information in Table S1, and note that we obtained the DPRG sequence data directly from the U.S. Department of Energy Joint Genome Institute. Our analysis included newly generated whole genome sequences from five lines (CACG, CACN, DPRN, NHN, and KOOT), and we present details of sequence generation in Text S1.

### Genome sequence alignment, SNP identification and annotation

We aligned paired end reads to the *M. guttatus* v2.0 reference genome [87] using Burrows-Wheeler Aligner (bwa [32]) with a minimum alignment quality threshold of Q29 (filtering done using SAMtools [88]). Alignment-processing details can be found in Text S1. We produced a high quality set of invariant sites and SNPs simultaneously for all lines using the GATK Unified Genotyper, with a site quality threshold of Q40 [89], [90]. For all analyses described below, we exclusively used genotype calls from reference scaffolds 1–14, corresponding to the 14 chromosomes in the *Mimulus* genome. For all analyses (except PSMC, which requires a consistently high density of data, see below), we set also set a strict minimum depth cutoff of 10 reads per site. To assign genotypes at heterozygous sites, we randomly selected one of two alternate alleles. Such heterozygous sites are not concentrated in long genomic regions and account for approximately 1% and 2% of synonymous SNPs in average focal *M. nasutus* and *M. guttatus* samples, respectively. This translates to individual synonymous heterozygosity of approximately 0.2% and 0.5% in *M. nasutus* and *M. guttatus*, respectively, even before additional filtering to remove misaligned sites (see below). Because sequence diversity is relatively high in our sample, we also analyzed patterns of pairwise sequence diversity using reads aligned with Stampy [33] (expected divergence set to 5%) and an otherwise identical pipeline to that described above. We describe the number of reads mapped with bwa and Stampy for our focal, high coverage lines in Table S7.

To minimize misclassifying mismapped paralogs as SNPs, we then removed triallelic sites and censured genotypes at sites where individual depth was two standard deviations away from mean depth. After these filtering steps, we classified remaining genic loci as zero, two, three, or fourfold degenerate using the *Mimulus guttatus* v2.0 gene annotations provided by phytozome [87]. Compared to bwa, the Stampy pipeline generated quantitatively larger estimates of sequence diversity, but qualitatively similar results (*i.e.*, rank order of pairwise π_S_, Table S2B). Because Stampy doubled individual heterozygosity at synonymous sites, and increased π_N_/π_S_, we believe that it may have mismapped a greater proportion of our reads. Therefore, although Stampy aligned a greater number of reads to the reference genome than bwa (Table S7), we conservatively focus on our bwa alignments for our major analyses. As noted above, none of our qualitative conclusions depend on the read alignment pipeline used.

### Data analysis

In addition to descriptions of our analyses, below and in Text S1, we recreate many analyses, including our PCA, and HMM in a file submitted to Dryad. These analyses can be run on the processed genotypic data for all samples at SNPs used in nj and PCA analyses as well as comparisons between focal samples in 1 kb windows across the genome, all available from doi:10.5061/dryad.vp645.

#### NJ tree and PCA

We first analyzed broad patterns of genomic differentiation captured by principle component analysis and a neighbor-joining (nj) tree for all *M. guttatus* and *M. nasutus* samples. For both these analyses (and the Treemix analyses) we downsampled to 1,000 synonymous SNPs per chromosome with more than one copy of the minor allele (14,000 informative sites in total). In this downsampling we insisted that no focal samples have missing genotypic data and then prioritized by the number of individuals with sequence data (see Text S1). After this downsampling, all individuals had genotypic data at more than 60% of SNPS, and 97% of these SNPs had genotypic data for at least 16 of our 19 individuals.

We construct the nj tree with the nj function in the R [91] package ape [92], and root it by the outgroup, *M. dentilobus*. An nj tree is an empirical description of a distance matrix, rather than a phylogenetic model. As such, they have a long history of use in population genetics [93]–[96], where complex population structure and migration history yields a complex distribution of genealogies across the genome. Indeed, in cases of recent speciation or ongoing gene flow, we expect the specific assumption of a phylogenetic model - that the evolutionary relationships can be described by a strictly bifurcating directed acyclic graph - to be explicitly violated. Perhaps due to the widespread application of nj trees in population genetics, the behavior of admixed populations in nj trees is known (see [97]), and readers can evaluate the nj tree with this knowledge.

We construct our PCA with customized R scripts accounting for missing genotype data described in Text S1. Because all five *M. nasutus* samples are very closely related, they have a large effect on PCA space, as is clear from the observation that PC1 cleanly separates *M. nasutus* and *M. guttatus*. Downsampling to any one *M. nasutus* sample removes this distortion and places *M. nasutus* firmly within southern *M. guttatus* samples (Figure S1).

#### Nucleotide diversity and divergence

Preliminary analyses of nucleotide variation showed a strong influence of sequence features such as read depth and length (Table S3) on diversity estimates. In general, samples with low depth and short reads are less different from all other samples than are samples with high depth and long reads (Tables S3), a likely consequence of difficulties in aligning short reads when they differ substantially from the reference. We therefore focus all following analyses on eight samples with high and consistent mean read depths (13×–24×) and read lengths (all 100 bp paired end reads).

*Divergence and diversity*. We quantified patterns of synonymous and nonsynonymous sequence variation at four-fold and zero-fold degenerate sites, respectively. For each pairwise comparison, we count the number of pairwise sequence differences and number of sites for which both samples have data above our quality and depth thresholds. To generate confidence intervals for our point estimates of diversity and divergence that acknowledge the non-independence of sequence variants due to linkage disequilibrium, we resample 100 kb windows with replacement. We also compared pairwise synonymous differences before and after removing putatively introgressed regions (see Text S1 and Table S8 for more details). Notably, after removing regions of putative introgression, the two southern *M. nasutus* samples were equally diverged from *M. nasutus*, and were both genetically closer to *M. nasutus* than the introgression-censured northern *M. guttatus* samples.

*Allele Frequency Spectrum (AFS). *To polarize the AFS, we examined all sites passing our depth and quality thresholds for M. dentilobus as well as all focal samples. We labeled the M. dentilobus allele as ancestral, and the alternate allele as derived.

*Premature stop codon identification.* We searched for premature stop codons in each *Mimulus* accession using the *Mimulus guttatus* v2.0 gene annotations. We defined a mutant stop codon to be premature only if all three nucleotide sites were available for the codon, if it occurred in a gene for which at least 25% of the codons were available in the sample, and if the codon did not occur in the last 5% of all codons on the 3′ end of the gene.

*Correlations between recombination rate and diversity (or divergence).* We estimated genetic distances in centiMorgans (cM) using information from three existing *Mimulus* genetic linkage maps: one intra-population *M. guttatus* composite map, IMxIM (integrated from three different F_2_ maps between individuals originating from the IM population [98]), and two inter-specific F_2_ maps between IM62 *M. guttatus* (the reference line) and SF *M. nasutus*, IMxSF_2006 [85] and IMxSF_2009 (C. Wu and J. Willis unpublished). All three linkage maps are available at www.mimulusevolution.org. See Text S1 for more details.

With this integrated map, we estimated a local recombination rate for every 100 kb window, smoothed by the mean rate in the surrounding 500 kb. In each window we calculated mean pairwise sequence diversity at synonymous sites and used a non-parametric spearman rank sign test to evaluate the relationship between synonymous sequence diversity and the local recombination rate. We excluded windows with fewer than 100 pairwise comparisons, and regions without recombination estimates. The final number of 100 kb windows included in each pairwise comparison ranged from 1,773–2,023 (∼177.3–202.3 Mb), or 60.5–69.0% of the reference genome. Moreover, the set of windows in each analysis largely overlapped – 1,756 windows were common to all analyses meaning that for a given test 87% to 99% of the windows were used in all other analyses. In Text S1 and Table S6, we show that divergence to *M. dentilobus* and local depth do not influence our qualitative conclusions.

In interpreting our negative relationship between the recombination rate and interspecific divergence across the genome of hybridizing *M. guttatus*, we note that genomic regions with the lowest recombination rates have the highest densities of centromeric and TE-like repeats, and in contrast, high-recombination regions have the highest local gene-densities per physical distance (data not shown). However, there is no obvious mechanistic link between this observation and the consistent negative correlation between divergence and recombination between *M. nasutus* and sympatric, but not allopatric, *M. guttatus*.

*PSMC.* As a complementary inference of historical demography and differentiation, we applied Li and Durbin's implementation of the pairwise sequentially Markovian coalescent (PSMC) [36] to pairwise combinations of focal haploid genomes. We phased the genome of each focal sample by randomly choosing an allele at each heterozygous site; residual heterozygosity was extremely low (see above) therefore we did not expect this to significantly affect our results (see Text S1 for additional details). Due to high diversity in our dataset, we used a window size of 10 bp for PSMC analysis. For all comparisons, we ran PSMC for 20 iterations and used the following input settings: recombination/mutation ratio (r) = 1.25, Tmax = 10, number of intervals (n) = 60, number of population size parameters = 24, parameter distribution pattern = ‘1*4+1*4+1*3+18*2+1*3+1*4+1*6’. We represented time using a generation time of 1 year and a mutation rate of 1.5×10^−8^. We note that choosing a fixed value for the recombination/mutation ratio is appropriate for comparisons between species and within *M. guttatus*; however, this does not capture the change in this ratio accompanying the transition to selfing in *M. nasutus*. Therefore quantitative estimates of population decline through time within *M. nasutus* are best viewed as very rough approximations. To generate a measure of variability in the PSMC estimates, we ran 100 bootstrap analyses for each pairwise comparison (see Text S1 for additional details). Finally, we note that applying PSMC to our Stampy-aligned dataset yielded consistent biological conclusions (see Text S1 and Figures S13, S14, S15, S16) to our bwa dataset.

#### Introgression analyses

*Treemix.* As a test for recent admixture between *M. guttatus* and *M. nasutus*, we used Pickrell and Pritchard's (2012) Treemix method to model the evolutionary history of a group as a series of splits and gene flow events. This method has been previously shown to perform well, even when single individuals represent a group (see Section SI 11 of [99]). For these analyses, we used the same subsample of 14,000 SNPs used for the nj tree and PCA described above. We specified a Treemix block size of 50 SNPs and estimated the evolutionary history including 1, 2, 3 or 4 admixture events. We also ran analyses with and without the reference line IM62 included, and with all nineteen *Mimulus* samples (see Text S1 and results, as well as Figure S10).

*Four population D test for admixture.* Following the notation of Patterson *et al.* [100], we denote sample allele frequencies from populations Y, Z, W and X in lowercase. Here, Y is a sympatric *M. guttatus* population; Z is an allopatric *M. guttatus* population geographically close to Y; W is *M. nasutus*, and X is an allopatric *M. guttatus* population geographically distant from Y. Summing across *i* loci 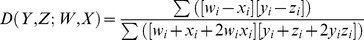
We evaluate significance with a block jackknife, dropping one chromosome in each run [100].

*HMM.* We implement the forward-backward algorithm and posterior decoding as described by Durbin et al. [101] in a customized R script controlling for underflow (available upon request) to calculate posterior probabilities of *M. nasutus* or *M. guttatus* ancestry across all four focal *M. guttatus* samples.

We take as our emissions the minimum pairwise π between our focal *M. guttatus* sample and all *M. nasutus* samples in non-overlapping 1 kb windows (π_close_nas_). We compared π_close_nas_ to the genome-wide distribution of π between a *M. nasutus* sample and its genetically closest *M. nasutus* sample and π between *M. guttatus* samples and the genetically closest *M. nasutus* sample, to calculate the emission probability of admixed or pure *M. guttatus* ancestry, respectively. In doing so we accounted for both the heterogeneity in the number of informative sites across the genome and the fact that we compare each *M. nasutus* sample to three others, while we compare each *M. guttatus* sample to all four *M. nasutus* samples (see Text S1).

We calculate *t_i,j_*, the probability of transitioning from ancestry of species *i* to ancestry from species *j* from α, the admixture proportion, and r, the product of the recombination rate per 1 kb multiplied by a point estimate of the number of generations since admixture. We set *t_i,j_* to *t_gut,gut_* = (1−r)+r (1−α), *t_gut,nas_* = r α, *t_nas,gut_* = r (1−α), and *t_nas,nas_* = (1−r)+r α, and optimize these parameters with the Nelder-Mead algorithm implemented in the R function, optim, calculating the likelihood of our data given these parameters from the forward algorithm. α estimated in this way is similar to estimates from the proportion of low divergence windows presented in the text, suggesting that our data provide information about both α and r. We assume that r is constant across windows, ignoring the influence of the recombination rate on the transition rate.

*Inference of introgression history.* The number of admixture blocks and our point estimate of admixture timing are strong evidence that admixture is not the result of a single chance *M. nasutus* ancestor in the history of these samples. To see this, consider that our current day genome is expected to be broken into *X* chunks *v* generations ago, where *X* is the number of recombination events (*i.e.*, the map length in Morgans which is ∼14.7 in *Mimulus*, times the number of generations, *v*), plus the number of chromosomes (14 in *Mimulus*). So, for example, the CACG genome is broken into *X* = 2,219 chunks at our point estimate of its admixture time, *v* = 150 generations ago (150 generations times 14.7 Morgans plus 14 chromosomes). These *X* chunks are drawn from 2*^v^* genealogical ancestors spread across many ancestors, meaning that CACG is expected to inherit 2,219/2^150^∼1/2^139^ chromosomal segments from a typical genealogical ancestor, and therefore under a point model of admixture the odds of CACG inheriting two ancestry blocks from the same ancestor is vanishingly small. Therefore, each of the 227 *M. nasutus* ancestry blocks observed in CACG descends from a different admixture event, implying a vast number of admixed ancestors in the history of the sympatric populations *M. guttatus*. The case is starker for DPRG, for which we infer a much older point estimate of the admixture time.

Because recombination is a Poisson process, under a single admixture time, *v*, we expect the variance in admixture block length to equal the square of the mean block length. However the variance in admixture block lengths is inconsistent with this expectation for both southern (DPRG, mean = 0.0010 M, σ = 0.0013 M, Bootstrap P<0.001), and northern (CACG, mean = 0.0074 M, σ = 0.0102M, Bootstrap P<0.001) sympatric *M. guttatus* samples. This argues against a point model of admixture and suggests ongoing and sustained gene flow into *M. guttatus* where it is sympatric with *M. nasutus*. We acknowledge that these calculations are somewhat crude, especially because we assume a constant recombination rate genome-wide. Nonetheless, the extreme variation in admixture block length and the large number of visually obvious blocks in Figure S9 supports this qualitative result.

We note that since our inference of the extreme improbability that two admixture blocks are derived from the same introgression event relied on a point model, we must soften this conclusion. It is plausible that a few young ancestry blocks in CACG are descended from the same admixture event, however, the rejection of a point admixture model strengthens our major conclusion that gene flow is ongoing and that our samples do not represent single admixture events.

#### Inference of introgression from *M. guttatus* to *M. nasutus*

To test for introgression from *M. guttatus* into *M. nasutus*, we took advantage of the structure of genetic variation in *M. nasutus* – for most of the genome all individuals are remarkably similar, and when this is not the case, one individual often differs sharply from all others. The genetic variation in such genomic regions has either been maintained from the stock of ancestral variation, or it has been reintroduced by introgression upon secondary contact. The extreme genetic variation and miniscule extent of linkage disequilibrium within *M. guttatus* makes these two alternative hypotheses nearly indistinguishable in any given region; however, by collating information across all such regions we can test the introgression hypothesis.

To do so, we find long regions (20 kb) of the *M. nasutus* genome for which one individual is a genetic outlier, as described above. In these regions, we ask whether the outlier is more closely related to a specific *M. guttatus* sample than are the non-outliers. Under incomplete lineage sorting, there is a 50% probability that this is the case. However, under an admixture model this probability is greater than 50% if the *M. guttatus* sample is more closely related to the potential admixture source than it is to the population that founded *M. nasutus*. See Text S1 for more details, and Table S5 for the robustness of this inference to our specific rules for identifying outlier windows.

We perform this test for all *M. nasutus* samples individually against two focal allopatric *M. guttatus* samples from the northern (AHQT) and southern (SLP) groups. In addition to testing the one sided hypothesis of whether the outlier sample is more often like a given *M. guttatus* sample, we also pool our northern *M. nasutus* samples to amplify any signal.

## Supporting Information

Figure S1 Principal component analysis after downsampling to a single *M. nasutus* individual. All five *M. nasutus* samples (each plotted in ***A–E***) consistently cluster within southern *M. guttatus*.
(PDF)

Figure S2 PSMC estimates of population diversity and divergence through time, showing the full range of recent potential maximum population sizes. Samples are identical to those in Figure 1E.
(PDF)

Figure S3 PSMC estimate of the split date between *M. nasutus* and southern *M. guttatus*. We infer speciation to occur when the between species curve (SLP×KOOT, gray) diverges from the southern *M. guttatus* curve (SLP×DPRG, blue). The black/dark gray line showing greater effective population size between *M. nasutus* and northern *M. guttatus* is shown for comparison.
(PDF)

Figure S4 PSMC inference shows *M. nasutus*’ decline in effective population size. Effective population size through time is shown for pseudo-diploid genomes for all six pair-wise combinations of the four focal *M. nasutus* individuals. One intraspecific *M. guttatus* pair (AHQT×SLP, blue line) is shown for comparison.
(PDF)

Figure S5 PSMC suggests shared ancestry (in the form of a decrease in *N_e_*) between *M. nasutus* and sympatric *M. guttatus* due to gene flow in sympatry. Population size through time is shown for pseudo-diploid genomes for pair-wise combinations of *M. guttatus* and/or *M. nasutus* individuals. Blue and violet = intraspecific *M. guttatus*. Black/gray = between species comparisons with allopatric *M. guttatus*. Brown/salmon and dark gold = interspecific comparisons with sympatric *M. guttatus*. Red = intraspecific *M. nasutus*.
(PDF)

Figure S6 PSMC suggests shared ancestry between *M. nasutus* and sympatric *M. guttatus*. This figure is zoomed out for scale. Samples are identical to those in Figure S4.
(PDF)

Figure S7 The allele frequency spectrum. The proportion of derived polymorphisms observed in one two or three (x-axis) *M. guttatus* (blue) and *M. nasutus* (red) samples. Filled and hatched bars describe four and zero-fold degenerate positions, respectively, while error bars indicate the upper and lower 2.5% of tails of the block bootstrap distribution.
(PDF)

Figure S8 Histograms of pairwise sequence variation (π). π within and between species in overlapping windows of varying size from 1 kb to 100 kb (A–F). For interspecific comparisons we focus only on allopatric *M. guttatus* populations. Dotted lines denote π<0.5%.
(PDF)

Figure S9 Recent coalescence between focal samples and alternative *M. nasutus* across all chromosomes. Moving along each chromosome, we color genomic regions in which the focal individual and a *M. nasutus* sample (indicated by color) recently coalesce (π_s_≤0.5%). White regions coalesce more distantly in the past (π_s_>0.5%) and gray regions indicate insufficient density of called sites. Tick marks on the x-axis indicate 1 MB. For *M. nasutus* samples, colored regions represent common ancestry since the species split. Regions of *M. guttatus* genomes recently coalescing with *M. nasutus* likely represent recent introgression. Recent coalescence across chromosomes 1–5 (***A***), 6–10 (***B***) and 11–14 (***C***), and S9C presents recent coalescence across chromosomes 10–14. Note that since we insist on a high density of synonymous sites to evaluate ‘recent coalescence’ only a subset of our data informs our questions of recent coalescence. However, our HMM makes use of information from all genomic regions, and conditions on the density of sites with genotype data. Therefore, we can label introgression regions in places here we do not evaluate recent coalescence – i.e. purple lines indicating introgression into *M. guttatus* samples in gray regions of Figure S9.
(PDF)

Figure S10 Alternative Treemix analyses. We present all Treemix analyses varying data subset and number of admixture arrows. Left to Right: (***A***) Focal samples+the reference, rooted by the outgroup (MDENT) (***B***) Focal samples rooted by the outgroup (MDENT), or (***C***) All samples rooted by the outgroup (MDENT). From top to bottom: (***1***) one, (***2***) two, (***3***) three, or (***4***) four admixture events.
(PDF)

Figure S11 Histograms of pairwise sequence divergence to *M. nasutus*. Shown in overlapping windows of varying size, by individual *M. guttatus* sample. (***A–F***) show window sizes from 1 kb to 100 kb. Dotted lines denote π_S_<0.5%.
(PDF)

Figure S12 The admixture block length distribution under alternative post-hoc ‘healing’ rules. The number of admixed blocks (as inferred by a greater than 95% posterior probability of *M. nasutus* ancestry from our HMM) longer than x. We joined two admixture blocks within (***A***) 0, (***B***) 20, (***C***) 50, or (***D***) 100 kb.
(PDF)

Figure S13 PSMC inference using Stampy-aligned data shows similar patterns of population structure within *M. guttatus* and population size decline within *M. nasutus* compared to bwa-aligned data. Here, and in Figures S14, S15, S16, Stampy PSMC trajectories are overlaid over original bwa trajectories+bootstraps for identical sample comparisons.
(PDF)

Figure S14 Stampy PSMC trajectories show a similar rate of species divergence over time compared to bwa trajectories. However, inferred population sizes are consistently larger from the Stampy than the bwa pipeline. This result is consistent with higher estimated levels of sequence diversity and divergence from Stampy.
(PDF)

Figure S15 Stampy PSMC trajectories – speciation time. Both Stampy and bwa pipelines provide qualitatively consistent estimates for the time of speciation between *M. guttatus* and *M. nasutus* compared to bwa trajectories.
(PDF)

Figure S16 Stampy PSMC trajectories – admixture. Stampy inferred psmc trajectories reveal a similar effect of geography and admixture on species divergence compared to bwa trajectories.
(PDF)

Table S1 Detailed information about the biology, geography, inbreeding and sequencing of each sample analyzed in this manuscript. Gray-shaded rows denote focal samples used in our primary analyses.
(DOCX)

Table S2 Pairwise sequence comparisons between samples. (***A***) For all pairwise comparisons, we report the identifiers of our samples, the mean number of pairwise sequence differences at fourfold degenerate sites (π_S_), the ratio of diversity at fully constrained and fourfold degenerate sites (π_N_/π_S_), the type of comparison (with regard to the population and species sample), and the number of focal samples involved in the comparison. (***B***) All pairwise π_S_ and π_N_/π_S_ values for focal samples as obtained from bwa and (Stampy) between focal comparisons.
(DOCX)

Table S3 A summary of pairwise sequence diversity in comparisons between all samples. We split this summary by the number of focal samples and the class of population comparison, and present both the mean number of pairwise sequence differences at fourfold degenerate sites (π_S_), and the ratio of diversity at fully constrained and fourfold degenerate sites (π_N_/π_S_).
(DOCX)

Table S4 Summary of the admixture block-length distribution and the robustness of our inference of admixture history. For each focal sample, we present (***1***) The inferred number of blocks of *M. nasutus* ancestry (***2***) The total length (in kb) of *M. nasutus* ancestry inferred in the focal sample, (***3***) The mean block length in centiMorgans = mean_block_length[bp] * total_map_length[cM]/genome_size[bp], where total_map_length ∼1,470 cM and total_genome_size ∼2.6*10^8^. (***4***) The standard deviation in block length (in cM). (***5***) ***T***, the expected number of generations since admixture = 1/mean_length(cM). (***6***) P(exp) – A one sided test of the hypothesis that our block length distribution is not more variable than expected under one admixture pulse at time ***T***. We obtained this probability by resampling blocks with replacement 1,000 times and finding the proportion of resampling experiments with mean block lengths greater than the variance in block lengths. (***7***) The probability that ancestry from an admixture event at time ***T*** is maintained until the present. This low probability (never greater than 10^−10^), argues against a model of *M. nasutus* ancestry in CACG or in DPRG being derived from a small number of introgression events. We explored numerous post-hoc strategies to ensure the robustness of our inference to idiosyncrasies in the identification of contiguous admixture blocks. We first attempted to ‘heal’ physically close blocks into longer blocks. Specifically we connect admixture blocks separated by 0, 20, 50 or 100 kb into one longer block (noted in column, ‘Heal’). We also control for the potential of accidentally labeling regions of low divergence as introgressed by removing blocks whose length matches those identified in our allopatric samples, SLP or AHQT (noted in the column, ‘Control for short blocks’).
(DOCX)

Table S5 Inference of introgression from *M. guttatus* to *M. nasutus* is robust to alternative thresholds of identifying outlier regions. The number of genomic regions where the outlier *M. nasutus* sample (or collection of samples) is closer to the *M. guttatus* sample of interest than is the average *M. nasutus* sample is presented before the slash and the total number of outlier regions with informative data is given after the slash. Note that the total number of outlier regions for a given *M. nasutus* sample may differ in comparisons to AHQT and SLP due to different patterns of missing data. π_S_ and ***L*** denote the threshold π_S_ and the sliding window size used to identity 20 kb outlier regions, respectively (see Text S1 for more details). Light gray shading represents the intermediate parameter values for identifying outliers regions reported in the main text.
(DOCX)

Table S6 The negative relationship between recombination rate and divergence between *M. nasutus* and sympatric *M. guttatus* is not driven by sequencing depth or divergence to *M. dentilobus*. The first two columns present the percentage of fourfold degenerate sites that differ in the comparison (noted in the row heading) in regions with lower (low rec) and higher (high rec) than median recombination rates. The subsequent columns describe Spearman's ρ and the P-value associated with this nonparametric correlation coefficient with alternative controls described in the supplement.
(DOCX)

Table S7 Read alignment. Number of reads aligned (at q29) with bwa and Stampy for our focal lines.
(DOCX)

Table S8 After removing regions of recent introgression, interspecific divergence corresponds to the topology of the neighbor-joining tree. We present the percent of fourfold degenerate sites that differ between samples before (above the main diagonal) and after (below the main diagonal) removing regions inferred to be recently introgressed. Note that while CACG is much closer to *M. nasutus* samples before removing introgressed regions than AHQT, these two samples are equally differentiated from *M. nasutus* after removing regions of introgression.
(DOCX)

Text S1 Detailed descriptions of data generation, analyses, and tests of the robustness of major results outlined in the main text. This includes details of sequencing, alignment/read mapping, neighbor joining trees, PCA, and Treemix analyses. We also provide details of the implementation of our Hidden Markov Model, information regarding the recombination map used in our analyses, and our analysis of the relationship between local recombination rates and genetic variation. We include descriptions of additional PSMC results, including those between *M. nasutus* and sympatric *M. nasutus*. We evaluate the robustness of our major conclusions regarding introgression, by (***1***) providing additional Treemix analyses, (***2***) Analyzing block length distributions under alternative “healing” rules, and (***3***) Controlling for potential confounds of the observed relationship between pairwise sequence divergence and the local recombination rate. We also describe how the local recombination rate varies with genomic content.
(DOCX)
