# Supplementary figures and images for: Speciation and Introgression between Mimulus nasutus and Mimulus guttatus
Source: PLoS Genet. 2014 Jun 26;10(6):e1004410. doi: 10.1371/journal.pgen.1004410 (PMC4072524; doi:10.1371/journal.pgen.1004410)

**A) PCA, focal nas = CACN**

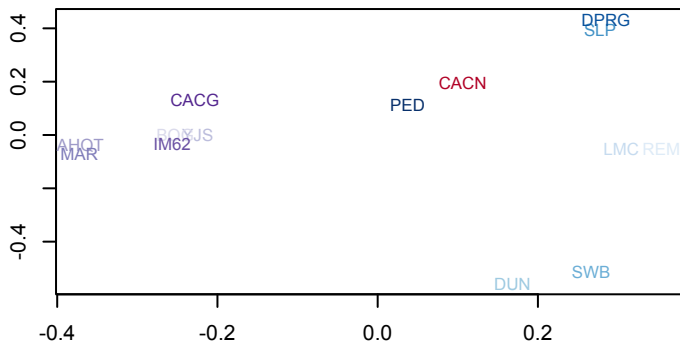

**B) PCA, focal nas = SF**

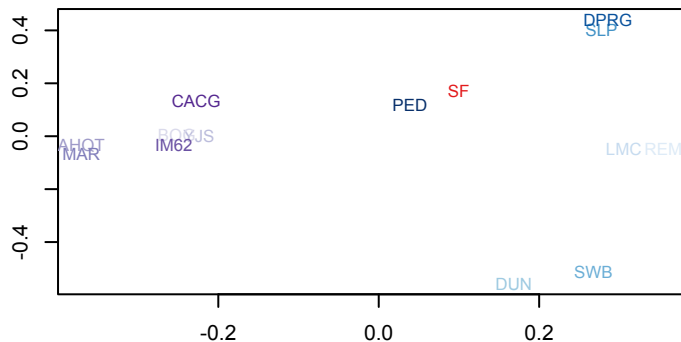

**C) PCA, focal nas = NHN**

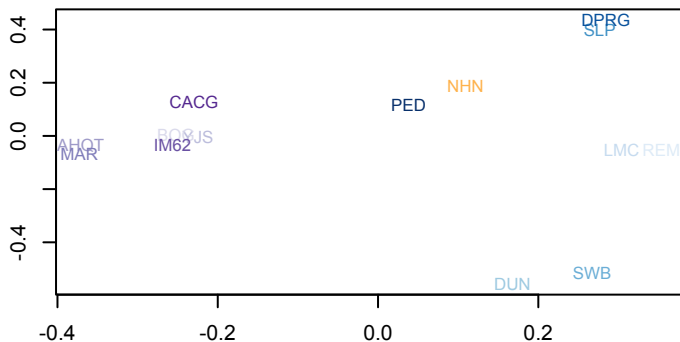

**D) PCA, focal nas = Koot**

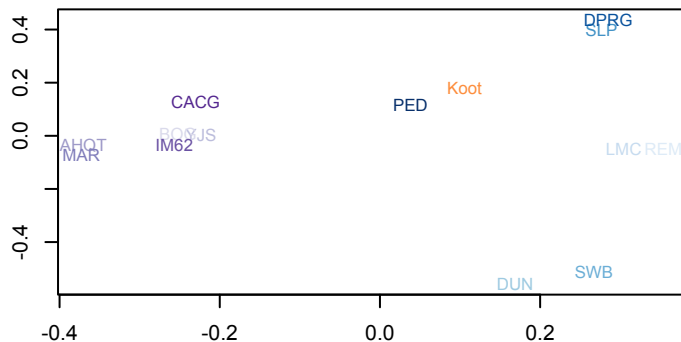

**E) PCA, focal nas = DPRN**

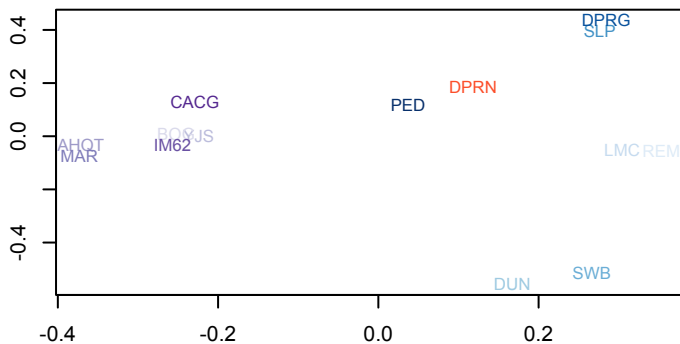

Supplement: Figure S1 — Principal component analysis after downsampling to a single M. nasutus individual. All five M. nasutus samples (each plotted in A–E) consistently cluster within southern M. guttatus. (PDF) [file pgen.1004410.s001.pdf]

Effective population size ( $\times 10^4$ )

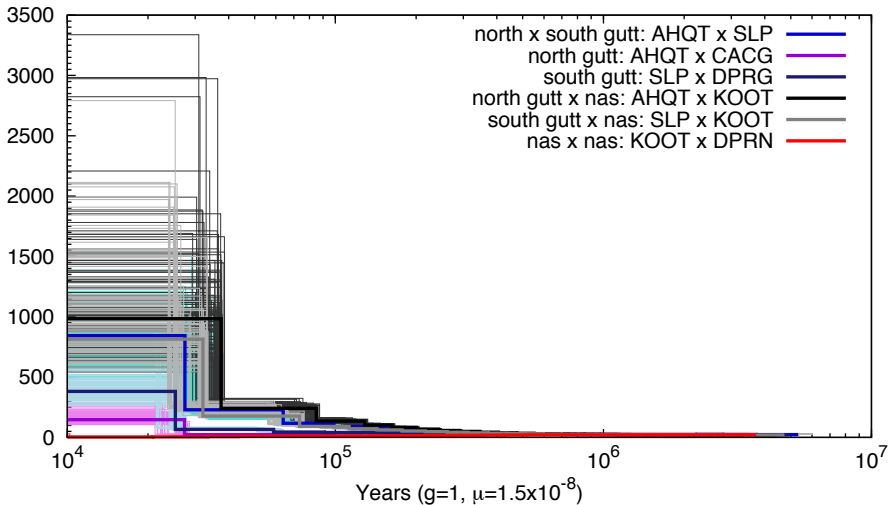

Supplement: Figure S2 — PSMC estimates of population diversity and divergence through time, showing the full range of recent potential maximum population sizes. Samples are identical to those in Figure 1E. (PDF) [file pgen.1004410.s002.pdf]

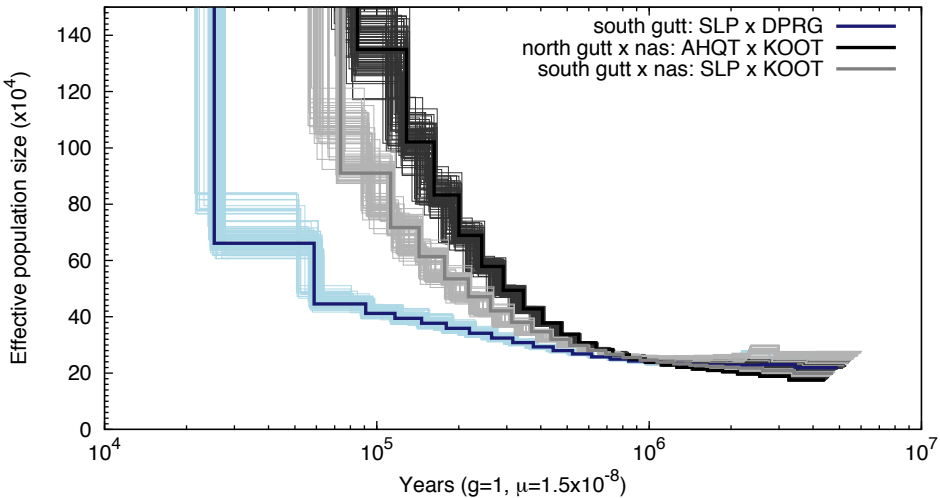

Supplement: Figure S3 — PSMC estimate of the split date between M. nasutus and southern M. guttatus. We infer speciation to occur when the between species curve (SLP×KOOT, gray) diverges from the southern M. guttatus curve (SLP×DPRG, blue). The black/dark gray line showing greater effective population size between M. nasutus and northern M. guttatus is shown for comparison. (PDF) [file pgen.1004410.s003.pdf]

Effective population size ( $\times 10^4$ )

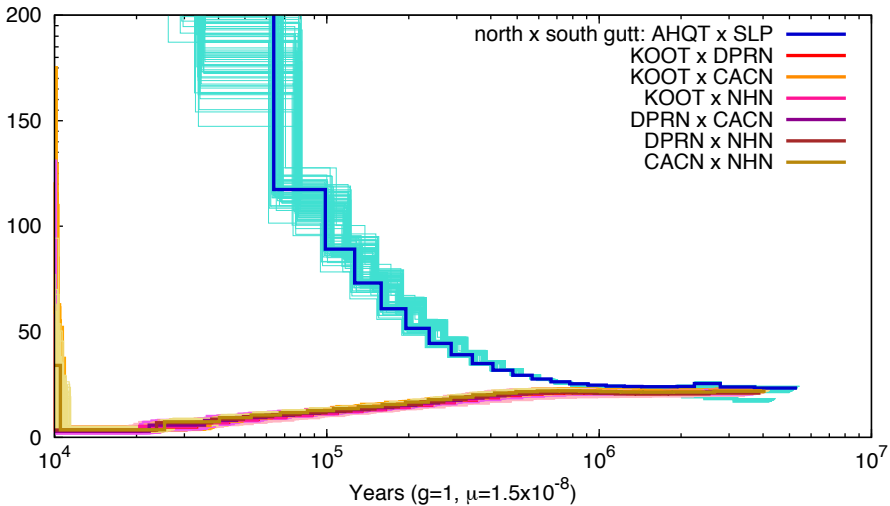

Supplement: Figure S4 — PSMC inference shows M. nasutus’ decline in effective population size. Effective population size through time is shown for pseudo-diploid genomes for all six pair-wise combinations of the four focal M. nasutus individuals. One intraspecific M. guttatus pair (AHQT×SLP, blue line) is shown for comparison. (PDF) [file pgen.1004410.s004.pdf]

Effective population size ( $\times 10^4$ )

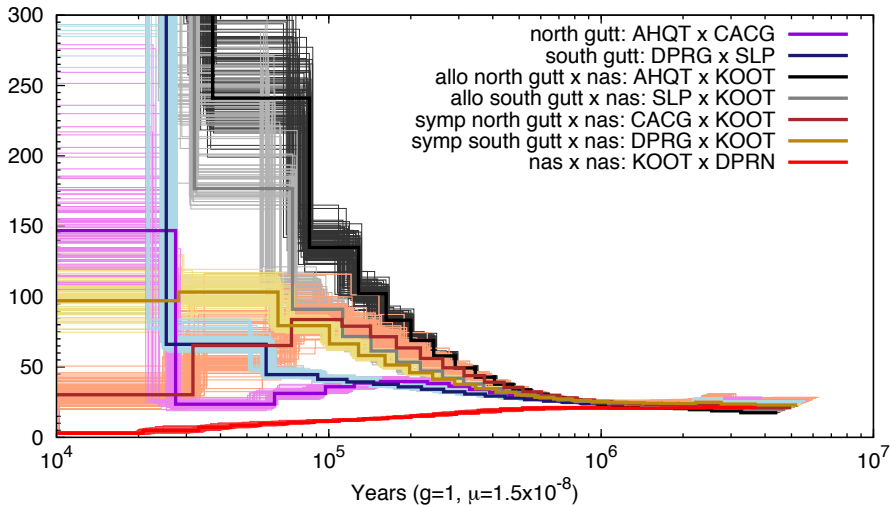

Supplement: Figure S5 — PSMC suggests shared ancestry (in the form of a decrease in Ne) between M. nasutus and sympatric M. guttatus due to gene flow in sympatry. Population size through time is shown for pseudo-diploid genomes for pair-wise combinations of M. guttatus and/or M. nasutus individuals. Blue and violet = intraspecific M. guttatus. Black/gray = between species comparisons with allopatric M. guttatus. Brown/salmon and dark gold = interspecific comparisons with sympatric M. guttatus. Red = intraspecific M. nasutus. (PDF) [file pgen.1004410.s005.pdf]

Effective population size ( $\times 10^4$ )

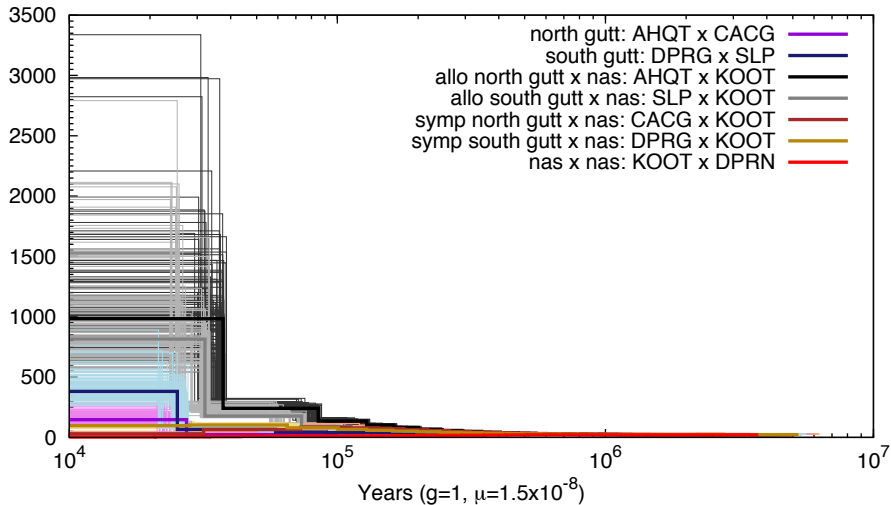

Supplement: Figure S6 — PSMC suggests shared ancestry between M. nasutus and sympatric M. guttatus. This figure is zoomed out for scale. Samples are identical to those in Figure S4. (PDF) [file pgen.1004410.s006.pdf]

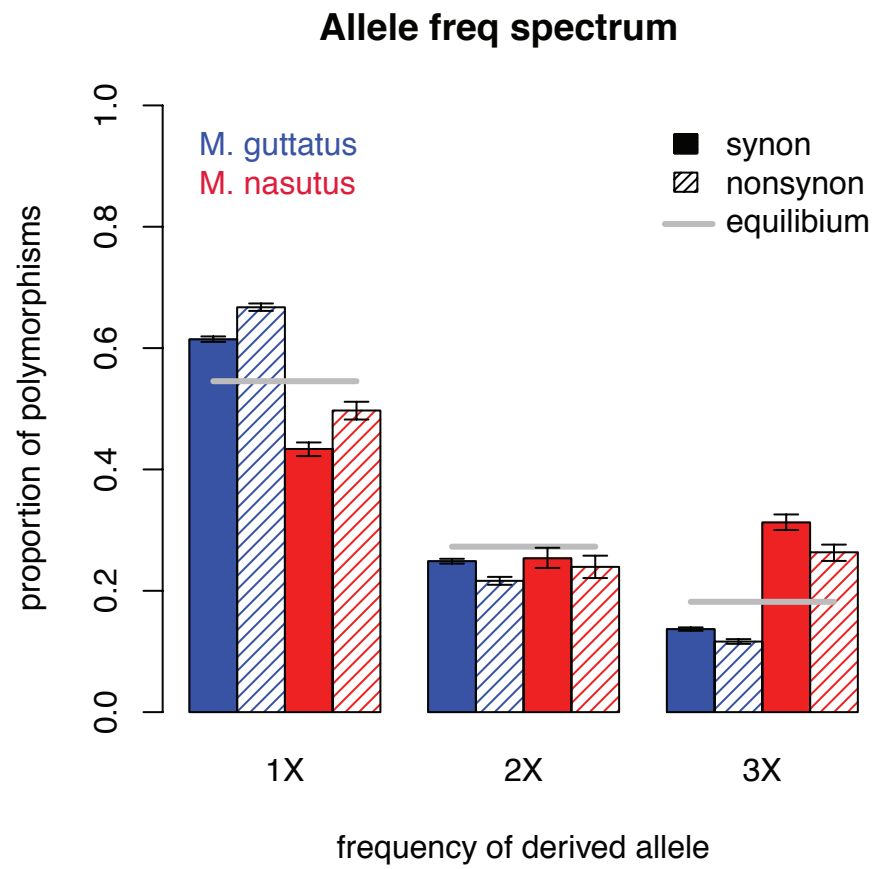

Supplement: Figure S7 — The allele frequency spectrum. The proportion of derived polymorphisms observed in one two or three (x-axis) M. guttatus (blue) and M. nasutus (red) samples. Filled and hatched bars describe four and zero-fold degenerate positions, respectively, while error bars indicate the upper and lower 2.5% of tails of the block bootstrap distribution. (PDF) [file pgen.1004410.s007.pdf]

Effective population size ( $\times 10^4$ )

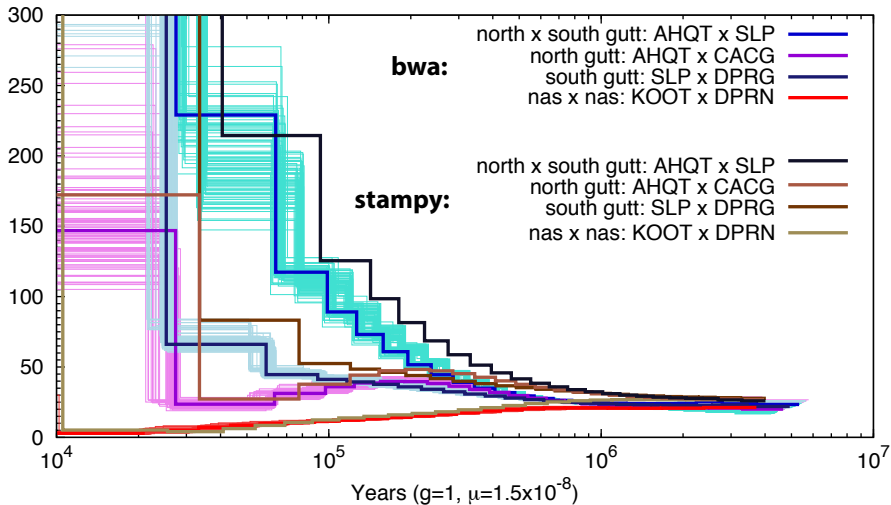

Supplement: Figure S13 — PSMC inference using Stampy-aligned data shows similar patterns of population structure within M. guttatus and population size decline within M. nasutus compared to bwa-aligned data. Here, and in Figures S14, S15, S16, Stampy PSMC trajectories are overlaid over original bwa trajectories+bootstraps for identical sample comparisons. (PDF) [file pgen.1004410.s013.pdf]

Effective population size ( $\times 10^4$ )

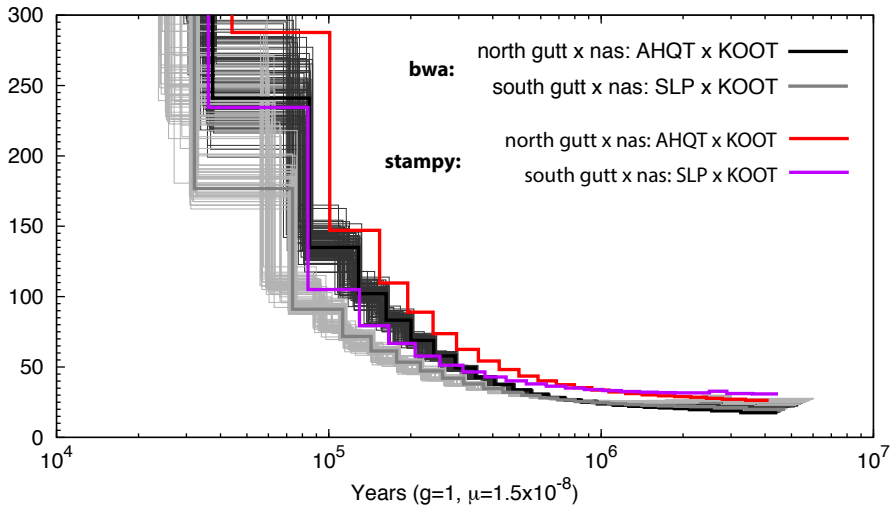

Supplement: Figure S14 — Stampy PSMC trajectories show a similar rate of species divergence over time compared to bwa trajectories. However, inferred population sizes are consistently larger from the Stampy than the bwa pipeline. This result is consistent with higher estimated levels of sequence diversity and divergence from Stampy. (PDF) [file pgen.1004410.s014.pdf]

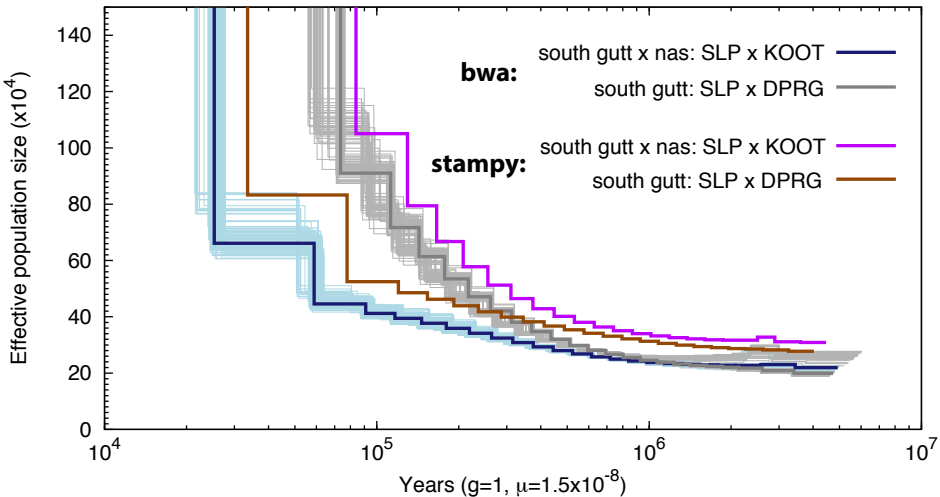

Supplement: Figure S15 — Stampy PSMC trajectories – speciation time. Both Stampy and bwa pipelines provide qualitatively consistent estimates for the time of speciation between M. guttatus and M. nasutus compared to bwa trajectories. (PDF) [file pgen.1004410.s015.pdf]

Effective population size ( $\times 10^4$ )

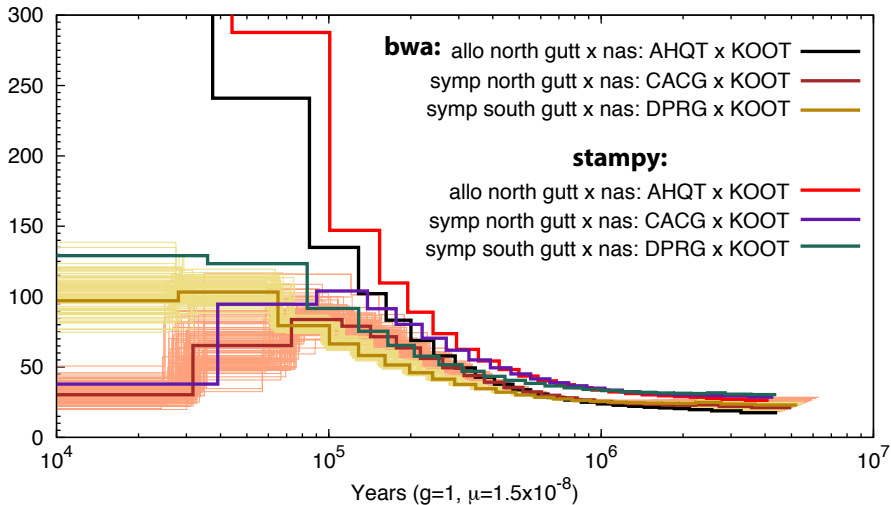

Supplement: Figure S16 — Stampy PSMC trajectories – admixture. Stampy inferred psmc trajectories reveal a similar effect of geography and admixture on species divergence compared to bwa trajectories. (PDF) [file pgen.1004410.s016.pdf]
