# Supplementary material for: Speciation and Introgression between Mimulus nasutus and Mimulus guttatus
Source: PLoS Genet. 2014 Jun 26;10(6):e1004410. doi: 10.1371/journal.pgen.1004410 (PMC4072524; doi:10.1371/journal.pgen.1004410)
Supplement: Figure S8 — Histograms of pairwise sequence variation (π). π within and between species in overlapping windows of varying size from 1 kb to 100 kb (A–F). For interspecific comparisons we focus only on allopatric M. guttatus populations. Dotted lines denote π<0.5%. (PDF) [file pgen.1004410.s008.pdf]

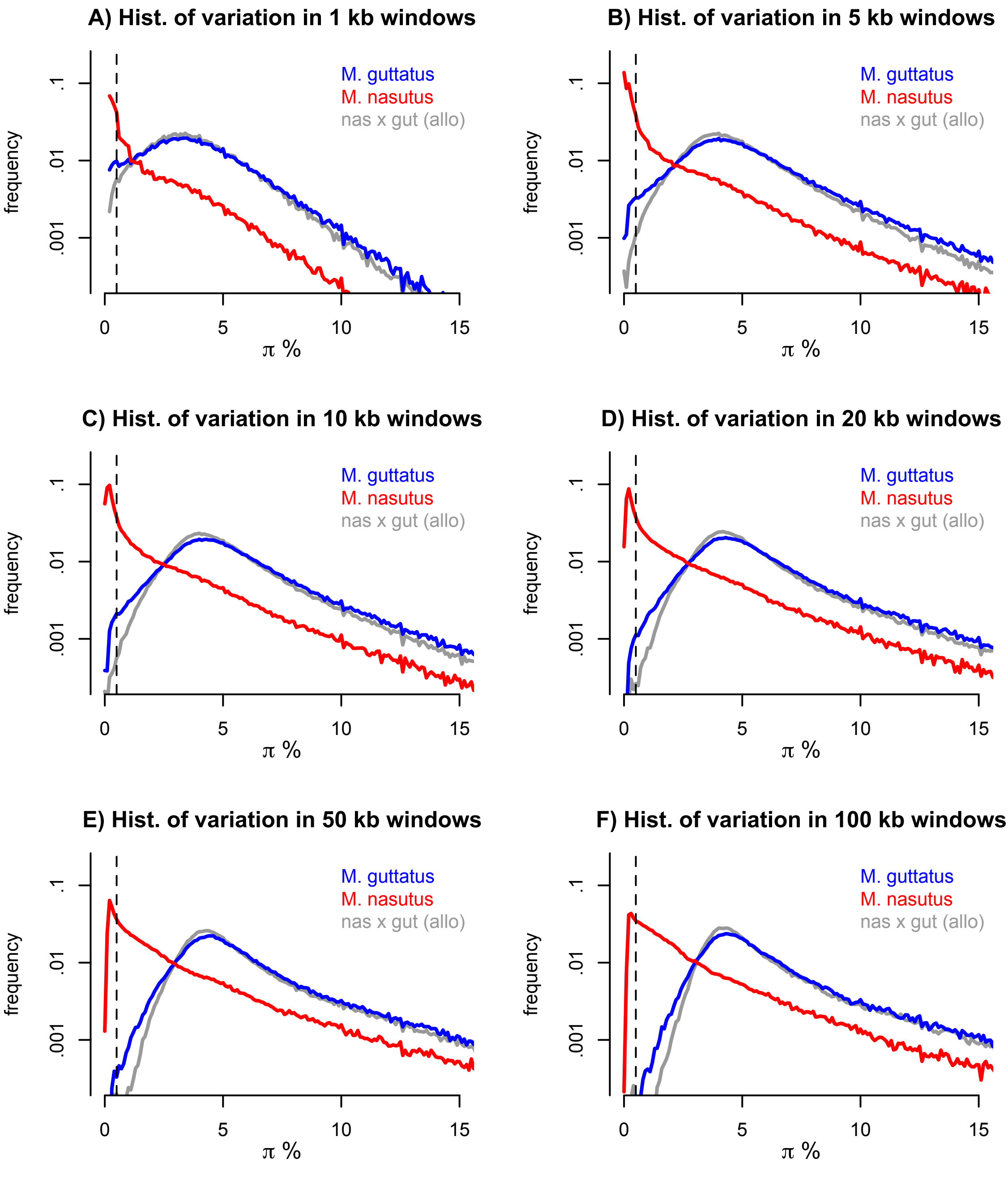
A) Hist. of variation in 1 kb windows
B) Hist. of variation in 5 kb windows
C) Hist. of variation in 10 kb windows
D) Hist. of variation in 20 kb windows
E) Hist. of variation in 50 kb windows
F) Hist. of variation in 100 kb windows
M. guttatus
M. nasutus
nas x gut (allo)
frequency
.1
.01
.001
0
5
10
15
π %
