# Supplementary material for: Speciation and Introgression between Mimulus nasutus and Mimulus guttatus
Source: PLoS Genet. 2014 Jun 26;10(6):e1004410. doi: 10.1371/journal.pgen.1004410 (PMC4072524; doi:10.1371/journal.pgen.1004410)
Supplement: Figure S9 — Recent coalescence between focal samples and alternative M. nasutus across all chromosomes. Moving along each chromosome, we color genomic regions in which the focal individual and a M. nasutus sample (indicated by color) recently coalesce (πs≤0.5%). White regions coalesce more distantly in the past (πs>0.5%) and gray regions indicate insufficient density of called sites. Tick marks on the x-axis indicate 1 MB. For M. nasutus samples, colored regions represent common ancestry since the species split. Regions of M. guttatus genomes recently coalescing with M. nasutus likely represent recent introgression. Recent coalescence across chromosomes 1–5 (A), 6–10 (B) and 11–14 (C), and S9C presents recent coalescence across chromosomes 10–14. Note that since we insist on a high density of synonymous sites to evaluate ‘recent coalescence’ only a subset of our data informs our questions of recent coalescence. However, our HMM makes use of information from all genomic regions, and conditions on the density of sites with genotype data. Therefore, we can label introgression regions in places here we do not evaluate recent coalescence – i.e. purple lines indicating introgression into M. guttatus samples in gray regions of Figure S9. (PDF) [file pgen.1004410.s009.pdf]

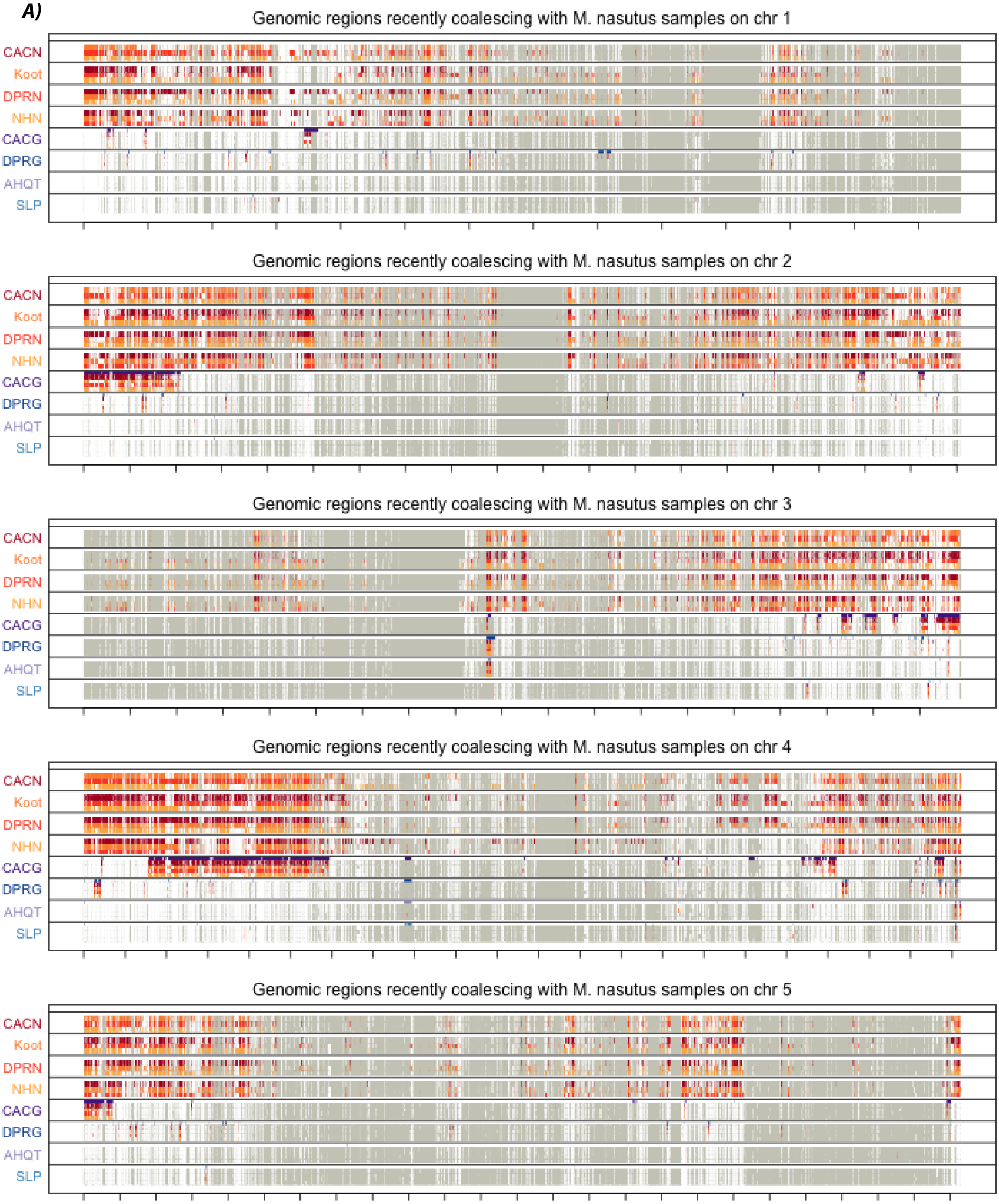
A)
Genomic regions recently coalescing with M. nasutus samples on chr 1
CACN
Koot
DPRN
NHN
CACG
DPRG
AHQT
SLP
Genomic regions recently coalescing with M. nasutus samples on chr 2
CACN
Koot
DPRN
NHN
CACG
DPRG
AHQT
SLP
Genomic regions recently coalescing with M. nasutus samples on chr 3
CACN
Koot
DPRN
NHN
CACG
DPRG
AHQT
SLP
Genomic regions recently coalescing with M. nasutus samples on chr 4
CACN
Koot
DPRN
NHN
CACG
DPRG
AHQT
SLP
Genomic regions recently coalescing with M. nasutus samples on chr 5
CACN
Koot
DPRN
NHN
CACG
DPRG
AHQT
SLP

B)

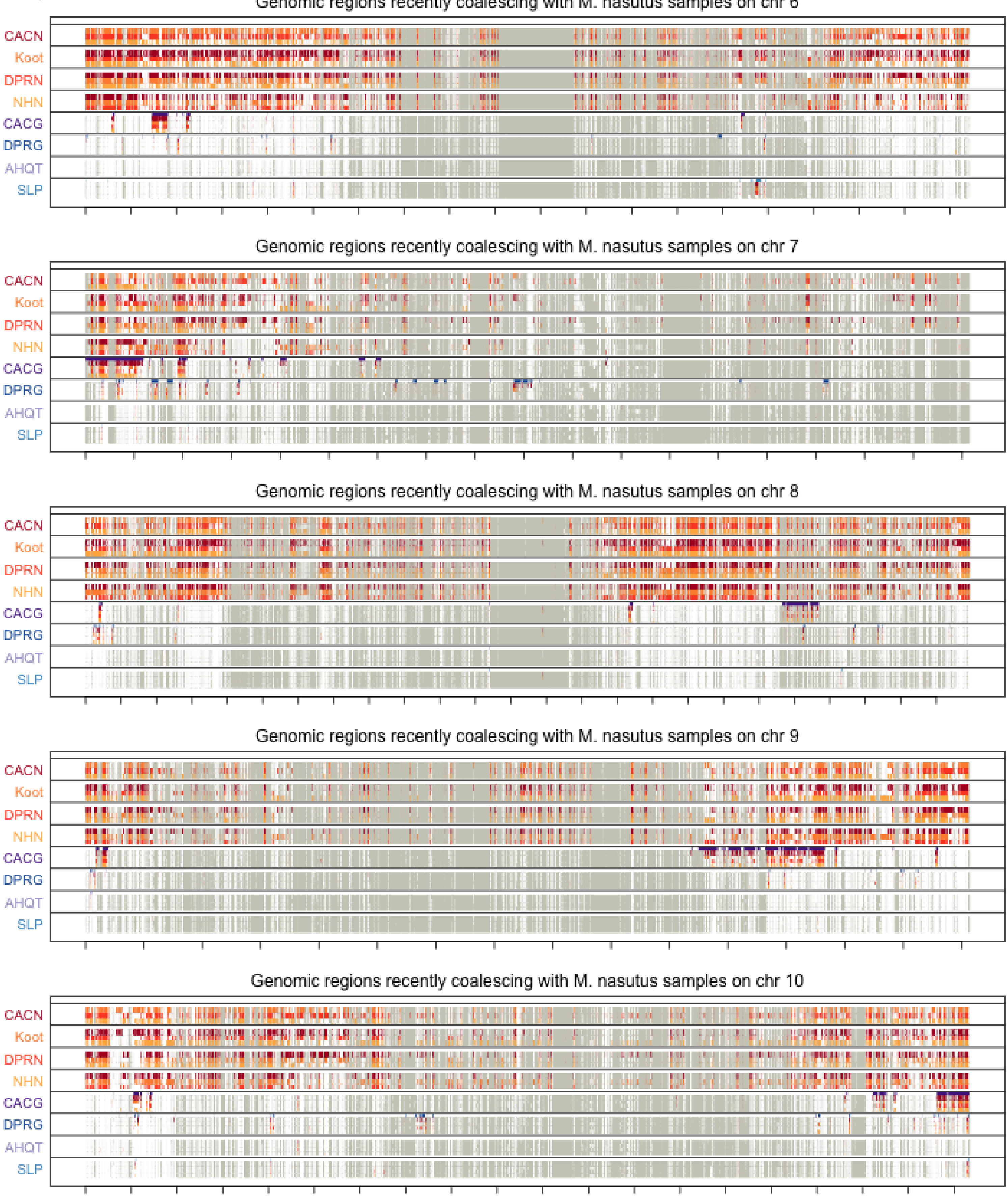
Genomic regions recently coalescing with M. nasutus samples on chr 6
CACN
Koot
DPRN
NHN
CACG
DPRG
AHQT
SLP
Genomic regions recently coalescing with M. nasutus samples on chr 7
CACN
Koot
DPRN
NHN
CACG
DPRG
AHQT
SLP
Genomic regions recently coalescing with M. nasutus samples on chr 8
CACN
Koot
DPRN
NHN
CACG
DPRG
AHQT
SLP
Genomic regions recently coalescing with M. nasutus samples on chr 9
CACN
Koot
DPRN
NHN
CACG
DPRG
AHQT
SLP
Genomic regions recently coalescing with M. nasutus samples on chr 10
CACN
Koot
DPRN
NHN
CACG
DPRG
AHQT
SLP

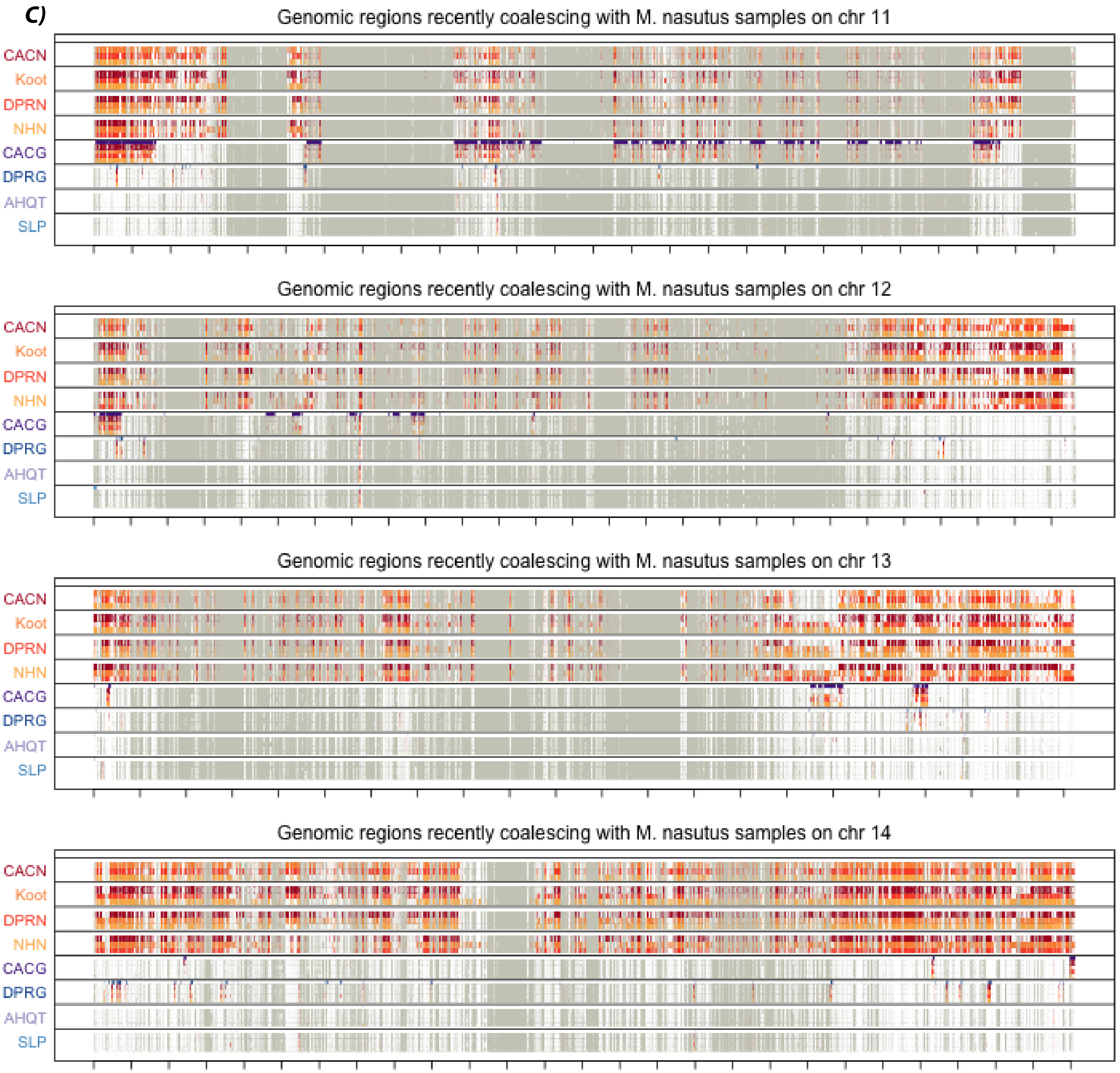
C)
Genomic regions recently coalescing with M. nasutus samples on chr 11
CACN
Koot
DPRN
NHN
CACG
DPRG
AHQT
SLP
Genomic regions recently coalescing with M. nasutus samples on chr 12
CACN
Koot
DPRN
NHN
CACG
DPRG
AHQT
SLP
Genomic regions recently coalescing with M. nasutus samples on chr 13
CACN
Koot
DPRN
NHN
CACG
DPRG
AHQT
SLP
Genomic regions recently coalescing with M. nasutus samples on chr 14
CACN
Koot
DPRN
NHN
CACG
DPRG
AHQT
SLP
