# Supplementary material for: Speciation and Introgression between Mimulus nasutus and Mimulus guttatus
Source: PLoS Genet. 2014 Jun 26;10(6):e1004410. doi: 10.1371/journal.pgen.1004410 (PMC4072524; doi:10.1371/journal.pgen.1004410)
Supplement: Figure S10 — Alternative Treemix analyses. We present all Treemix analyses varying data subset and number of admixture arrows. Left to Right: (A) Focal samples+the reference, rooted by the outgroup (MDENT) (B) Focal samples rooted by the outgroup (MDENT), or (C) All samples rooted by the outgroup (MDENT). From top to bottom: (1) one, (2) two, (3) three, or (4) four admixture events. (PDF) [file pgen.1004410.s010.pdf]

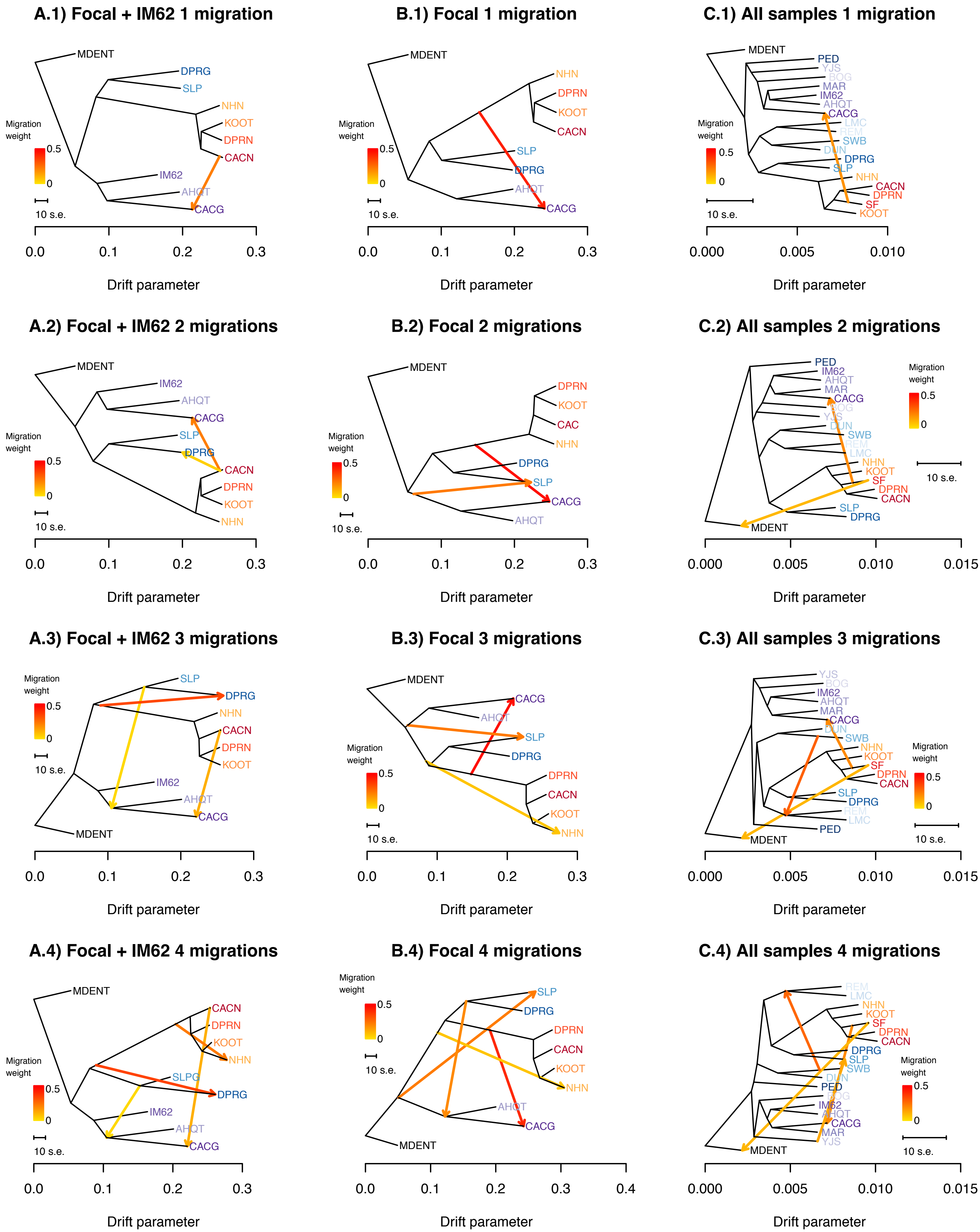
A.1) Focal + IM62 1 migration
MDENT
DPRG
SLP
NHN
KOOT
DPRN
CACN
IM62
AHQT
CACG
Migration weight
0.5
0
10 s.e.
0.0
0.1
0.2
0.3
Drift parameter
B.1) Focal 1 migration
MDENT
NHN
DPRN
KOOT
CACN
SLP
DPRG
AHQT
CACG
Migration weight
0.5
0
10 s.e.
0.0
0.1
0.2
0.3
Drift parameter
C.1) All samples 1 migration
MDENT
PED
YJS
BOG
MAR
IM62
AHQT
CACG
LMC
REM
SWB
DUN
DPRG
SLP
NHN
CACN
DPRN
SF
KOOT
Migration weight
0.5
0
10 s.e.
0.000
0.005
0.010
Drift parameter
A.2) Focal + IM62 2 migrations
MDENT
IM62
AHQT
CACG
SLP
DPRG
CACN
DPRN
KOOT
NHN
Migration weight
0.5
0
10 s.e.
0.0
0.1
0.2
0.3
Drift parameter
B.2) Focal 2 migrations
MDENT
DPRN
KOOT
CAC
NHN
DPRG
SLP
CACG
AHQT
Migration weight
0.5
0
10 s.e.
0.0
0.1
0.2
0.3
Drift parameter
C.2) All samples 2 migrations
PED
IM62
AHQT
MAR
CACG
BOG
YJS
DUN
SWB
REM
LMC
NHN
KOOT
SF
DPRN
CACN
SLP
DPRG
MDENT
Migration weight
0.5
0
10 s.e.
0.000
0.005
0.010
0.015
Drift parameter
A.3) Focal + IM62 3 migrations
Migration weight
0.5
0
10 s.e.
SLP
DPRG
NHN
CACN
DPRN
KOOT
IM62
AHQT
CACG
MDENT
0.0
0.1
0.2
0.3
Drift parameter
B.3) Focal 3 migrations
MDENT
CACG
AHQT
SLP
DPRG
DPRN
CACN
KOOT
NHN
Migration weight
0.5
0
10 s.e.
0.0
0.1
0.2
0.3
Drift parameter
C.3) All samples 3 migrations
YJS
BOG
IM62
AHQT
MAR
CACG
DUN
SWB
NHN
KOOT
SF
DPRN
CACN
SLP
DPRG
REM
LMC
PED
MDENT
Migration weight
0.5
0
10 s.e.
0.000
0.005
0.010
0.015
Drift parameter
A.4) Focal + IM62 4 migrations
MDENT
CACN
DPRN
KOOT
NHN
SLPG
DPRG
IM62
AHQT
CACG
Migration weight
0.5
0
10 s.e.
0.0
0.1
0.2
0.3
Drift parameter
B.4) Focal 4 migrations
Migration weight
0.5
0
10 s.e.
SLP
DPRG
DPRN
CACN
KOOT
NHN
AHQT
CACG
MDENT
0.0
0.1
0.2
0.3
0.4
Drift parameter
C.4) All samples 4 migrations
REM
LMC
NHN
KOOT
SF
DPRN
CACN
DPRG
SLP
SWB
DUN
PED
BOG
IM62
AHQT
CACG
MAR
YJS
MDENT
Migration weight
0.5
0
10 s.e.
0.000
0.005
0.010
0.015
Drift parameter
