# Supplementary material for: Speciation and Introgression between Mimulus nasutus and Mimulus guttatus
Source: PLoS Genet. 2014 Jun 26;10(6):e1004410. doi: 10.1371/journal.pgen.1004410 (PMC4072524; doi:10.1371/journal.pgen.1004410)
Supplement: Figure S11 — Histograms of pairwise sequence divergence to M. nasutus. Shown in overlapping windows of varying size, by individual M. guttatus sample. (A–F) show window sizes from 1 kb to 100 kb. Dotted lines denote πS<0.5%. (PDF) [file pgen.1004410.s011.pdf]

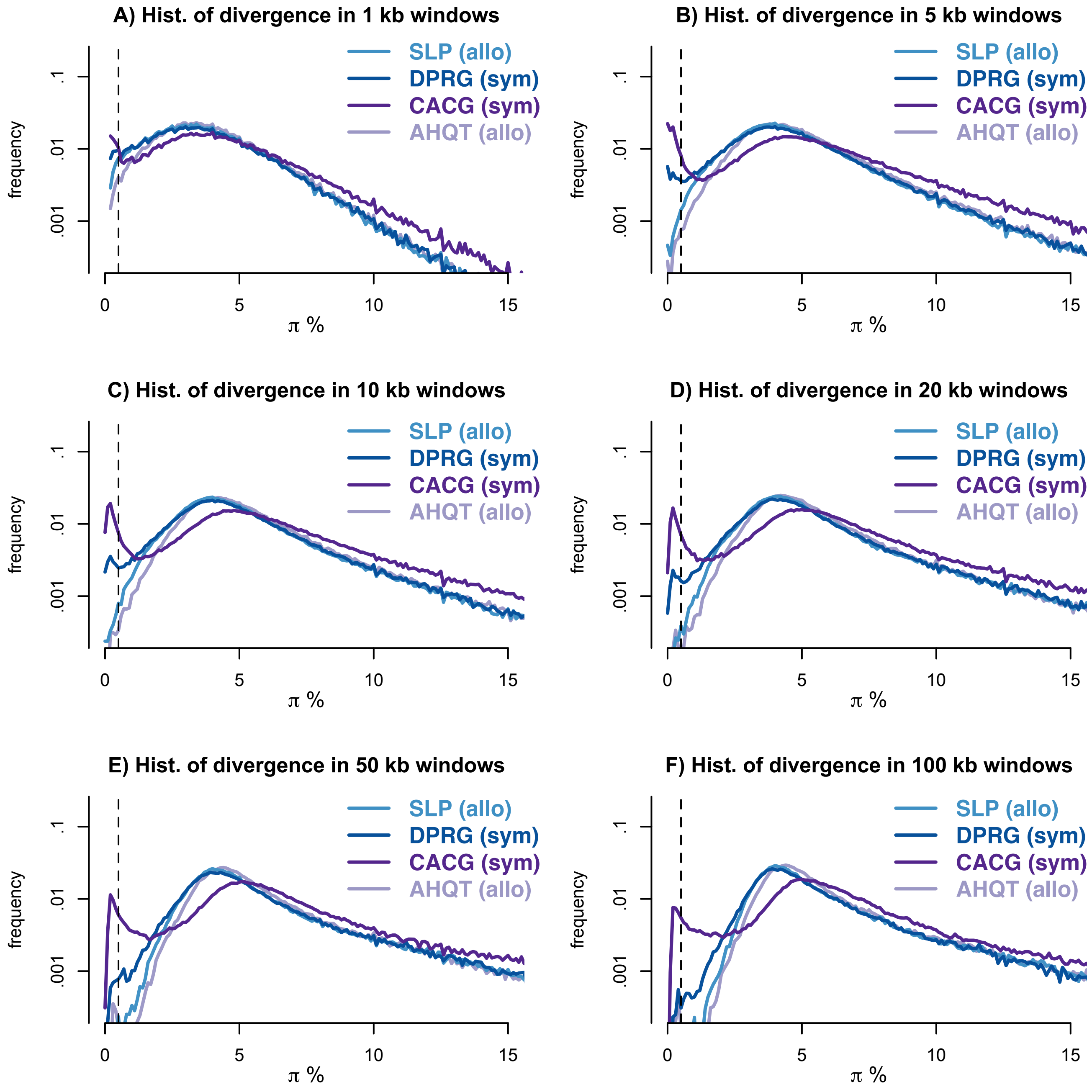
A) Hist. of divergence in 1 kb windows
B) Hist. of divergence in 5 kb windows
C) Hist. of divergence in 10 kb windows
D) Hist. of divergence in 20 kb windows
E) Hist. of divergence in 50 kb windows
F) Hist. of divergence in 100 kb windows
SLP (allo)
DPRG (sym)
CACG (sym)
AHQT (allo)
frequency
.1
.01
.001
π %
0
5
10
15
