# Supplementary material for: Speciation and Introgression between Mimulus nasutus and Mimulus guttatus
Source: PLoS Genet. 2014 Jun 26;10(6):e1004410. doi: 10.1371/journal.pgen.1004410 (PMC4072524; doi:10.1371/journal.pgen.1004410)
Supplement: Figure S12 — The admixture block length distribution under alternative post-hoc ‘healing’ rules. The number of admixed blocks (as inferred by a greater than 95% posterior probability of M. nasutus ancestry from our HMM) longer than x. We joined two admixture blocks within (A) 0, (B) 20, (C) 50, or (D) 100 kb. (PDF) [file pgen.1004410.s012.pdf]

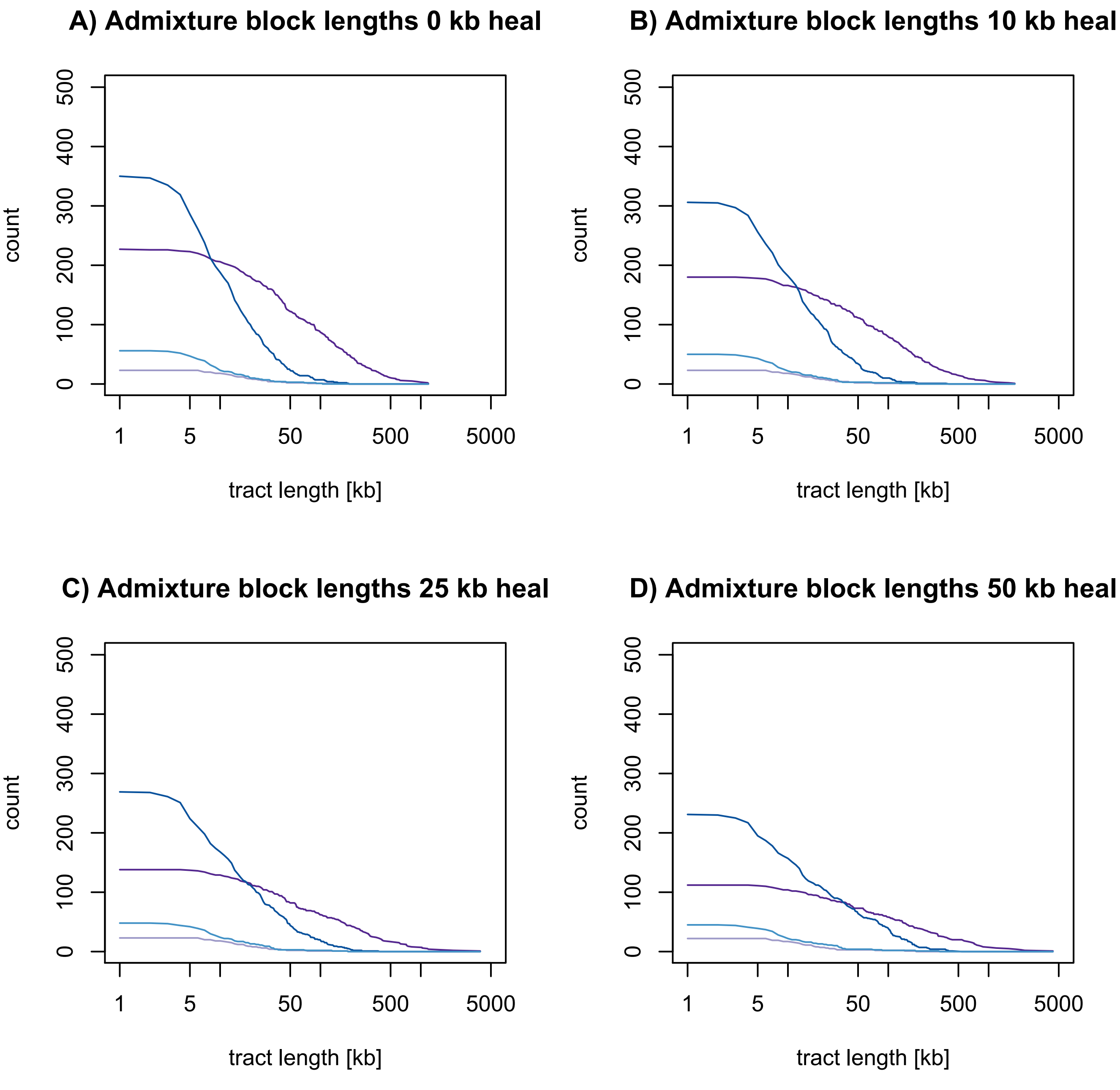

A) Admixture block lengths 0 kb heal
B) Admixture block lengths 10 kb heal
C) Admixture block lengths 25 kb heal
D) Admixture block lengths 50 kb heal
count
0
100
200
300
400
500
tract length [kb]
1
5
50
500
5000
