# Supplementary material for: Speciation and Introgression between Mimulus nasutus and Mimulus guttatus
Source: PLoS Genet. 2014 Jun 26;10(6):e1004410. doi: 10.1371/journal.pgen.1004410 (PMC4072524; doi:10.1371/journal.pgen.1004410)
Supplement: Table S1 — Detailed information about the biology, geography, inbreeding and sequencing of each sample analyzed in this manuscript. Gray-shaded rows denote focal samples used in our primary analyses. (DOCX) [file pgen.1004410.s017.docx]

*Table S1)* Detailed information about the biology, geography, inbreeding and sequencing of each sample analyzed in this manuscript.

| **ID** | ***Sp.*** | **State** | **(Long , Lat)** | **Ecology, etc.** | **Patry** | **LH ^+^** | **# Gen Inbred** | **Read**  **Length**^♬^ | **Million**  **Paired-**  **End**  **Reads** | **Machine**  **^€^** | **Seq.**  **Facility**  **^¥^** | **NCBI SRA Accession No.** |
| --- | --- | --- | --- | --- | --- | --- | --- | --- | --- | --- | --- | --- |
| DENT-13007 | *dent* | AZ | unknown^#^ | Inland | ? | PE | natural | 76 | 22.1 | GA-II | DoE JGI | SRX030541 |
| AHQT1.2 | *gutt* | WY | (-110.813, 44.431) | Inland, thermal. *M. guttatus* pop. with a high selfing rate | allo | AN | 1 | 100 | 55.8 | Hi-Seq | DoE JGI | SRX142379 |
| BOG10 | *gutt* | NV | (-118.805, 41.923) | Inland, hot springs | allo | PE | 3 | 76 | 28.0 | GA-II | DoE JGI | SRX030570 |
| CACG6 | *gutt* | WA | (-121.366, 45.710) | Inland | sym | AN | 3 | 100 | 85.2 | Hi-Seq | IGSP | SRX525044 |
| DPRG84* | *gutt* | CA | (-120.344, 37.828) | Inland | sym | AN | 3 | 100 | 64.2 | Hi-Seq | DoE JGI | SRA166889 alias MED82 |
| DUN | *gutt* | OR | (-124.137, 43.893) | Coastal, dunes | allo | PE | >6 | 36 | 26.2 | GA-II | DoE JGI | SRX030973 SRX030974 |
| IM62 | *gutt* | OR | (-122.108, 44.481) | Inland | allo | AN | >10 | 100 | 103.2 | Hi-Seq | DoE JGI | SRX115898 |
| IM62^$^ | *gutt* | OR | (-122.108, 44.481) | Inland | allo | AN | >10 | 75 | 24.9 | GA-II | DoE JGI | SRX021072 |
| LMC24 | *gutt* | CA | (-123.084, 38.864) | Inland | allo | AN | 4 | 76 | 24.8 | GA-II | DoE JGI | SRX030680 |
| MAR3 | *gutt* | OR | (-123.294, 43.479) | Inland | allo | AN | 3 | 76 | 30.6 | GA-II | DoE JGI | SRX030542 |
| PED5 | *gutt* | AZ | (-110.130, 31.587) | Inland | allo | ? | 2 | 76 | 25.6 | GA-II | DoE JGI | SRX030544 |
| REM8-10 | *gutt* | CA | (-122.411, 38.860) | Inland, serpentine | allo | AN | 4 | 76 | 27.0 | GA-II | DoE JGI | SRX030546 |
| SLP19 | *gutt* | CA | (-120.462, 37.848) | Inland, serpentine | para | AN | ≥3 | 100 | 61.1 | Hi-Seq | DoE JGI | SRX142377 |
| SWB-S3-1-8 | *gutt* | CA | (-123.690, 39.036) | Coastal | allo | PE | 10 | 76 | 28.6 | GA-II | DoE JGI | SRX030679 |
| YJS6 | *gutt* | ID | (-114.585, 44.951) | Inland | allo | PE | 3 | 76 | 25.2 | GA-II | DoE JGI | SRX030545 |
| CACN9 | *nas* | WA | (-121.367, 45.711) | Inland, seep | sym | AN | natural | 100 | 58.4 | Hi-Seq | IGSP | SRX525048 |
| DPRN104* | *nas* | CA | (-120.344, 37.829) | Inland | sym | AN | natural | 100 | 53.2 | Hi-Seq | IGSP | SRX525050 |
| KOOT | *nas* | MT | (-115.983, 48.104) | Inland | allo | AN | natural | 100 | 57.2 | Hi-Seq | IGSP | SRX525049 |
| NHN | *nas* | VI^$^ | (-124.160, 49.273) | Coastal, meadow | sym | AN | natural | 100 | 55.2 | Hi-Seq | IGSP | SRX525051 |
| SF5 | *nas* | OR | (-121.022, 45.264) | Inland | allo | AN | natural | 76 | 24.2 | GA-II | DoE JGI | SRX116529 |

* DPRG and DPRN are referred to as MED and MEN in previous publications, respectively.

$ This is a technical replicate. We present comparisons to this sample in Table S2A. Otherwise, we do not use this sample in our analyses.

+ LH = Life History: AN = annual PE = perennial

# Maricopa County, AZ, USA

^$^ Vancouver Island, Canada

^♬^Read length in base-pairs of a single end of paired end reads.

**^€^**Sequencing Technology - Hi-Seq = Illumina Hi-Seq 2000; GA-II = Illumina Genome Analyzer II

**^¥^** Sequencing facility - DoE JGI = U.S. Department of Energy Joint Genome Institute; IGSP = Duke Institute for Genome Sciences and Policy
