# Supplementary material for: Speciation and Introgression between Mimulus nasutus and Mimulus guttatus
Source: PLoS Genet. 2014 Jun 26;10(6):e1004410. doi: 10.1371/journal.pgen.1004410 (PMC4072524; doi:10.1371/journal.pgen.1004410)
Supplement: Table S2 — Pairwise sequence comparisons between samples. (A) For all pairwise comparisons, we report the identifiers of our samples, the mean number of pairwise sequence differences at fourfold degenerate sites (πS), the ratio of diversity at fully constrained and fourfold degenerate sites (πN/πS), the type of comparison (with regard to the population and species sample), and the number of focal samples involved in the comparison. (B) All pairwise πS and πN/πS values for focal samples as obtained from bwa and (Stampy) between focal comparisons. (DOCX) [file pgen.1004410.s018.docx]

*Table S2*) Pairwise sequence comparisons between samples. *S2A*) Pairwise comparisons between all samples (bwa pipeline). *S2B*) Pairwise comparisons between focal samples (comparing stampy and bwa pipelines)

**Table S2A**

| Sample 1 | Sample 2 | π_S_ | π_N_/π_S_ | Type of comparison | # Focal |
| --- | --- | --- | --- | --- | --- |
| CACN | KOOT | 0.0091 | 0.2058 | M. nasutus | 2 |
| CACN | DPRN | 0.009 | 0.1946 | M. nasutus | 2 |
| CACN | NHN | 0.0112 | 0.1928 | M. nasutus | 2 |
| KOOT | DPRN | 0.0083 | 0.2031 | M. nasutus | 2 |
| KOOT | NHN | 0.009 | 0.1989 | M. nasutus | 2 |
| DPRN | NHN | 0.0108 | 0.1898 | M. nasutus | 2 |
| AHQT | CACN | 0.0544 | 0.1563 | M. nasutus X M. guttatus (North) | 2 |
| AHQT | KOOT | 0.0547 | 0.1578 | M. nasutus X M. guttatus (North) | 2 |
| AHQT | DPRN | 0.0539 | 0.155 | M. nasutus X M. guttatus (North) | 2 |
| AHQT | NHN | 0.0537 | 0.1548 | M. nasutus X M. guttatus (North) | 2 |
| CACG | CACN | 0.0448 | 0.1611 | M. nasutus X M. guttatus (North) | 2 |
| CACG | KOOT | 0.0459 | 0.1634 | M. nasutus X M. guttatus (North) | 2 |
| CACG | DPRN | 0.0453 | 0.1602 | M. nasutus X M. guttatus (North) | 2 |
| CACG | NHN | 0.0456 | 0.1601 | M. nasutus X M. guttatus (North) | 2 |
| CACN | DPRG | 0.0478 | 0.1566 | M. nasutus X M. guttatus (South) | 2 |
| CACN | SLP | 0.0507 | 0.1575 | M. nasutus X M. guttatus (South) | 2 |
| KOOT | DPRG | 0.0481 | 0.1576 | M. nasutus X M. guttatus (South) | 2 |
| KOOT | SLP | 0.0508 | 0.1589 | M. nasutus X M. guttatus (South) | 2 |
| DPRG | DPRN | 0.0473 | 0.154 | M. nasutus X M. guttatus (South) | 2 |
| DPRG | NHN | 0.0475 | 0.1544 | M. nasutus X M. guttatus (South) | 2 |
| DPRN | SLP | 0.0501 | 0.1562 | M. nasutus X M. guttatus (South) | 2 |
| NHN | SLP | 0.0503 | 0.1561 | M. nasutus X M. guttatus (South) | 2 |
| AHQT | CACG | 0.0398 | 0.161 | M. guttatus (North) | 2 |
| AHQT | DPRG | 0.0522 | 0.1542 | M. guttatus (South X North) | 2 |
| AHQT | SLP | 0.0534 | 0.1569 | M. guttatus (South X North) | 2 |
| CACG | DPRG | 0.0514 | 0.1579 | M. guttatus (South X North) | 2 |
| CACG | SLP | 0.0532 | 0.1593 | M. guttatus (South X North) | 2 |
| DPRG | SLP | 0.0446 | 0.1537 | M. guttatus (South) | 2 |
| CACN | SF | 0.0063 | 0.216 | M. nasutus | 1 |
| KOOT | SF | 0.0073 | 0.2205 | M. nasutus | 1 |
| DPRN | SF | 0.008 | 0.2037 | M. nasutus | 1 |
| NHN | SF | 0.01 | 0.1953 | M. nasutus | 1 |
| CACN | MDENT | 0.0647 | 0.1576 | M. nasutus X M. dentilobus | 1 |
| KOOT | MDENT | 0.0649 | 0.1585 | M. nasutus X M. dentilobus | 1 |
| MDENT | DPRN | 0.0641 | 0.1576 | M. nasutus X M. dentilobus | 1 |
| MDENT | NHN | 0.0641 | 0.1574 | M. nasutus X M. dentilobus | 1 |
| AHQT | SF | 0.0481 | 0.1547 | M. nasutus X M. guttatus (North) | 1 |
| BOG | CACN | 0.0503 | 0.1608 | M. nasutus X M. guttatus (North) | 1 |
| BOG | KOOT | 0.0505 | 0.1624 | M. nasutus X M. guttatus (North) | 1 |
| BOG | DPRN | 0.0496 | 0.1603 | M. nasutus X M. guttatus (North) | 1 |
| BOG | NHN | 0.0495 | 0.1601 | M. nasutus X M. guttatus (North) | 1 |
| CACG | SF | 0.0399 | 0.1612 | M. nasutus X M. guttatus (North) | 1 |
| CACN | IM62 | 0.0528 | 0.1563 | M. nasutus X M. guttatus (North) | 1 |
| CACN | IM62.rep | 0.0541 | 0.1564 | M. nasutus X M. guttatus (North) | 1 |
| CACN | MAR | 0.0536 | 0.1548 | M. nasutus X M. guttatus (North) | 1 |
| CACN | REM | 0.0453 | 0.1597 | M. nasutus X M. guttatus (North) | 1 |
| CACN | YJS | 0.0503 | 0.1592 | M. nasutus X M. guttatus (North) | 1 |
| IM62 | KOOT | 0.0532 | 0.1588 | M. nasutus X M. guttatus (North) | 1 |
| IM62 | DPRN | 0.0519 | 0.1544 | M. nasutus X M. guttatus (North) | 1 |
| IM62 | NHN | 0.0518 | 0.1546 | M. nasutus X M. guttatus (North) | 1 |
| IM62.rep | KOOT | 0.0544 | 0.1587 | M. nasutus X M. guttatus (North) | 1 |
| IM62.rep | DPRN | 0.0532 | 0.1546 | M. nasutus X M. guttatus (North) | 1 |
| IM62.rep | NHN | 0.0529 | 0.1545 | M. nasutus X M. guttatus (North) | 1 |
| KOOT | MAR | 0.0539 | 0.1571 | M. nasutus X M. guttatus (North) | 1 |
| KOOT | REM | 0.0453 | 0.1628 | M. nasutus X M. guttatus (North) | 1 |
| KOOT | YJS | 0.0504 | 0.1614 | M. nasutus X M. guttatus (North) | 1 |
| MAR | DPRN | 0.053 | 0.1543 | M. nasutus X M. guttatus (North) | 1 |
| MAR | NHN | 0.0528 | 0.1542 | M. nasutus X M. guttatus (North) | 1 |
| DPRN | REM | 0.0448 | 0.1592 | M. nasutus X M. guttatus (North) | 1 |
| DPRN | YJS | 0.0497 | 0.1585 | M. nasutus X M. guttatus (North) | 1 |
| NHN | REM | 0.045 | 0.159 | M. nasutus X M. guttatus (North) | 1 |
| NHN | YJS | 0.0495 | 0.1591 | M. nasutus X M. guttatus (North) | 1 |
| CACN | DUN | 0.0516 | 0.1543 | M. nasutus X M. guttatus (South) | 1 |
| CACN | LMC | 0.0435 | 0.1619 | M. nasutus X M. guttatus (South) | 1 |
| CACN | PED | 0.0467 | 0.1604 | M. nasutus X M. guttatus (South) | 1 |
| CACN | SWB | 0.0473 | 0.1594 | M. nasutus X M. guttatus (South) | 1 |
| DUN | KOOT | 0.0517 | 0.1559 | M. nasutus X M. guttatus (South) | 1 |
| DUN | DPRN | 0.0509 | 0.1532 | M. nasutus X M. guttatus (South) | 1 |
| DUN | NHN | 0.0508 | 0.1528 | M. nasutus X M. guttatus (South) | 1 |
| KOOT | LMC | 0.0435 | 0.1642 | M. nasutus X M. guttatus (South) | 1 |
| KOOT | PED | 0.0466 | 0.1629 | M. nasutus X M. guttatus (South) | 1 |
| KOOT | SWB | 0.0475 | 0.1612 | M. nasutus X M. guttatus (South) | 1 |
| LMC | DPRN | 0.0429 | 0.1627 | M. nasutus X M. guttatus (South) | 1 |
| LMC | NHN | 0.0428 | 0.1626 | M. nasutus X M. guttatus (South) | 1 |
| DPRG | SF | 0.0432 | 0.154 | M. nasutus X M. guttatus (South) | 1 |
| DPRN | PED | 0.0461 | 0.1591 | M. nasutus X M. guttatus (South) | 1 |
| DPRN | SWB | 0.0467 | 0.1591 | M. nasutus X M. guttatus (South) | 1 |
| NHN | PED | 0.046 | 0.1602 | M. nasutus X M. guttatus (South) | 1 |
| NHN | SWB | 0.0466 | 0.1583 | M. nasutus X M. guttatus (South) | 1 |
| SF | SLP | 0.0459 | 0.1564 | M. nasutus X M. guttatus (South) | 1 |
| AHQT | MDENT | 0.06 | 0.1613 | M. guttatus (North) X  M. dentilobus | 1 |
| CACG | MDENT | 0.0608 | 0.1621 | M. guttatus (North) X  M. dentilobus | 1 |
| MDENT | DPRG | 0.0646 | 0.1583 | M. guttatus (South) X  M. dentilobus | 1 |
| MDENT | SLP | 0.0647 | 0.1591 | M. guttatus (South) X  M. dentilobus | 1 |
| AHQT | BOG | 0.0357 | 0.1599 | M. guttatus (North) | 1 |
| AHQT | IM62 | 0.038 | 0.1568 | M. guttatus (North) | 1 |
| AHQT | IM62.rep | 0.0391 | 0.1573 | M. guttatus (North) | 1 |
| AHQT | MAR | 0.0349 | 0.1561 | M. guttatus (North) | 1 |
| AHQT | REM | 0.0456 | 0.1531 | M. guttatus (North) | 1 |
| AHQT | YJS | 0.0349 | 0.1591 | M. guttatus (North) | 1 |
| BOG | CACG | 0.0397 | 0.1624 | M. guttatus (North) | 1 |
| CACG | IM62 | 0.0413 | 0.1609 | M. guttatus (North) | 1 |
| CACG | IM62.rep | 0.0419 | 0.1628 | M. guttatus (North) | 1 |
| CACG | MAR | 0.0384 | 0.1597 | M. guttatus (North) | 1 |
| CACG | REM | 0.0453 | 0.1578 | M. guttatus (North) | 1 |
| CACG | YJS | 0.0394 | 0.1625 | M. guttatus (North) | 1 |
| AHQT | DUN | 0.0444 | 0.1529 | M. guttatus (South X North) | 1 |
| AHQT | LMC | 0.0434 | 0.158 | M. guttatus (South X North) | 1 |
| AHQT | PED | 0.0417 | 0.1582 | M. guttatus (South X North) | 1 |
| AHQT | SWB | 0.0426 | 0.1573 | M. guttatus (South X North) | 1 |
| BOG | DPRG | 0.0477 | 0.1582 | M. guttatus (South X North) | 1 |
| BOG | SLP | 0.049 | 0.1605 | M. guttatus (South X North) | 1 |
| CACG | DUN | 0.0468 | 0.156 | M. guttatus (South X North) | 1 |
| CACG | LMC | 0.0432 | 0.1611 | M. guttatus (South X North) | 1 |
| CACG | PED | 0.0435 | 0.1624 | M. guttatus (South X North) | 1 |
| CACG | SWB | 0.0443 | 0.1594 | M. guttatus (South X North) | 1 |
| IM62 | DPRG | 0.0515 | 0.1545 | M. guttatus (South X North) | 1 |
| IM62 | SLP | 0.0525 | 0.1569 | M. guttatus (South X North) | 1 |
| IM62.rep | DPRG | 0.0527 | 0.1545 | M. guttatus (South X North) | 1 |
| IM62.rep | SLP | 0.0541 | 0.1573 | M. guttatus (South X North) | 1 |
| MAR | DPRG | 0.0516 | 0.1528 | M. guttatus (South X North) | 1 |
| MAR | SLP | 0.0528 | 0.1558 | M. guttatus (South X North) | 1 |
| DPRG | REM | 0.042 | 0.152 | M. guttatus (South X North) | 1 |
| DPRG | YJS | 0.0477 | 0.1574 | M. guttatus (South X North) | 1 |
| REM | SLP | 0.0437 | 0.1549 | M. guttatus (South X North) | 1 |
| SLP | YJS | 0.0489 | 0.1595 | M. guttatus (South X North) | 1 |
| DUN | DPRG | 0.0488 | 0.1512 | M. guttatus (South) | 1 |
| DUN | SLP | 0.05 | 0.1548 | M. guttatus (South) | 1 |
| LMC | DPRG | 0.0409 | 0.1542 | M. guttatus (South) | 1 |
| LMC | SLP | 0.0425 | 0.1595 | M. guttatus (South) | 1 |
| DPRG | PED | 0.0435 | 0.1564 | M. guttatus (South) | 1 |
| DPRG | SWB | 0.044 | 0.1564 | M. guttatus (South) | 1 |
| PED | SLP | 0.0453 | 0.1598 | M. guttatus (South) | 1 |
| SLP | SWB | 0.0455 | 0.1599 | M. guttatus (South) | 1 |
| MDENT | SF | 0.0595 | 0.1604 | M. nasutus X M. dentilobus | 0 |
| BOG | SF | 0.0446 | 0.1599 | M. nasutus X M. guttatus (North) | 0 |
| IM62 | SF | 0.044 | 0.1523 | M. nasutus X M. guttatus (North) | 0 |
| IM62.rep | SF | 0.045 | 0.1538 | M. nasutus X M. guttatus (North) | 0 |
| MAR | SF | 0.0471 | 0.1532 | M. nasutus X M. guttatus (North) | 0 |
| REM | SF | 0.0412 | 0.1607 | M. nasutus X M. guttatus (North) | 0 |
| SF | YJS | 0.0446 | 0.1579 | M. nasutus X M. guttatus (North) | 0 |
| DUN | SF | 0.0461 | 0.1526 | M. nasutus X M. guttatus (South) | 0 |
| LMC | SF | 0.0388 | 0.1637 | M. nasutus X M. guttatus (South) | 0 |
| PED | SF | 0.042 | 0.1608 | M. nasutus X M. guttatus (South) | 0 |
| SF | SWB | 0.0424 | 0.1584 | M. nasutus X M. guttatus (South) | 0 |
| BOG | MDENT | 0.0558 | 0.1694 | M. guttatus (North) X  M. dentilobus | 0 |
| IM62 | MDENT | 0.054 | 0.1627 | M. guttatus (North) X  M. dentilobus | 0 |
| IM62.rep | MDENT | 0.0557 | 0.1615 | M. guttatus (North) X  M. dentilobus | 0 |
| MAR | MDENT | 0.0578 | 0.1618 | M. guttatus (North) X  M. dentilobus | 0 |
| MDENT | REM | 0.0581 | 0.1642 | M. guttatus (North) X  M. dentilobus | 0 |
| MDENT | YJS | 0.0567 | 0.1662 | M. guttatus (North) X  M. dentilobus | 0 |
| DUN | MDENT | 0.0599 | 0.1598 | M. guttatus (South) X  M. dentilobus | 0 |
| LMC | MDENT | 0.0574 | 0.1652 | M. guttatus (South) X  M. dentilobus | 0 |
| MDENT | PED | 0.0568 | 0.1637 | M. guttatus (South) X  M. dentilobus | 0 |
| MDENT | SWB | 0.0572 | 0.166 | M. guttatus (South) X  M. dentilobus | 0 |
| BOG | IM62 | 0.0345 | 0.1598 | M. guttatus (North) | 0 |
| BOG | IM62.rep | 0.0354 | 0.162 | M. guttatus (North) | 0 |
| BOG | MAR | 0.0345 | 0.161 | M. guttatus (North) | 0 |
| BOG | REM | 0.042 | 0.1631 | M. guttatus (North) | 0 |
| BOG | YJS | 0.0342 | 0.1662 | M. guttatus (North) | 0 |
| IM62 | IM62.rep | 0.0003 | 0.2433 | M. guttatus (North) | 0 |
| IM62 | MAR | 0.0319 | 0.1545 | M. guttatus (North) | 0 |
| IM62 | REM | 0.0421 | 0.1518 | M. guttatus (North) | 0 |
| IM62 | YJS | 0.0342 | 0.1564 | M. guttatus (North) | 0 |
| IM62.rep | MAR | 0.033 | 0.1553 | M. guttatus (North) | 0 |
| IM62.rep | REM | 0.0425 | 0.1551 | M. guttatus (North) | 0 |
| IM62.rep | YJS | 0.0353 | 0.1554 | M. guttatus (North) | 0 |
| MAR | REM | 0.0443 | 0.154 | M. guttatus (North) | 0 |
| MAR | YJS | 0.0355 | 0.1575 | M. guttatus (North) | 0 |
| REM | YJS | 0.0423 | 0.1595 | M. guttatus (North) | 0 |
| BOG | DUN | 0.0418 | 0.1568 | M. guttatus (South X North) | 0 |
| BOG | LMC | 0.0394 | 0.1663 | M. guttatus (South X North) | 0 |
| BOG | PED | 0.0379 | 0.1675 | M. guttatus (South X North) | 0 |
| BOG | SWB | 0.0395 | 0.162 | M. guttatus (South X North) | 0 |
| DUN | IM62 | 0.0433 | 0.1508 | M. guttatus (South X North) | 0 |
| DUN | IM62.rep | 0.0441 | 0.1512 | M. guttatus (South X North) | 0 |
| DUN | MAR | 0.0423 | 0.1525 | M. guttatus (South X North) | 0 |
| DUN | REM | 0.0391 | 0.156 | M. guttatus (South X North) | 0 |
| DUN | YJS | 0.0414 | 0.154 | M. guttatus (South X North) | 0 |
| IM62 | LMC | 0.0391 | 0.1569 | M. guttatus (South X North) | 0 |
| IM62 | PED | 0.0392 | 0.1572 | M. guttatus (South X North) | 0 |
| IM62 | SWB | 0.0397 | 0.1548 | M. guttatus (South X North) | 0 |
| IM62.rep | LMC | 0.0401 | 0.1589 | M. guttatus (South X North) | 0 |
| IM62.rep | PED | 0.0403 | 0.156 | M. guttatus (South X North) | 0 |
| IM62.rep | SWB | 0.0409 | 0.1544 | M. guttatus (South X North) | 0 |
| LMC | MAR | 0.042 | 0.1577 | M. guttatus (South X North) | 0 |
| LMC | REM | 0.03 | 0.1767 | M. guttatus (South X North) | 0 |
| LMC | YJS | 0.0392 | 0.1668 | M. guttatus (South X North) | 0 |
| MAR | PED | 0.0409 | 0.1578 | M. guttatus (South X North) | 0 |
| MAR | SWB | 0.0416 | 0.1555 | M. guttatus (South X North) | 0 |
| PED | REM | 0.0397 | 0.1628 | M. guttatus (South X North) | 0 |
| PED | YJS | 0.0382 | 0.1641 | M. guttatus (South X North) | 0 |
| REM | SWB | 0.0349 | 0.1671 | M. guttatus (South X North) | 0 |
| SWB | YJS | 0.0388 | 0.1614 | M. guttatus (South X North) | 0 |
| DUN | LMC | 0.0372 | 0.1621 | M. guttatus (South) | 0 |
| DUN | PED | 0.0409 | 0.1566 | M. guttatus (South) | 0 |
| DUN | SWB | 0.0277 | 0.1661 | M. guttatus (South) | 0 |
| LMC | PED | 0.0376 | 0.1667 | M. guttatus (South) | 0 |
| LMC | SWB | 0.032 | 0.1732 | M. guttatus (South) | 0 |
| PED | SWB | 0.0372 | 0.1649 | M. guttatus (South) | 0 |

**Table S2B**

| Sample 1 | Sample 2 | π_S_ bwa  (π_S_ stampy) | π_N_/π_S_ bwa  (π_N_/π_S_ stampy) | Type of comparison | Rank π_S_  bwa (Stampy) |
| --- | --- | --- | --- | --- | --- |
| CACN | KOOT | 0.0091 (0.01159) | 0.2058 (0.23156) | M. nas | 25 (26) |
| CACN | DPRN | 0.009 (0.01159) | 0.1946 (0.2243) | M. nas | 26 (26) |
| CACN | NHN | 0.0112 (0.01408) | 0.1928 (0.21962) | M. nas | 23 (23) |
| KOOT | DPRN | 0.0083 (0.01086) | 0.2031 (0.23253) | M. nas | 28 (28) |
| KOOT | NHN | 0.009 (0.01162) | 0.1989 (0.22566) | M. nas | 26 (25) |
| DPRN | NHN | 0.0108 (0.01381) | 0.1898 (0.21745) | M. nas | 24 (24) |
| CACG | CACN | 0.0448 (0.05063) | 0.1611 (0.1706) | M. nas X M. gut  (North Sym) | 20 (21) |
| CACG | KOOT | 0.0459 (0.05156) | 0.1634 (0.17198) | M. nas X M. gut  (North Sym) | 17 (19) |
| CACG | DPRN | 0.0453 (0.05179) | 0.1602 (0.17097) | M. nas X M. gut  (North Sym) | 19 (18) |
| CACG | NHN | 0.0456 (0.05214) | 0.1601 (0.17137) | M. nas X M. gut  (North Sym) | 18 (17) |
| DPRG | CACN | 0.0478 (0.05417) | 0.1566 (0.16545) | M. nas X M. gut  (South Sym) | 14 (15) |
| DPRG | KOOT | 0.0481 (0.05426) | 0.1576 (0.16579) | M. nas X M. gut  (South Sym) | 13 (14) |
| DPRG | DPRN | 0.0473 (0.05415) | 0.154 (0.16538) | M. nas X M. gut  (South Sym) | 16 (16) |
| DPRG | NHN | 0.0475 (0.05433) | 0.1544 (0.16551) | M. nas X M. gut  (South Sym) | 15 (13) |
| SLP | DPRN | 0.0501 (0.0574) | 0.1562 (0.16762) | M. nas X M. gut  (South Allo) | 12 (11) |
| SLP | NHN | 0.0503 (0.05751) | 0.1561 (0.16776) | M. nas X M. gut  (South Allo) | 11 (9) |
| SLP | CACN | 0.0507 (0.05742) | 0.1575 (0.16782) | M. nas X M. gut  (South Allo) | 10 (10) |
| SLP | KOOT | 0.0508 (0.05739) | 0.1589 (0.1685) | M. nas X M. gut  (South Allo) | 9 (12) |
| AHQT | CACN | 0.0544 (0.06168) | 0.1563 (0.16628) | M. nas X M. gut  (North Allo) | 2 (3) |
| AHQT | KOOT | 0.0547 (0.06171) | 0.1578 (0.16675) | M. nas X M. gut  (North Allo) | 1 (2) |
| AHQT | DPRN | 0.0539 (0.06183) | 0.155 (0.16619) | M. nas X M. gut  (North Allo) | 3 (1) |
| AHQT | NHN | 0.0537 (0.0616) | 0.1548 (0.16609) | M. nas X M. gut  (North Allo) | 4 (4) |
| AHQT | CACG | 0.0398 (0.04527) | 0.161 (0.17154) | M. gut (North) | 22 (22) |
| AHQT | DPRG | 0.0522 (0.05915) | 0.1542 (0.16303) | M. gut (South X North) | 7 (7) |
| AHQT | SLP | 0.0534 (0.06082) | 0.1569 (0.16662) | M. gut (South X North) | 5 (5) |
| CACG | DPRG | 0.0514 (0.05757) | 0.1579 (0.16483) | M. gut (South X North) | 8 (8) |
| CACG | SLP | 0.0532 (0.05988) | 0.1593 (0.16779) | M. gut (South X North) | 6 (6) |
| DPRG | SLP | 0.0446 (0.05066) | 0.1537 (0.16331) | M. gut (South) | 21 (20) |
