# Supplementary material for: Speciation and Introgression between Mimulus nasutus and Mimulus guttatus
Source: PLoS Genet. 2014 Jun 26;10(6):e1004410. doi: 10.1371/journal.pgen.1004410 (PMC4072524; doi:10.1371/journal.pgen.1004410)
Supplement: Table S3 — A summary of pairwise sequence diversity in comparisons between all samples. We split this summary by the number of focal samples and the class of population comparison, and present both the mean number of pairwise sequence differences at fourfold degenerate sites (πS), and the ratio of diversity at fully constrained and fourfold degenerate sites (πN/πS). (DOCX) [file pgen.1004410.s019.docx]

*Table S3)* A summary of pairwise sequence diversity in comparisons between all samples.

| Comparison | # of focal samples | π_S_ | π_N_/π_S_ |
| --- | --- | --- | --- |
| M. guttatus (North) | 0 | 0.032881114 | 0.158215329 |
| M. guttatus (North) | 1 | 0.03905176 | 0.159231447 |
| M. guttatus (North) | 2 | 0.03975362 | 0.160967574 |
| M. guttatus (North) X M. dentilobus | 0 | 0.056176252 | 0.163768857 |
| M. guttatus (North) X M. dentilobus | 1 | 0.060400169 | 0.161706935 |
| M. guttatus (South X North) | 0 | 0.040716802 | 0.156685768 |
| M. guttatus (South X North) | 1 | 0.048184677 | 0.156386183 |
| M. guttatus (South X North) | 2 | 0.052557792 | 0.157113957 |
| M. guttatus (South) | 0 | 0.034973709 | 0.163133394 |
| M. guttatus (South) | 1 | 0.046413063 | 0.155447195 |
| M. guttatus (South) | 2 | 0.044593728 | 0.153736849 |
| M. guttatus (South) X M. dentilobus | 0 | 0.058394551 | 0.162545546 |
| M. guttatus (South) X M. dentilobus | 1 | 0.064657437 | 0.158693178 |
| M. nasutus | 1 | 0.007883245 | 0.207432369 |
| M. nasutus | 2 | 0.009565595 | 0.197091862 |
| M. nasutus X M. dentilobus | 0 | 0.059518884 | 0.160402991 |
| M. nasutus X M. dentilobus | 1 | 0.064453912 | 0.157786429 |
| M. nasutus X M. guttatus (North) | 0 | 0.044767503 | 0.155487085 |
| M. nasutus X M. guttatus (North) | 1 | 0.050955018 | 0.157330052 |
| M. nasutus X M. guttatus (North) | 2 | 0.049721701 | 0.158399731 |
| M. nasutus X M. guttatus (South) | 0 | 0.043482976 | 0.156725938 |
| M. nasutus X M. guttatus (South) | 1 | 0.047854641 | 0.157210047 |
| M. nasutus X M. guttatus (South) | 2 | 0.049053125 | 0.156452924 |
