# Supplementary material for: Speciation and Introgression between Mimulus nasutus and Mimulus guttatus
Source: PLoS Genet. 2014 Jun 26;10(6):e1004410. doi: 10.1371/journal.pgen.1004410 (PMC4072524; doi:10.1371/journal.pgen.1004410)
Supplement: Table S4 — Summary of the admixture block-length distribution and the robustness of our inference of admixture history. For each focal sample, we present (1) The inferred number of blocks of M. nasutus ancestry (2) The total length (in kb) of M. nasutus ancestry inferred in the focal sample, (3) The mean block length in centiMorgans = mean_block_length[bp] * total_map_length[cM]/genome_size[bp], where total_map_length ∼1,470 cM and total_genome_size ∼2.6*108. (4) The standard deviation in block length (in cM). (5) T, the expected number of generations since admixture = 1/mean_length(cM). (6) P(exp) – A one sided test of the hypothesis that our block length distribution is not more variable than expected under one admixture pulse at time T. We obtained this probability by resampling blocks with replacement 1,000 times and finding the proportion of resampling experiments with mean block lengths greater than the variance in block lengths. (7) The probability that ancestry from an admixture event at time T is maintained until the present. This low probability (never greater than 10−10), argues against a model of M. nasutus ancestry in CACG or in DPRG being derived from a small number of introgression events. We explored numerous post-hoc strategies to ensure the robustness of our inference to idiosyncrasies in the identification of contiguous admixture blocks. We first attempted to ‘heal’ physically close blocks into longer blocks. Specifically we connect admixture blocks separated by 0, 20, 50 or 100 kb into one longer block (noted in column, ‘Heal’). We also control for the potential of accidentally labeling regions of low divergence as introgressed by removing blocks whose length matches those identified in our allopatric samples, SLP or AHQT (noted in the column, ‘Control for short blocks’). (DOCX) [file pgen.1004410.s020.docx]

*Table S4)* Summary of the admixture block-length distribution and the robustness of our inference of admixture history.

| Focal Sample | Control for short blocks | Heal | # of blocks | Length (kb) | Mean  Length (cM) | Sdv length (cM) | ***T*** - Time since admixture | P(exp) | Prob (block inherited from point event) |
| --- | --- | --- | --- | --- | --- | --- | --- | --- | --- |
| CACG | none | 0 | 227 | 29977 | 0.74 | 1.04 | 135 | <0.001 | 9.40E-38 |
|  |  | 20 | 180 | 30554 | 0.95 | 1.45 | 105 | <0.001 | 7.68E-29 |
|  |  | 50 | 138 | 32031 | 1.3 | 2.5 | 77 | <0.001 | 1.75E-20 |
|  |  | 100 | 112 | 33873 | 1.7 | 3.19 | 59 | <0.001 | 3.20E-15 |
|  | ahqt | 0 | 204 | 29352 | 0.81 | 1.08 | 124 | <0.001 | 1.88E-34 |
|  |  | 20 | 157 | 29928 | 1.07 | 1.51 | 93 | <0.001 | 1.98E-25 |
|  |  | 50 | 115 | 31412 | 1.53 | 2.68 | 65 | <0.001 | 4.43E-17 |
|  |  | 100 | 90 | 33182 | 2.07 | 3.45 | 48 | <0.001 | 4.06E-12 |
|  | Slp | 0 | 171 | 29021 | 0.95 | 1.12 | 105 | 0.03 | 7.58E-29 |
|  |  | 20 | 130 | 29450 | 1.27 | 1.59 | 79 | 0.04 | 4.85E-21 |
|  |  | 50 | 90 | 30770 | 1.92 | 2.91 | 52 | 0.04 | 3.16E-13 |
|  |  | 100 | 67 | 32235 | 2.7 | 3.81 | 37 | 0.06 | 7.88E-09 |
| DPRG | None | 0 | 350 | 6484 | 0.1 | 0.13 | 962 | <0.001 | 6.89E-286 |
|  |  | 20 | 306 | 6979 | 0.13 | 0.19 | 781 | <0.001 | 1.31E-231 |
|  |  | 50 | 269 | 8194 | 0.17 | 0.25 | 585 | <0.001 | 1.26E-172 |
|  |  | 100 | 231 | 11101 | 0.27 | 0.39 | 371 | <0.001 | 2.47E-108 |
|  | Ahqt | 0 | 327 | 5848 | 0.1 | 0.13 | 997 | <0.001 | 2.87E-296 |
|  |  | 20 | 283 | 6348 | 0.13 | 0.18 | 795 | <0.001 | 1.51E-235 |
|  |  | 50 | 246 | 7560 | 0.17 | 0.25 | 580 | <0.001 | 4.44E-171 |
|  |  | 100 | 209 | 10412 | 0.28 | 0.4 | 358 | <0.001 | 2.12E-104 |
|  | Slp | 0 | 294 | 5622 | 0.11 | 0.14 | 932 | <0.001 | 7.28E-277 |
|  |  | 20 | 256 | 6041 | 0.13 | 0.19 | 755 | 0.02 | 9.58E-224 |
|  |  | 50 | 221 | 7212 | 0.18 | 0.26 | 546 | <0.001 | 6.25E-161 |
|  |  | 100 | 186 | 9855 | 0.3 | 0.4 | 336 | <0.001 | 5.41E-98 |
| SLP | none | 0 | 56 | 867 | 0.09 | 0.11 | 1151 | 0.1 | 0 |
|  |  | 20 | 50 | 932 | 0.1 | 0.18 | 956 | 0.11 | 4.09E-284 |
|  |  | 50 | 48 | 982 | 0.11 | 0.18 | 871 | 0.11 | 1.43E-258 |
|  |  | 100 | 45 | 1192 | 0.15 | 0.3 | 673 | 0.06 | 5.60E-199 |
|  | ahqt | 0 | 33 | 330 | 0.06 | 0.09 | 1782 | 0.37 | 0 |
|  |  | 20 | 27 | 331 | 0.07 | 0.14 | 1454 | 0.34 | 0 |
|  |  | 50 | 25 | 376 | 0.08 | 0.14 | 1185 | 0.3 | 0 |
|  |  | 100 | 23 | 559 | 0.14 | 0.36 | 733 | 0.17 | 3.80E-217 |
| AHQT | none | 0 | 23 | 630 | 0.15 | 0.21 | 651 | 0.37 | 2.55E-192 |
|  |  | 20 | 23 | 630 | 0.15 | 0.21 | 651 | 0.37 | 2.55E-192 |
|  |  | 50 | 23 | 630 | 0.15 | 0.21 | 651 | 0.34 | 2.55E-192 |
|  |  | 100 | 22 | 689 | 0.18 | 0.26 | 569 | 0.12 | 8.10E-168 |
