# Supplementary material for: Speciation and Introgression between Mimulus nasutus and Mimulus guttatus
Source: PLoS Genet. 2014 Jun 26;10(6):e1004410. doi: 10.1371/journal.pgen.1004410 (PMC4072524; doi:10.1371/journal.pgen.1004410)
Supplement: Table S5 — Inference of introgression from M. guttatus to M. nasutus is robust to alternative thresholds of identifying outlier regions. The number of genomic regions where the outlier M. nasutus sample (or collection of samples) is closer to the M. guttatus sample of interest than is the average M. nasutus sample is presented before the slash and the total number of outlier regions with informative data is given after the slash. Note that the total number of outlier regions for a given M. nasutus sample may differ in comparisons to AHQT and SLP due to different patterns of missing data. πS and L denote the threshold πS and the sliding window size used to identity 20 kb outlier regions, respectively (see Text S1 for more details). Light gray shading represents the intermediate parameter values for identifying outliers regions reported in the main text. (DOCX) [file pgen.1004410.s021.docx]

*Table S5)* Inference of introgression from *M. guttatus* to *M. nasutus* is robust to alternative thresholds of identifying outlier regions.

| **A** | ***L*** | π_S_% | DPRN | NHN | KOOT | CACN | Northern_nas |
| --- | --- | --- | --- | --- | --- | --- | --- |
| To AHQT | 5 | 0.5 | 47/102 (0.814) | **138/231 (0.002)** | 31/60 (0.449) | 101/207 (0.662) | **270/498 (0.033)** |
|  | 5 | 1.0 | 40/80 (0.544) | **120/197 (0.001)** | 25/53 (0.708) | 81/154 (0.286) | **226/404 (0.01)** |
|  | 5 | 2.0 | 28/56 (0.317) | **90/133 (0.02)** | 17/38 (0.76) | 59/106 (0.636) | 166/277 (0.144) |
|  | 10 | 0.5 | 71/154 (0.853) | **164/290 (0.015)** | 39/80 (0.631) | 123/243 (0.449) | 326/613 (0.062) |
|  | 10 | 1.0 | 68/134 (0.466) | **141/249 (0.021)** | 36/67 (0.313) | 95/174 (0.128) | **272/490 (0.008)** |
|  | 10 | 2.0 | 43/89 (0.664) | 90/165 (0.138) | 32/54 (0.11) | 61/116 (0.321) | **183/335 (0.05)** |
|  | 20 | 0.5 | 126/247 (0.4) | **245/453 (0.045)** | 40/105 (0.995) | 186/348 (0.109) | 471/906 (0.122) |
|  | 20 | 1.0 | 112/216 (0.317) | **222/402 (0.02)** | 46/98 (0.76) | 146/297 (0.636) | 414/797 (0.144) |
|  | 20 | 2.0 | 78/139 (0.087) | 147/273 (0.113) | 42/77 (0.247) | 107/198 (0.143) | **296/548 (0.033)** |
| **B** | ***L*** | π_S_ | DPRN | NHN | KOOT | CACN | Northern_nas |
| To SLP | 5 | 0.5 | 36/71 (0.5) | 85/172 (0.59) | 13/32 (0.892) | 50/111 (0.873) | 148/315 (0.87) |
|  | 5 | 1.0 | 27/56 (0.656) | 72/136 (0.274) | 17/34 (0.568) | 37/79 (0.75) | 126/249 (0.45) |
|  | 5 | 2.0 | 16/39 (0.432) | 49/91 (0.596) | 13/24 (0.551) | 26/52 (0.95) | 88/167 (0.872) |
|  | 10 | 0.5 | 48/99 (0.656) | 112/217 (0.342) | 18/47 (0.96) | 72/153 (0.79) | 202/417 (0.754) |
|  | 10 | 1.0 | 42/82 (0.456) | 97/186 (0.304) | 19/46 (0.908) | 51/108 (0.75) | 167/340 (0.648) |
|  | 10 | 2.0 | 22/52 (0.894) | 62/124 (0.536) | 17/33 (0.5) | 34/68 (0.548) | 113/225 (0.5) |
|  | 20 | 0.5 | 73/159 (0.867) | 156/305 (0.366) | 23/61 (0.98) | 111/224 (0.579) | 290/590 (0.675) |
|  | 20 | 1.0 | 70/137 (0.432) | 133/269 (0.596) | 30/60 (0.551) | 87/196 (0.95) | 250/525 (0.872) |
|  | 20 | 2.0 | 45/91 (0.583) | 90/180 (0.53) | 24/48 (0.557) | 44/111 (0.989) | 158/339 (0.904) |
