# Supplementary material for: Speciation and Introgression between Mimulus nasutus and Mimulus guttatus
Source: PLoS Genet. 2014 Jun 26;10(6):e1004410. doi: 10.1371/journal.pgen.1004410 (PMC4072524; doi:10.1371/journal.pgen.1004410)
Supplement: Table S6 — The negative relationship between recombination rate and divergence between M. nasutus and sympatric M. guttatus is not driven by sequencing depth or divergence to M. dentilobus. The first two columns present the percentage of fourfold degenerate sites that differ in the comparison (noted in the row heading) in regions with lower (low rec) and higher (high rec) than median recombination rates. The subsequent columns describe Spearman's ρ and the P-value associated with this nonparametric correlation coefficient with alternative controls described in the supplement. (DOCX) [file pgen.1004410.s022.docx]

*Table S6)* The negative relationship between recombination rate and divergence between *M. nasutus* and sympatric *M. guttatus* is not driven by sequencing depth or divergence to *M. dentilobus*.

| Differentiation by recombination rate | Mean # of pairwise sequence differences (%) | | No control | | Control for mutation (divergence to M. dent) | | Control for depth  (at synonymous sites) | | Control for mutation  and depth | |
| --- | --- | --- | --- | --- | --- | --- | --- | --- | --- | --- |
|  | low rec | high rec | P-value | ρ | P-value | ρ | P-value | ρ | P-value | ρ |
| Within  *M. nasutus* | 0.70 | 0.81 | 0.1228 | 0.0370 | 0.2553 | 0.0273 | 0.1439 | 0.0350 | 0.2375 | 0.0283 |
| Within northern  *M. guttatus* | 4.03 | 4.08 | 0.6744 | -0.0101 | 0.6764 | -0.0100 | 0.5734 | -0.0135 | 0.5865 | -0.0130 |
| Within southern  *M. guttatus* | 4.59 | 4.62 | 0.5353 | -0.0149 | 0.8785 | 0.0037 | 0.4509 | -0.0181 | 0.9571 | -0.0013 |
| Within all  *M. guttatus* | 5.17 | 5.09 | 0.2441 | -0.0279 | 0.6572 | -0.0106 | 0.2044 | -0.0304 | 0.5191 | -0.0155 |
| Between CACG  and *M. nasutus* | 5.48 | 5.14 | 0.0027 | -0.0718 | 0.0070 | -0.0646 | 0.0022 | -0.0732 | 0.0064 | -0.0652 |
| Between DPRG  and *M. nasutus* | 5.11 | 4.93 | 0.0008 | -0.0800 | 0.0022 | -0.0732 | 0.0006 | -0.0822 | 0.0016 | -0.0754 |
| Between SLP  and *M. nasutus* | 5.28 | 5.15 | 0.0297 | -0.0521 | 0.0458 | -0.0479 | 0.0241 | -0.0540 | 0.0354 | -0.0504 |
| Between AHQT  and *M. nasutus* | 5.63 | 5.54 | 0.2768 | -0.0261 | 0.5338 | -0.0149 | 0.2388 | -0.0282 | 0.4551 | -0.0179 |
