# Supplementary material for: Speciation and Introgression between Mimulus nasutus and Mimulus guttatus
Source: PLoS Genet. 2014 Jun 26;10(6):e1004410. doi: 10.1371/journal.pgen.1004410 (PMC4072524; doi:10.1371/journal.pgen.1004410)
Supplement: Table S7 — Read alignment. Number of reads aligned (at q29) with bwa and Stampy for our focal lines. (DOCX) [file pgen.1004410.s023.docx]

*Table S7)* Read alignment (at q29) with bwa and Stampy for our focal, high coverage lines.

| Line | Species | No. reads aligned: bwa | No. reads aligned: Stampy | No. reads aligned: Stampy − bwa | % Change in no. reads aligned: Stampy − bwa |
| --- | --- | --- | --- | --- | --- |
| AHQT | guttatus | 27,724,976 | 34,885,412 | 7,160,436 | 0.26 |
| CACG | guttatus | 54,760,211 | 69,720,097 | 14,959,886 | 0.27 |
| DPRG | guttatus | 31,136,751 | 44,130,212 | 12,993,461 | 0.42 |
| SLP | guttatus | 29,398,613 | 41,676,163 | 12,277,550 | 0.42 |
| CACN | nasutus | 30,503,460 | 46,031,138 | 15,527,678 | 0.51 |
| DPRN | nasutus | 27,449,620 | 42,950,405 | 15,500,785 | 0.56 |
| KOOT | nasutus | 38,523,447 | 59,276,013 | 20,752,566 | 0.54 |
| NHN | nasutus | 30,012,276 | 46,189,075 | 16,176,799 | 0.54 |
