# Supplementary material for: Speciation and Introgression between Mimulus nasutus and Mimulus guttatus
Source: PLoS Genet. 2014 Jun 26;10(6):e1004410. doi: 10.1371/journal.pgen.1004410 (PMC4072524; doi:10.1371/journal.pgen.1004410)
Supplement: Table S8 — After removing regions of recent introgression, interspecific divergence corresponds to the topology of the neighbor-joining tree. We present the percent of fourfold degenerate sites that differ between samples before (above the main diagonal) and after (below the main diagonal) removing regions inferred to be recently introgressed. Note that while CACG is much closer to M. nasutus samples before removing introgressed regions than AHQT, these two samples are equally differentiated from M. nasutus after removing regions of introgression. (DOCX) [file pgen.1004410.s024.docx]

*Table S8)* After removing regions of recent introgression, interspecific divergence corresponds to the topology of the neighbor-joining tree.

| Mean # of synonymous sequence diffs % | | | Including introgression regions | | | | | | | |
| --- | --- | --- | --- | --- | --- | --- | --- | --- | --- | --- |
|  |  |  | *M. guttatus* | | | | *M. nasutus* | | | |
|  |  |  | AHQT | CACG | DPRG | SLP | NHN | DPRN | KOOT | CACN |
| Excluding introgression | *M guttatus* | AHQT |  | 3.98 | 5.22 | 5.34 | 5.37 | 5.39 | 5.47 | 5.44 |
|  |  | CACG | 3.65 |  | 5.14 | 5.32 | 4.56 | 4.53 | 4.59 | 4.48 |
|  |  | DPRG | 5.26 | 5.29 |  | 4.46 | 4.75 | 4.73 | 4.81 | 4.78 |
|  |  | SLP | 5.37 | 5.42 | 4.48 |  | 5.03 | 5.01 | 5.08 | 5.07 |
|  | *M. nasutus* | NHN | 5.39 | 5.38 | 4.95 | 5.06 |  | 1.08 | 0.90 | 1.12 |
|  |  | DPRN | 5.41 | 5.40 | 4.93 | 5.05 | 1.08 |  | 0.83 | 0.90 |
|  |  | KOOT | 5.49 | 5.48 | 5.02 | 5.12 | 0.90 | 0.83 |  | 0.91 |
|  |  | CACN | 5.46 | 5.44 | 4.98 | 5.10 | 1.12 | 0.90 | 0.91 |  |
