# Supplementary material for: Speciation and Introgression between Mimulus nasutus and Mimulus guttatus
Source: PLoS Genet. 2014 Jun 26;10(6):e1004410. doi: 10.1371/journal.pgen.1004410 (PMC4072524; doi:10.1371/journal.pgen.1004410)
Supplement: Text S1 — Detailed descriptions of data generation, analyses, and tests of the robustness of major results outlined in the main text. This includes details of sequencing, alignment/read mapping, neighbor joining trees, PCA, and Treemix analyses. We also provide details of the implementation of our Hidden Markov Model, information regarding the recombination map used in our analyses, and our analysis of the relationship between local recombination rates and genetic variation. We include descriptions of additional PSMC results, including those between M. nasutus and sympatric M. nasutus. We evaluate the robustness of our major conclusions regarding introgression, by (1) providing additional Treemix analyses, (2) Analyzing block length distributions under alternative “healing” rules, and (3) Controlling for potential confounds of the observed relationship between pairwise sequence divergence and the local recombination rate. We also describe how the local recombination rate varies with genomic content. (DOCX) [file pgen.1004410.s025.docx]

**Text S1**

**Supplementary Methods**

*Sequencing:*

We generated new whole genome sequences for one *M. guttatus* (CACG) and four *M. nasutus* samples (CACN, DPRN, NHN, and KOOT). For these five samples, colleagues at Duke University extracted genomic DNA using a modified CTAB protocol ([1](#_ENREF_1)) and RNAse A treatment. Sequencing libraries were prepared at the Duke Institute for Genome Sciences and Policy (IGSP) using standard Illumina Tru-Seq DNA library preparation kits and protocols, and sequenced on the Illumina Hi-Seq 2000 platform at the IGSP. Before sequence analysis of all samples, we removed potential contamination of sequencing adapters and primers with Trimmomatic ([2](#_ENREF_2)) and confirmed removal using FastQC ([www.bioinformatics.babraham.ac.uk/projects/fastqc/](http://www.bioinformatics.babraham.ac.uk/projects/fastqc/)).

*Alignment processing*

After alignment, we removed potential pcr and optical duplicates using Picard (http://picard.sourceforge.net/). We did not filter reads with improper alignment flags (≤ ~5% of the mapped reads), however this had little effect on genotype calls (average proportion sequence difference between filtered and unfiltered datasets = 6.2 x 10^-5^ (± 1.3 x 10^-5^ SE) for five *Mimulus* lines varying in sequencing depth and read length). To minimize SNP errors around insertion/deletion polymorphisms, we performed local realignment for each sample using the Genome Analysis Tool Kit (GATK; [3](#_ENREF_3), [4](#_ENREF_4)).

*Downsampling for nj tree and PCA analyses:*

We use all nineteen samples for a genome-wide SNP analysis to learn about the genetic relationships and major components of genetic variation in these samples. For these analyses, we sampled 1,000 fourfold degenerate SNPs per chromosome (14,000 in total), which had at least two copies of the minor allele, and prioritized SNPs by the number of samples with available genotype data. For all loci we had sample data for at least 14 of 19 samples, and for 97% of loci, we had data for at least 16 of 19 samples. Coverage across these 14,000 SNPs ranged from 60% to 100% per sample. We resample these 14,000 SNPs with replacement to generate the distribution of trees presented in Figure 1B.

*PCA:*

We constructed a covariance matrix across pairs of individuals. To do so we evaluated the mean genotypic covariance across the 14,000 sites for a pair of samples. We calculated principle components as the eigenvectors of this matrix. Customized R scripts for this operation are available from the authors upon request.

*PSMC input file generation and bootstrap analyses:*

To create pseudo-diploid genomes for PSMC analyses ([5](#_ENREF_5)), we first called the consensus sequence for each of our lines by running SAMtools mpileup ([6](#_ENREF_6)) on the final, locally realigned bam file for each line. Due to differences in overall coverage among chromosomes, we set the minimum coverage to 5X for chromosomes 1, 2, 4, 6, 8, 10, 13 and 14, and 1X for chromosomes 3, 5, 7, 9, 11 and 12. For each line, we set the maximum coverage for all chromosomes to 2 times the standard deviation plus the mean. We merged consensus sequences using Heng Li’s seqtk toolset (https://github.com/lh3/seqtk), with a quality threshold of 20. For any site with residual heterozygosity, we randomly chose one allele.

To generate a measure of variability in the PSMC estimates of *M. guttatus* diversity and species divergence through time we ran 100 bootstrap analyses for each pairwise comparison. We used the PSMC utility splitfa to break up each pseudo-diploid genome into non-overlapping, similarly sized segments (resulting in 59 segments). To perform bootstrap analyses, we ran 100 separate PSMC analyses using the segmented genome as input and the –b (bootstrap) option. The bootstrap option randomly resamples with replacement from all of the segments to generate a unique/bootstrapped genome, similar in size to the original, and then runs PSMC on the bootstrapped genome. Note that bootstrapped genome sets were independent among different pairwise *Mimulus* comparisons. For our analyses we present both the point estimate using the full pseudo-diploid genome for each pairwise comparison (dark, thick lines) and the 100 bootstrap analyses (lighter, thin lines).

*Treemix analyses:*

Genotypes for these analyses consisted of the 14,000 biallelic SNPs used in our neighbor-joining and PCA analyses (above). We considered each line a population, with population allele counts being represented as ‘2,0’ or ‘0,2’ for the alternate genotypes, and ‘0,0’ as missing data.

*HMM to identify introgression in M. guttatus:*

To make appropriate emission probabilities for our HMM we need to generate a comparable distribution of pairwise comparisons within our four *M. nasutus* samples and between focal *M. guttatus* samples and the four *M. nasutus* samples. We also must acknowledge the heterogeneity in the density of called sites (*i.e.*, sites where both samples surpass our quality cutoffs) across the genome and across individuals. To even out sample size (because each *M. nasutus* could be compared to three other *M. nasutus* samples, while each *M. guttatus* sample could be compared to four *M. nasutus* samples), we alternately left out one *M. nasutus* sample in our calculation of π between an *M. guttatus* sample and the nearest *M. nasutus*. We combined all values across the 16 classes of comparisons (the product of four *M. guttatus* samples and the four ways to leave out one *M. nasutus* sample) to calculate the empirical distribution of π to the nearest *M. nasutus* sample.

To accommodate the heterogeneity in the number of called sites, we bin all pairwise comparisons in 1kb windows by the number of sites with data for both lines (greater than the smaller bounds and less than or equal to the larger 0,5,10,20,50,75,100,250). Within each window, there are 3 pairwise comparisons. Among these, we select the comparison with the lowest pairwise π that is also in the bin with the most sites. In practice, this usually amounts to selecting the lowest pairwise π in a window, because in 65% of windows all three pairwise comparisons to a focal individual are in the same bin. Nonetheless, we make note of the number of pairwise comparisons for each minimum π value and use this as a second layer of conditioning, below. For each set of conditioning we calculate the frequency of windows with π in given discretized bins.

With our distribution of π to the nearest *M. nasutus* samples in hand, we can now calculate emission probabilities. We do so for each 1kb window, conditional on the largest bin of called sites and the number of pairwise comparisons with this number of called sites. For a given *M. guttatus* sample, we systematically leave out one *M. nasutus* sample, looping over each *M. nasutus* sample. We then find the emission probabilities for *M. guttatus* or *M. nasutus* ancestry by finding the proportion of appropriately binned minimum π values in our within *M. nasutus* comparisons and the proportion of minimum π values in *M. guttatus* to *M. nasutus* comparisons, respectively. Finally, we average these emission probabilities across the four ways in which we left out a *M. nasutus* sample.

*Recombination map:*

In order to approximate the genetic distance per physical unit (cM/kb) of the IM62 *M. guttatus* v2.0 reference genome ([7](#_ENREF_7)), we accessed the map resources available at http://www.mimulusevolution.org. We began with the IMxIM map as an initial map because it contains linkage information from multiple individuals from the IM population. The IMxIM map also has the greatest number of mapped markers. To increase marker density we added markers from the two additional IMxSF maps not included in the IMxIM map. If flanking markers were shared between maps and if marker order was consistent, we assigned these additional markers a proportional genetic position in the IMxIM map according to their original recombination distance. We excluded entire regions where the genetic order of markers disagrees with the physical order of the reference genome, as well as regions distal to the first and last mapped marker on each chromosome; we did not estimate recombination in these regions or include them in our analyses with divergence. This conservative approach resulted in a final integrated map containing 285 markers with a total map length of ~14.7 Morgans (with genetic distances for ~256.5 Mb (87.5%) of the reference genome).

*Recombination rate and diversity (divergence):*

While calculating mean synonymous diversity in a window, we also calculate mean depth at synonymous sites and mean synonymous divergence to the outgroup, *M. dentilobus*. We then examine the spearman rank correlation of the local recombination rate and the residuals of the linear model where diversity is a function of divergence to *M. dentilobus* and mean depth at synonymous sites (Table S6).

**Supplementary Analyses and Results**

*Pairwise comparisons:*

Values of π_S_ and π_N_/π_S_ for each pairwise comparison are presented in Table S2. The mean number of pairwise sequence differences between *M. nasutus* and each focal *M. guttatus* sample is 4.54% (Northern sympatric – CACG), 4.76% (Southern sympatric – DPRG), 5.05% (Southern allopatric – SLP), 5.41% (Northern allopatric – AHQT).

To highlight the influence of read length and depth on estimates of diversity, in Table S3 we present mean π_S_ and π_N_/π_S_ values across all population comparisons split by the number of focal samples in a comparison (*i.e.*, zero means that neither of the samples is included in our detailed genomic analyses due to low depth or short reads). Note that for a given comparison between populations, diversity between two focal samples is much higher than that between two non-focal samples illustrating the influence of sequencing effort on diversity estimates. To avoid these effects we focus on our focal samples when discussing levels of diversity. We also note that we did not present in-depth genomic analyses of comparisons including the reference, IM62, because of unknown biases it may introduce.

We present values of pairwise π_S_ between each focal sample with (above the main diagonal) and without (below the main diagonal) putatively introgressed regions (as inferred by a >95% posterior probability of *M. nasutus* ancestry in a genomic region of an *M. guttatus* sample) in Table S8. Reassuringly, after removing such regions, our two northern focal *M. guttatus* samples no longer differ in the number of pairwise sequence difference to *M. nasutus*, suggesting that our HMM performed very well for CACG (compare CACG and AHQT to *M. nasutus* samples above and below the main diagonal). Removing regions of inferred recent introgression in DPRG also increased its distance from *M. nasutus* samples; however, this sample is still genetically closer to *M. nasutus* than is the allopatric southern sample, SLP. This suggests that our HMM may have missed short (*i.e.,* old) regions of introgression into DPRG and/or that even without introgression, DPRG is more closely related to the *M. nasutus* progenitor population than is SLP.

*Additional PSMC results:*

We present the results of additional PSMC analyses (on bwa-aligned data), including inference of divergence within and between species, *M. nasutus*’ population size decline, and effects of admixture on shared variation between *M. guttatus* and *M. nasutus* (Figures S2-S6)*.* We also include ‘zoomed-in’ and ‘zoomed-out’ views (changing the y-axis limits) for some analyses. In Figure S2, we present a ‘zoomed-out’ view of Figure 1E providing a view of historical population splits and population size changes over time. The extreme variation in recent population sizes demonstrates both the effect of population structure within *M. guttatus* on population size estimates, and the lower accuracy of PSMC time estimates in recent history ([5](#_ENREF_5)). In Figure S3, we present a ‘zoomed-in’ view of the split between *M. nasutus* and southern *M. guttatus*. The approximate split date of ~300-500 kya is visible by evaluating roughly when the southern *M. guttatus* x *M. nasutus* curve (SLP x KOOT, gray) diverges from the southern *M. guttatus* curve ([SLP x DPRG, blue; see 5](#_ENREF_5)).

We infer the history of population size decline in *M. nasutus* by running PSMC on all pairwise comparisons of our four high-coverage *M. nasutus* lines. From Figure S4, we observe that *M. nasutus*’ decline in effective population size was coincident with divergence between the species, indicating that it is plausible that the evolution of selfing was associated with speciation and the origin of *M. nasutus*. The extreme reduction in *M. nasutus*’ effective population size relative to *M. guttatus* is also evident from these analyses.

Our PSMC analyses demonstrate an effect of admixture on the inferred history of divergence. We observe a reduction in the between species effective population size between *M. nasutus* and sympatric *M. guttatus*, relative to that between *M. nasutus* and allopatric *M. guttatus* (Figure S5, and S6 for the full, zoomed-out view). In Figure S5, relatively recent (*i.e.*, between ~10 and 70 kya) effective population sizes between *M. nasutus* and sympatric *M. guttatus* are reduced relative to allopatric comparisons, and roughly in the range of or even lower than population sizes within southern and northern *M. guttatus*, further supporting a history of ongoing and recent introgression.

Finally, we present a set of Stampy-based PSMC trajectories overlaid with the original bwa-based PSMC trajectories & bootstraps (Figures S13-S16). PSMC trajectories for Stampy and bwa-processed data are largely similar. As expected from its larger estimate of π_s_ genome-wide, PSMC trajectories from Stampy alignments suggest larger absolute population sizes (greater absolute divergence). However, relative relationships and biological conclusions (including speciation up to ~500 kya, population size decline in *M. nasutus* since its origin, and effects of recent admixture on divergence) are unchanged.

For example, PSMC trajectories using Stampy-aligned data also find higher population divergence (population size) between northern and southern *M. guttatus* (AHQT x SLP) relative to that within either northern (AHQT x CACG) or southern *M. guttatus* (SLP x DPRG) (Figure S13). Although Stampy shows a spike in *M. nasutus* x *M. nasutus* population size in recent time, Stampy and bwa show extremely similar trajectories for *M. nasutus*’ population size decline since its origin (Figure S13). Similarly, divergence between *M. guttatus* and *M. nasutus* increases at roughly the same rate through time whether inference is made using bwa-aligned or Stampy-aligned data (Figure S14). Note that the absolute difference in divergence to *M. nasutus* between northern (AHQT) and southern (SLP) *M. guttatus* is similar in both data sets (*i.e.*, population size difference between black & gray lines and between red & purple lines is similar; Figure S14). Time since speciation inferred using Stampy-aligned data is also consistent (Figure S15). Note how the difference in divergence between vs. within species is similar for Stampy and bwa (*i.e.*, population size difference between gray & blue lines and between purple & brown lines in Figure S15 is similar). Lastly, we also see geography and admixture similarly impact PSMC inference of species divergence regardless of using Stampy or bwa-aligned data (Figure S16).

*Robustness of introgression results*

Treemix

We explored our Treemix analyses over a range of different sample subsets and numbers of admixture events:

***(A)*** Focal samples and the reference (IM62) rooted by the outgroup

***(B)*** Focal samples rooted by the outgroup

***(C)*** All *M. nasutus* and *M. guttatus* samples rooted by the outgroup.

For each set of samples, we allowed one, two, three, or four historical admixture events (Figure S10). Regardless of sample subset and the number of admixture events allowed, we always see strong evidence of introgression from *M. nasutus* into CACG, a result consistent with all analyses in this manuscript. However, the other clear signal of introgression observed in our genomic analyses – introgression from *M. nasutus* into DPRG, was only observed when we allow for more than one introgression event and analyze all focal samples and the reference genome (Figure S10 A.2-A.4). When we limit our analysis to focal samples (rooted by the outgroup) and allow for two or more introgression events, treemix places an introgression arrow from northern *M. guttatus* samples to SLP (Figure S10 B.2-B.4). We view this result as an attempt to explain the positive covariance in genotype between SLP and northern *M. guttatus* after accounting for topology; however, in this case, the direction of introgression is likely difficult to distinguish on the basis of the distance matrix and such a constrained topology of so few samples. Because other lines of evidence suggest introgression into DPRG, and because SLP and DPRG are equally diverged from northern M. guttatus after removing putatively introgressed genomic regions (Table S8), we interpret treemix results as consistent with introgression from M. nasutus into DPRG.

Block length distributions

In the main text, we used the length distribution of admixture blocks to provide a detailed view of the recent history of introgression of *M. nasutus* ancestry into *M. guttatus*. While this summary of the data contains much information, our inference of this distribution is likely imperfect. In practice, we may break up long admixture blocks or we may mislabel short genomic regions with low divergence as short admixture blocks.

In practice, both problems could confuse our inference. Miscalling short unadmixed regions and breaking up long regions into numerous smaller ones will both push back our inferred admixture time. Additionally, introducing short, false positive blocks may mislead us into seeing a mix of old and new admixture events, when in practice there was a single recent pulse. A major claim of our manuscript is that admixed *M. guttatus* samples are not simply early-generation hybrids, but rather represent on ongoing history of introgression. We therefore wish to ensure that these potential challenges to characterizing the block length distribution do not mislead our inference.

We use two strategies to ensure the robustness of our results. First, we ‘heal’ admixture blocks within X = {0,20,50,100} kb of one another (Figure S12), to guard against breaking up few long admixture blocks into more short ones. We also use our allopatric and putatively ‘pure’ *M. guttatus* samples to empirically control for the false positive admixture blocks. To do so, we alternatively use the block length distribution of AHQT and SLP and remove the closest matched block lengths in our other samples (note that we use the AHQT block length distribution in an attempt to better characterize the introgression history of SLP as well). By factorially implementing these controls, we see that our inference of ongoing introgression of *M. nasutus* into sympatric *M. guttatus* populations is robust. In all controls, we observe more variation in admixture tract lengths than would be expected under a simple point admixture model. Moreover, while removing young blocks and creating longer blocks creates a more recent estimated admixture time, our most recent estimated admixture time in CACG is 37 generations ago, arguing against a single recent admixture event. Even if admixture occurred 37 generations ago into CACG, it is very unlikely that a block from a given event at that time would survive to the present – and therefore gene flow is likely (relatively) consistently ongoing (Table S4).

Robustness of inferred introgression from *M. guttatus* into *M. nasutus*

In the main text we described our strategy of using outlier windows – regions where one *M. nasutus* sample differed radically from all others to infer historical introgression from *M. guttatus* into *M. nasutus*. The identification of outlier windows required numerous decisions; here we investigate the robustness of the signal of introgression to these choices.

The first was the π_S_ cutoff differentiating outlier and non-outlier regions. We chose three alternative values for this cutoff – 0.5% (roughly corresponding to the expected level of differentiation since the species split), 2.0% (roughly corresponding to expected levels of variation within an ancestral *M. guttatus* population), and 1.0% (representing a compromise between these values). Within a given cutoff, we identify 20 contiguous overlapping sliding windows (with a 1 kb slide) where one sample differs from all others by π_S_ greater than this threshold, while the others are differentiated from one another by π_S_ less than this threshold. Although we always insist on 20 contiguous windows (representing 20 kb), we vary the window size, allowing it to take the value of 5, 10 or 20 kb (noted by ***L*** in Table S5).

Regardless of exact thresholds, we always see evidence for either introgression into NHN and/or introgression into the pooled collection of northern *M. nasutus* samples (CACN, NHN, and KOOT), in the form of too many outlier windows being too close to AHQT (Table S5). By contrast, no samples are closer to SLP in outlier regions more often than expected by chance. However, as noted in the main text, our inability to identify introgression from southern M. *guttatus* into southern *M. nasutus* is likely underpowered because SLP may be too similar to the population that founded *M. nasutus*.

The relationship between divergence and recombination rate is not driven by sequencing depth or mutation rate variation

In the main text, we report a strong negative relationship between the local recombination rate (in 100 kb windows, smoothed over 500 kb) and absolute divergence between *M. nasutus* and sympatric *M. guttatus* samples at synonymous sites. We control for the potential confounds of the mutation rate (measured as divergence to *M. dentilobus*) and/or sequencing depth (at synonymous sites) in Table S6. To do so, we find the nonparametric correlation (Spearman’s ρ) between the recombination rate and residuals of predicted divergence given local depth and/or divergence to *M. dentilobus* (where predictions come from the best fit linear model).

The relationship between the local recombination rate and genomic content

Using the early release genome annotations available on phytozome [ftp://ftp.jgi-psf.org/pub/compgen/phytozome/v9.0/early_release/Mguttatus_v2.0/annotation/], and the genetic map described above, we examined the relationship between the locally smoothed recombination rate (in 100 kb windows, smoothed over 500 kb), and numerous genomic features – specifically, gene density, transposable element density, and the density of centromeric repeats (as defined in ([8](#_ENREF_8))). We found a negative correlation between the local recombination rate and both the number of transposable elements (rho= -0.305, P << 0.0001) and the number of centromeric repeats (rho = -0. 300, P << 0.0001), and a positive correlation between the local recombination rate and gene density (rho= 0.737, P << 0.0001). As described in the main text, this relationship seems incapable of driving the negative correlation between the recombination rate and synonymous divergence between *M. nasutus* and sympatric *M. guttatus*, without driving similar relationships within species or in allopatry.

**REFERENCES**

1. Kelly AJ & Willis JH (1998) Polymorphic microsatellite loci in Mimulus guttatus and related species. *Molecular ecology* 7(6):769-774.

2. Lohse M*, et al.* (2012) RobiNA: a user-friendly, integrated software solution for RNA-Seq-based transcriptomics. *Nucleic acids research* 40(W1):W622-W627.

3. DePristo MA*, et al.* (2011) A framework for variation discovery and genotyping using next-generation DNA sequencing data. *Nature genetics* 43(5):491-+.

4. McKenna A*, et al.* (2010) The Genome Analysis Toolkit: A MapReduce framework for analyzing next-generation DNA sequencing data. *Genome Res* 20(9):1297-1303.

5. Li H & Durbin R (2011) Inference of human population history from individual whole-genome sequences. *Nature* 475(7357):493-496.

6. Li H*, et al.* (2009) The Sequence Alignment/Map format and SAMtools. *Bioinformatics* 25(16):2078-2079.

7. Joint Genome Institute D (Mimulus Genome Project).

8. Fishman L & Saunders A (2008) Centromere-associated female meiotic drive entails male fitness costs in monkeyflowers. *Science* 322(5907):1559-1562.
